# A *GFP* splicing reporter in a *coilin* mutant background reveals links between alternative splicing, siRNAs, and coilin function in *Arabidopsis thaliana*

**DOI:** 10.1093/g3journal/jkad175

**Published:** 2023-08-04

**Authors:** Tatsuo Kanno, Phebe Chiou, Ming-Tsung Wu, Wen-Dar Lin, Antonius Matzke, Marjori Matzke

**Affiliations:** Institute of Plant and Microbial Biology, Academia Sinica, Taipei 115201, Taiwan; Institute of Plant and Microbial Biology, Academia Sinica, Taipei 115201, Taiwan; Institute of Plant and Microbial Biology, Academia Sinica, Taipei 115201, Taiwan; Genenet Technology (UK) Limited, 128 City Road, London EC1V 2NX, UK; Institute of Plant and Microbial Biology, Academia Sinica, Taipei 115201, Taiwan; Institute of Plant and Microbial Biology, Academia Sinica, Taipei 115201, Taiwan; Institute of Plant and Microbial Biology, Academia Sinica, Taipei 115201, Taiwan

**Keywords:** alternative splicing, *Arabidopsis thaliana*, Cajal bodies, coilin, GFP, MEM1, posttranscriptional gene silencing, small RNAs, SMU2, WRAP53, ZCH1

## Abstract

Coilin is a scaffold protein essential for the structure of Cajal bodies, which are nucleolar-associated, nonmembranous organelles that coordinate the assembly of nuclear ribonucleoproteins (RNPs) including spliceosomal snRNPs. To study coilin function in plants, we conducted a genetic suppressor screen using a *coilin (coi1)* mutant in *Arabidopsis thaliana* and performed an immunoprecipitation-mass spectrometry analysis on coilin protein. The *coi1* mutations modify alternative splicing of a *GFP* reporter gene, resulting in a hyper-GFP phenotype in young *coi1* seedlings relative to the intermediate wild-type level. As shown here, this hyper-GFP phenotype is extinguished in older *coi1* seedlings by posttranscriptional gene silencing triggered by siRNAs derived from aberrant splice variants of *GFP* pre-mRNA. In the *coi1* suppressor screen, we identified suppressor mutations in WRAP53, a putative coilin–interacting protein; SMU2, a predicted splicing factor; and ZCH1, an incompletely characterized zinc finger protein. These suppressor mutations return the hyper-GFP fluorescence of young *coi1* seedlings to the intermediate wild-type level. Additionally, *coi1 zch1* mutants display more extensive *GFP* silencing and elevated levels of *GFP* siRNAs, suggesting the involvement of wild-type ZCH1 in siRNA biogenesis or stability. The immunoprecipitation-mass spectrometry analysis reinforced the roles of coilin in pre-mRNA splicing, nucleolar chromatin structure, and rRNA processing. The participation of coilin in these processes, at least some of which incorporate small RNAs, supports the hypothesis that coilin provides a chaperone for small RNA trafficking. Our study demonstrates the usefulness of the *GFP* splicing reporter for investigating alternative splicing, ribosome biogenesis, and siRNA-mediated silencing in the context of coilin function.

## Teaser

Coilin is a scaffold protein essential for the structure of Cajal bodies, which are nucleolar-associated, nonmembranous organelles that coordinate the assembly of nuclear ribonucleoproteins (RNPs) including spliceosomal snRNPs. To study coilin function in plants, Kanno et al. conducted a suppressor screen using a *coilin* mutant in *Arabidopsis thaliana* and performed an IP/MS analysis on coilin protein. Results from their study reinforce the roles of coilin in pre-mRNA splicing, nucleolar chromatin structure, and rRNA processing, with possible involvement in these processes of siRNAs

## Introduction

Coilin is an intriguing protein that is present in most multicellular eukaryotes. Even after decades of research, the full range of coilin's functions and modes of action are not fully understood (Machyna *et al.* 2015). Coilin is best known as an essential structural constituent and prominent marker of Cajal bodies (CBs). CBs are nonmembranous nuclear organelles that are typically situated adjacent to the nucleolus, a second membrane-free nuclear organelle that also contains detectable amounts of coilin (Hebert 2013; Trinkle-Mulcahy and Sleeman 2017). CBs are major sites for the maturation, processing, and quality control of small nuclear RNAs (snRNAs), including those that are present in small nuclear ribonucleoprotein particles (snRNPs) required for pre-mRNA splicing, and small nucleolar RNAs (snoRNAs), which guide processing and maturation of rRNAs and tRNAs in the nucleolus (Machyna *et al.* 2015; Taliansky *et al.* 2023).

Even though coilin appears to be particularly enriched in CBs and is required for their formation and maintenance, a large fraction of coilin protein in animal cells is distributed throughout the nucleoplasm (Lam *et al.* 2002) where its roles are less clear. Through dynamic associations with chromatin, nucleoplasmic coilin in animals is thought to contribute to a number of processes including not only pre-mRNA splicing but also chromatin organization, DNA repair, and telomere maintenance (Machyna *et al.* 2015; Taliansky *et al.* 2023). Whether coilin also performs these multiple functions in plants is not yet clear.

The requirement for coilin to maintain CB structural integrity is illustrated by the ability of coilin mutations to disrupt CBs in animal cells (Liu *et al.* 2009; Carrero *et al.* 2011; Machyna *et al.* 2015) and in *Arabidopsis thaliana* (Arabidopsis) (Collier *et al.* 2006: Kanno *et al.* 2016). Mutations in coilin lead to embryonic lethality or semilethality in vertebrates (Walker *et al.* 2009; Strzelecka *et al.* 2010) whereas they have minimal effects on development in Drosophila (Liu *et al.* 2009) and Arabidopsis (Collier *et al.* 2006; Kanno *et al.* 2016; Abulfaraj *et al.* 2022). In both animals and plants, splicing defects have been observed in coilin-deficient mutants, consistent with a role for coilin in pre-mRNA splicing (Walker *et al.* 2009; Strzelecka *et al.* 2010: Kanno *et al.* 2016; Abulfaraj *et al.* 2022). Expression of stress- and plant immunity-related genes is often altered in coilin mutants in Arabidopsis (Kanno *et al.* 2016; Abulfaraj *et al.* 2022), indicating the importance of coilin for plant protective functions.

The ability of coilin to facilitate CB assembly depends on self-associations and interactions with RNA and other CB proteins (Makarov *et al.* 2013; Machyna *et al.* 2015). Conserved regions of coilin protein in plants and animals include an N-terminal self-association domain; a C-terminal atypical Tudor domain that mediates interactions with several snRNPs (Hebert *et al.* 2001; Xu *et al.* 2005); and a central disordered domain (Makarov *et al.* 2013). Although coilin lacks conventional RNA binding motifs, analysis of the secondary structure of Arabidopsis coilin revealed several degenerate RNA recognition motifs in the N-terminal region and central disordered domain (Makarov *et al.* 2013; Machyna *et al.* 2015). The reported ability of coilin to bind RNAs, particularly small noncoding RNAs such as snRNAs and snoRNAs (Machyna *et al.* 2014), led to the proposal that coilin may serve as a chaperone for the trafficking of nuclear noncoding small RNAs (Machyna *et al.* 2015).

The possible involvement of CBs and coilin in small interfering RNA (siRNA)-induced gene silencing (RNA silencing) has been explored in plants (Li *et al.* 2006; Pontes and Pikaard 2008; Pontes *et al.* 2013), where 21–24-nt siRNAs generated by dicer cleavage of double-stranded RNA (dsRNA) precursors mediate both posttranscriptional and transcriptional gene silencing (PTGS and TGS, respectively). DsRNAs can be produced by RNA-dependent RNA polymerases (RDR) acting on single-stranded RNAs that are perceived by the cell as aberrant in some way. Aberrant RNAs can include “foreign” RNAs derived from viruses and transgenes as well as endogenous RNAs that are untranslatable due to premature termination codons resulting from single nucleotide mutations, mis-splicing, or lack of a poly-A tail. PTGS involves sequence-specific degradation of target RNAs triggered by complementary 21–22-nt siRNAs, which are produced in Arabidopsis by DICER-LIKE4 (DCL4) and DCL2, respectively. TGS is associated with DNA methylation of promoter segments guided by DCL3-dependent 24-nt siRNAs in a process referred to as RNA-directed DNA methylation (RdDM) (Uslu and Wassenegger 2020; El-Sappah *et al.* 2021; Kryovrysanaki *et al* 2022).

In Arabidopsis, factors required for PTGS and TGS, such as DCL enzymes and ARGONAUTE (AGO) silencing effector proteins, have been detected in CBs by immunofluorescence microscopy (Li *et al.* 2006; Pontes and Pikaard 2008; Pontes *et al.* 2013). However, as shown by experiments using an Arabidopsis *ncb* (*no cajal body*) mutant (Collier *et al.* 2006), intact CBs do not seem to be required for siRNA-mediated gene silencing to occur. Neither RdDM nor the production of siRNAs at several tested loci was substantially affected by the loss of CB integrity in *ncb* mutants, although the efficiency of RdDM at these sites may have been somewhat reduced (Li *et al*. 2006).

Our interest in coilin was stimulated by the retrieval of multiple *coilin* (*coi1*) mutants in a forward genetic screen based on an alternatively spliced *GFP* reporter gene in Arabidopsis (Kanno *et al.* 2016, 2020). In this screen, *coi1* mutants were initially identified in young seedlings (shortly after germination) by their hyper-GFP phenotype relative to wild-type seedlings, which display an intermediate level of GFP fluorescence (Kanno *et al.* 2016). Although the basis of the hyper-GFP phenotype in young *coi1* seedlings is not yet completely understood, it can be attributed at least in part to modified splicing of *GFP* pre-mRNA, leading to elevated amounts of the only *GFP* splice variant that can be translated into protein (Kanno *et al.* 2016, 2020).

After publishing the identification of the hyper-GFP *coi1* mutants (Kanno *et al.* 2016), we later observed that in many older *coi1* seedlings (typically at the second to fourth true leaf stage), complete silencing of the *GFP* reporter gene occurs. The silencing pattern, featuring dark red leaves emerging from the shoot apex of a hyper-GFP plantlet, superficially resembled virus recovery, in which asymptomatic leaves appear during the growth of an infected plant. Recovery from virus infection is known to involve PTGS that is provoked by virus-derived siRNAs (Ghoshal and Sanfacon 2015; Uslu and Wassenegger 2020).

Here we report the results of experiments designed to investigate the delayed silencing phenomenon in older *coi1* mutants. We also describe findings from genetic and biochemical analyses that were carried out to identify proteins that may be functionally linked to coilin and potentially illuminate new coilin-dependent pathways.

## Materials and methods

### Plant material and growth conditions

Wild-type and mutant plants used in this study were in the ecotype Col-0 background and cultivated under long-day conditions (22–23°C, 16 hours light, 8 hours dark). Gene names are written in italics and proteins in upright letters. Mutant names are indicated in lowercase letters; wild-type names are in uppercase letters. To test for the occurrence of PTGS, *rdr6-14* and *dcl4-12* alleles were introduced into the wild-type line (*WT T*) and other mutants by intercrossing. For assessments of GFP fluorescence intensity in seedlings, seeds of the desired line were surface sterilized, germinated on solid Murashige and Skoog (MS) medium in plastic Petri dishes, and cultivated in a growth incubator under a 16-hour light/8-hour dark cycle at 24°C. Seedlings were observed daily for fluorescence levels under a Leica fluorescence stereo-microscope over a period of 4–6 weeks.

Seeds of the *WT T* line and coilin suppressor mutants as well as other mutants used in this study are available from the Arabidopsis Biological Research Center (ABRC) under the ABRC seed stock numbers listed in Supplementary Table 1. Accession numbers for data from RNA sequencing, small RNA sequencing, and whole genome DNA resequencing presented in this manuscript are also listed in Supplementary Table 1.

### Nomenclature of GFP phenotypes

Five major GFP phenotypes—as assessed by the strength of GFP fluorescence, which correlated with the level of GFP protein determined by Western blotting—were observed in various genotypes.

**Fig. 1.**
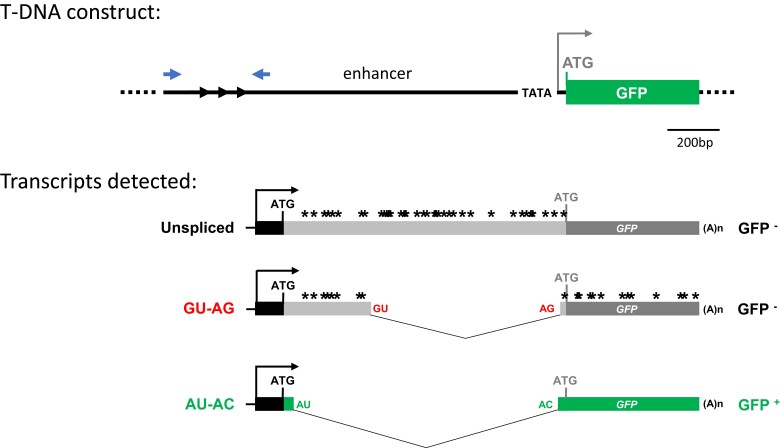
Alternative splicing of *GFP* reporter gene. *T-DNA construct:* One complete copy of the indicated T-DNA construct is integrated into the genome of *WT T* plants. An endogenous pararetrovirus enhancer is upstream of a minimal 35S promoter (TATA) and the anticipated GFP protein CDS (long green bar) The 3 black arrowheads at the 5′ end of the enhancer represent a short tandem repeat, which becomes methylated in the presence of homologous siRNAs (Kanno *et al.* 2008). Opposing blue arrowheads denote the positions of primers used to detect DNA methylation (Supplementary Table 3). Enhancer region, 1277 bp; minimal promoter, 91 bp; expected *GFP* CDS, 720 bp, Total length, 2088bp. *Transcripts detected:* Although transcription was predicted to start downstream of the TATA minimal promoter, this promoter, and the downstream ATG start codon remain unused (gray letters). Instead, transcription initiates at a cryptic promoter upstream in the tandem repeats (black arrow). Three polyadenylated *GFP* transcripts can be detected: an unspliced form (*GFP* pre-mRNA) and 2 spliced variants, which contain canonical (GU-AG) and noncanonical (AU-AC; *GFP* mRNA) splice sites, respectively (Kanno *et al.* 2008). Owing to the presence of stop codons (asterisks) in the unspliced and GU-AG transcripts, only the AU-AC transcript can be translated into GFP protein (green bar). Variations in the proportions of the 3 splice variants by alternative splicing modify the level of GPF fluorescence (Kanno *et al.* 2020). Translation initiates from a new upstream in-frame ATG start codon (black letters), which results in a 27 amino acid N-terminal extension to standard GFP protein (short green bar) (Fu *et al.* 2015).

GFP-intermediate (relative to GFP-weak and hyper-GFP phenotypes) is observed in *WT T* seedlings in the shoot apex, hypocotyl, and root, conforming to the expression pattern of the viral enhancer driving *GFP* expression (Fig. 1; Daxinger *et al.* 2009). The strength of GFP fluorescence in these regions is enhanced in a *rdr6* mutant background.

Hyper-GFP refers to GFP fluorescence that is considerably stronger than that observed in *WT T* plants, and that is usually visible upon germination and maintained thereafter throughout the lifetime of the plant (seen in *coi1* and *cwc16a* mutants in this study).

GFP-weak refers to GFP fluorescence that is visibly lower than that observed in the *WT T* line.

GFP-weak/intermediate refers to microscopically visible GFP fluorescence that is greatly reduced from that of the *coi1* single mutant to approximately the intermediate *WT T* level (seen in *coi* suppressor mutants). Note that the GFP weak and GFP weak/intermediate phenotypes could sometimes be masked by the accumulation of chlorophyll during plant growth. In these cases, it could be difficult to assess GFP silencing occurring at later stages of development.

GFP-negative (neg) refers to no visible GFP fluorescence. Seedlings appear dark red owing to the autofluorescence of chlorophyll at the excitation wavelength for GFP (seen in *gfp* mutants and aerial parts of *coi1* mutants undergoing delayed sense PTGS (S-PTGS).

The GFP phenotypes were visualized using stereo-fluorescence microscopy in several hundred seedlings from multiple generations of each genotype. The GFP phenotypes were consistent with GFP levels determined in selected genotypes by Western blotting. For nearly all genotypes, 100% of the seedlings displayed the GFP fluorescence and silencing phenotypes reported herein. The only exception was delayed S-PTGS in the hyper-GFP *coi1* mutant, which typically occurred in 40–90% of seedlings at the 2nd–4th true leaf stage.

### Forward genetic screen and identification of coilin suppressor mutants

The protocol for forward genetic screens based on an alternatively spliced *GFP* reporter gene in Arabidopsis [*T* line; referred to herein as “wild-type” (*WT*) *T*] (Fig. 1)] has been described previously (Kanno *et al.* 2016, 2018; Kanno, Lin, Fu, Matzke, *et al.* 2017; Kanno, Lin, Fu, Chang, *et al*. 2017). In the present *coilin* suppressor screen, approximately 40,000 Arabidopsis seeds of a homozygous *coi1-8* mutant (also homozygous for the *T* locus) were treated with ethyl methane sulfonate (EMS) and sown on soil (M1 generation). From approximately 20,000 M1 plants that grew to maturity and produced self-fertilized seeds, 52 batches of M2 seeds (the first generation when a recessive mutation can be homozygous) were harvested. M2 seeds were surface sterilized, germinated on solid MS medium in plastic Petri dishes, and cultivated in a growth incubator as described above.

Putative suppressor mutants were identified by examining M2 seedlings under a Leica fluorescence stereo-microscope approximately 7 d after germination for reduced GFP fluorescence relative to the hyper-GFP phenotype of the *coi1-8* single mutant. Around 100 putative suppressor mutants were recovered and grouped into 2 main categories: GFP-weak/intermediate and GFP-neg. Sequencing of the *GFP* reporter gene in the GFP-neg mutants revealed that 5 harbored a previously unidentified loss-of-function mutation in the *GFP* coding sequence (CDS) (Supplementary Table 1; described in Supplementary Fig. 1; Fu *et al.* 2015).

GFP-weak/intermediate mutants that contained a wild-type *GFP* nucleotide sequence were further tested to confirm: (1) stable meiotic inheritance of the GFP-weak/intermediate phenotype; (2) the presence of the expected *coi1-8* mutation in the endogenous *coi1* gene; and (3) the absence of any second-site intragenic suppressor mutations induced by the EMS treatment into the *coi1-8* gene sequence. GFP-weak mutants that passed these additional tests were then subjected to next-generation mapping (NGM) (James *et al*. 2013; Kanno *et al.* 2020) and/or candidate gene sequencing to identify the respective causative suppressor mutations. Mutations were confirmed by the identification of multiple alleles and/or complementation analyses. All mutations reported here are recessive.

### Western blotting using a GFP antibody

Western blotting to determine the relative levels of GFP protein level in the *coi1 wrap53, coi1 smu2*, and *coi1 zch1* double mutants compared to the *coi1* single mutant and *WT T* line was performed as described in a previous publication (Fu *et al.* 2015). For loading control, a duplicate gel containing the same samples was run and stained with Coomassie Brilliant blue.

### RNA and small RNA sequencing (RNAseq and sRNAseq)

Total RNA was prepared using a Plant Total RNA Miniprep kit (GMbiolab, Taiwan) from 2-wk-old seedlings of the *WT T* line and the *coi1 wrap53, coi1 smu2*, and *coi1 zch1* double mutants cultivated on solid MS medium as described above. RNA concentrations were assessed by NanoDrop (ND-1000 spectrophotometer). Library preparation, RNAseq and sRNAseq were performed (biological triplicates for each sample) as described previously by an in-house Genomic Technology Core facility (Kanno *et al.* 2016). Whole genome resequencing of the *coi1 wrap53, coi1 smu2*, and *coi1 zch1* double mutants was carried out to identify any remaining EMS-induced second-site mutations that alter splice sites. These mutations were then excluded from the analysis of alternative splicing (AS).

### Analysis of RNAseq and sRNAseq data

Pair-ended RNAseq reads were mapped to the Arabidopsis TAIR10 genome in two steps. The first step was to map reads to a combined transcriptome database of Araport11 (Cheng *et al.* 2017) and Atrtd2 (Zhang *et al.* 2017) using Bowtie2 (Langmead and Salzberg 2012), where only ungapped alignments of read pairs been were mapped to the same transcripts were accepted. Coordinates of these transcriptome-mapping alignments were transferred to the genome. The second step was to map the rest reads to the TAIR10 genome using BLAT (Kent 2002) directly.

To gain alignments of small RNA reads to the GFP-containing vector pAJM-EPRV (GenBank accession: HE582394.1) as precise as possible, the following prioritized approach was adopted: (1) reads were mapped to the canonical GFP transcript, only perfect matches were accepted, (2) for splicing junctions supported by RNAseq data, reads were mapped to junction-spanning fragments and only perfect matches were accepted, (3) repeat above 2 steps by allowing 1 mismatch, and (4) rest reads were mapped to pAJM-EPRV using BLAT with a set of fine scanning parameters (–minMatch = 1 –minScore = 14 –tileSize = 9) and allowing one mismatch. For comparing abundances of GFP siRNAs of different lengths between samples, corresponding read counts were normalized into read count per million reads, as well as read coverage per million reads along the GFP region in pAJM-EPRV.

AS detection for intron-retention (IR), exon-skipping (ES), and alternative donor/acceptor (altDA) was done using a similar method as described in our previous publications (Kanno, Lin, Fu, Chang, *et al*. 2017; Kanno, Lin, Fu, Matzke, *et al.* 2017; Kanno *et al.* 2018). For each kind of AS event, read-based counts that are supporting the AS event from the control and treatment samples were considered as signals, whereas read-based counts that are related but not supporting the event were considered as backgrounds. In practice, intron read depths, counts of splicing read skipping exon(s), and counts of reads exactly supporting one donor–acceptor pair were taken as the signals for IR, ES, and altDA events, respectively. For backgrounds, read depths of neighboring exons, counts of splicing reads involving one skipped exon, and counts of reads not supporting the donor–acceptor pair were taken for IR, ES, and altDA events, respectively. Signals from the control and treatment samples were then compared to the backgrounds using χ^2^ test for goodness-of-fit for discovering the differential preference of AS events in the 2 samples. of-fit for discovering differential preference of AS events in the 2 samples. In this study, an AS event was considered significant if its *P*-value is below 0.05. Note that these AS comparisons on RNAseq data were made by accumulating numbers from three biological replicates, i.e. numbers of merged samples. This is because read numbers on AS events are usually smaller than those for differentially expressed gene discovery. Doing so would give us a higher statistical power when applying the χ^2^-test for goodness-of-fit than separate biological replicates. Also, note that *P*-values here were not corrected because our aim is to discover general AS differences among samples under the same comparison standard.

### Protein immunoprecipitation of epitope-tagged coilin proteins

Total proteins were extracted from 2-wk-old seedlings of coilin-mRed, FLAG-coilin, and coilin-FLAG transgenic plants. Protein extraction and immunoprecipitation were carried out according to a published protocol (Huang *et al.* 2016). Briefly, 5 grams of seedlings were ground to a powder in liquid nitrogen. After grinding, 14 ml of SPII + buffer [100 mM Na-phosphate, pH 8.0, 150 mM NaCl, 5 mM EDTA, 5 mM EGTA, 0.1% NP-40, 1 mM PMSF, 5μM MG132, 1× Phosphatase inhibitor cocktail 2 (Sigma-Aldrich), 1× Phosphatase cocktail 3 (Sigma-Aldrich), and a protease inhibitor (cOmplet EDTA-free Protease Inhibitor Cocktail, Roche) were added to the ground sample. The solution was sonicated followed by centrifugation (2 times at 20,000g for 10 min at 4°C) to remove insoluble cell debris. The supernatant constituted the protein extract that was used for immunoprecipitation.

One hundred microliters of Anti-FLAG M2 Magnetic Beads (Millipore) or RFP-trap magnetic beads (ChromoTek) were washed 3 times with 1 ml of SII buffer and then added to the protein extract. The whole solution was incubated at 4°C for 1 hour with 20 rpm agitation. Following this step, the beads were collected using a magnetic stand and washed 3 times with 10 ml of SPII buffer. The bound proteins were eluted by adding elution buffer [100 mM Na-phosphate, pH 8.0, 150 mM NaCl, 0.05% Triton X-100, 500μg/ml 3× FLAG peptide (Millipore)] for FLAG- immunoprecipitation (IP) samples or, for mRed-IP sample, according to the manufacturer's instruction, incubating the beads with 0.2 M glycine pH 2.5 solution followed by adding 1 M Tris base pH 10.4 for neutralization.

### LC-MS/MS analysis and data processing

Digested peptides were injected into a Thermo Scientific Ultimate 3000 coupled with a Thermo Scientific Q-Exactive Mass Spectrometer. The samples were run in a linear 90 min gradient at a flow rate of 300 nL/min. The raw files were searched against the Araport_11 database using Proteome Discoverer (version 2.2). The peptide precursor mass tolerance was set at 10 ppm, and MS/MS tolerance was set at 0.02 Da. The false discovery rate (FDR) at protein and peptide levels was set at 1%. The variable modification was set as oxidation on Methionine resides, and the cysteine carbamidomethylation was set as a static modification.

## Results and discussion

### Investigation of delayed GFP silencing in older coi1 seedlings

#### Characteristics of delayed *GFP* silencing in *coi1* single mutants

**Fig. 2.**
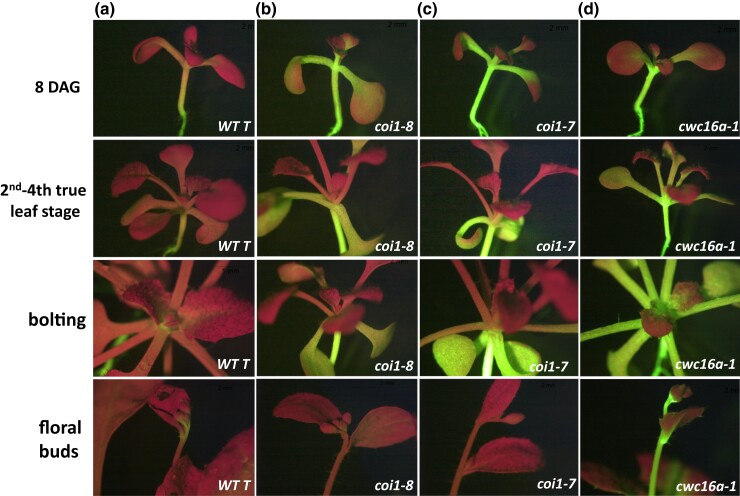
Delayed *GFP* silencing in *coi1* single mutants. a–c) Row 1: “Young” seedlings (8 DAG) of *coi1* single mutants (2 alleles: *coi1-7* and *coi1-8*; Supplementary Fig. 5) display hyper-GFP phenotypes relative to the *WT T* line, which has an intermediate level of GFP fluorescence. Rows 2, 3: In “older” *coi1* seedlings (2nd–4th true leaf stage), abruptly delayed silencing, typified by dark red leaves emerging from a hyper-GFP stem, can occur. Similar delayed and abrupt silencing was also observed with a *GUS* transgene in Arabidopsis line L1 (Elmayan *et al* 1998) Row 4: Delayed silencing persists into the floral stage. A) all rows: Most *WT T* plants also continue to show faint but visible accumulation of GFP fluorescence into the floral stage. A, all rows). D, all rows): The hyper-GFP *cwc16a* mutant (Kanno *et al.* 2017a, 2020) does not undergo delayed silencing but remains hyper-GFP during the entire lifetime of the plant.

The *coi1* mutants were identified in a previous forward genetic screen based on a wild-type” *T* line (*WT T*) containing an alternatively spliced *GFP* reporter gene in Arabidopsis (Fig. 1) (Kanno *et al.* 2016, 2020). *WT T* seedlings display an intermediate level of GFP fluorescence relative to 2 possible extremes: GFP-weak and hyper-GFP (Kanno *et al.* 2020). The *coi1* mutants could be distinguished from wild-type in young seedlings by their hyper-GFP phenotype [Fig. 2, a–c; 8 days after germination (DAG)] (Kanno *et al.* 2016).

After publication of these results (Kanno *et al.* 2016), we subsequently found that later in development—typically the second to fourth true leaf stage (referred to here as “older” seedlings, possibly coincident with the juvenile-to-adult transition; Bui *et al.* 2020)—40–90% of *coi1* seedlings spontaneously undergoes a *GFP* silencing phenomenon characterized by the abrupt emergence of dark red leaves, indicative of *GFP* gene silencing, from a hyper-GFP shoot. This silencing does not infiltrate below the point of initiation to lower parts of the seedling, which stay hyper-GFP (Fig. 2, b and c, older seedlings). Once triggered, delayed *GFP* silencing in older *coi1* seedlings persists in adult plants through the reproductive stage (Fig. 2, b and c, floral buds).

#### *Coi1* mutants and *WT T* seedlings contain siRNAs likely derived from aberrant but not translatable *GPF* transcripts

**Fig. 3.**
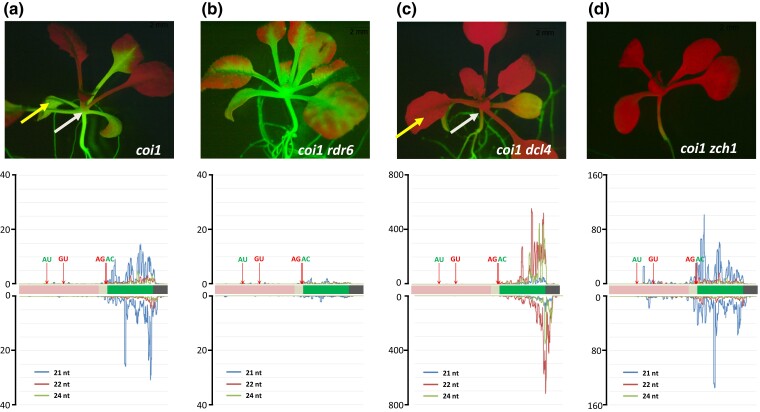
Delayed *GFP* silencing in *coi1* mutants is due to siRNA-mediated s-PTGS. a) *Top*: delayed S-PTGS in *coi1* single mutant. Hyper-GFP fluorescence is extinguished at the 2nd–4th true leaf stage and does not infiltrate below the site of initiation (white arrow). *Bottom*: *GFP* siRNAs, predominantly 21-nt in length (blue lines), are distributed throughout the *GFP* CDS (green bar) and more sparsely, in the upstream region flanked by AU and GU splice sites. b) *Top*: *coi1 rdr6* double mutants remain hyper-GFP throughout the plant's lifetime and *GFP* siRNAs are essentially eliminated (*Bottom*). c) *Top*: *coi1 dcl4* double mutants display strong *GFP* silencing in aerial parts of a seedling including, the primary true leaves (yellow arrow), which remain mostly hyper-GFP in the single *coi1* mutant a, yellow arrow). Unlike the more spatially restricted delayed S-PTGS in *coi1* single mutants, silencing in *coi1 dcl4* double mutants extends into the shoot apex and the top of the hypocotyl (compare a and c, white arrows), coincident with a large increase of 22- nt *GFP* siRNAs (orange lines, *Bottom*). The more extensive *GFP* silencing observed in a *coi1 dcl4* background potentially masks any abrupt *GFP* silencing that could occur later in older *coi dcl4* double mutants. d) *coi1 zch1* double mutants exhibit a pervasive silencing phenotype similar to that observed in *coi1 dcl4* but the size class of the predominant *GFP* siRNA differs: 22 nt in *coi1 dcl4* vs 21 nt in *coi1 zch1*. Seedlings were photographed at approximately 21 DAG. Length and distribution of *GFP* siRNAs along the *GFP* CDS and upstream region are indicated. Y-axes show read coverage per million reads (scale differs in each graph). Quantitative comparative differences are shown in Supplementary Fig. 3. AU-AC splice sites are shown in green letters, indicating that the resultant RNA is translated into GFP protein. GU-AG splice sites are shown in red letters indicating that the resultant RNA is untranslatable (see Fig. 1).

**Fig. 4.**
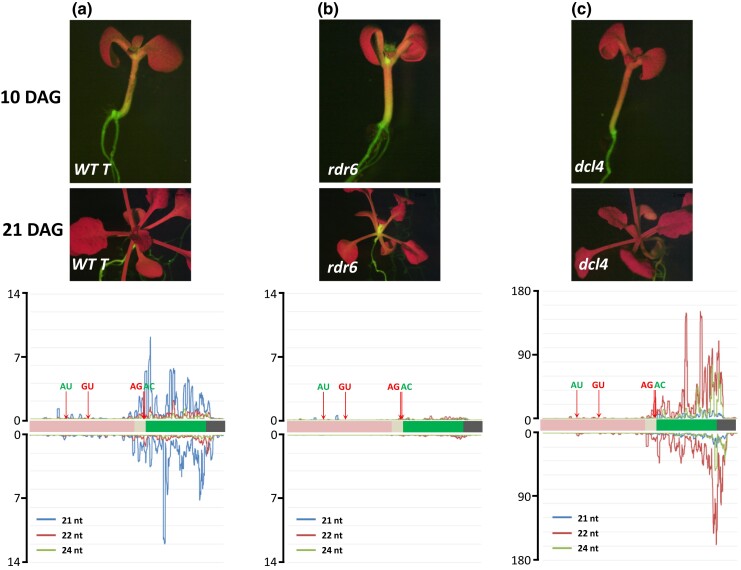
Pre-existing intermediate PTGS of *GFP* in *WT t* line. a) *Top*: The *WT T* line shows intermediate GFP fluorescence that is highest in the shoot apex, hypocotyl, and portions of the root (Daxinger *et al.* 2009). *Bottom*: *GFP* siRNAs in *WT T* are predominantly 21-nt in length (blue lines) and likely originate from the spliced, untranslatable GU-AG transcript (Supplementary Fig. 2). b) *Top*: an *rdr6* mutation introduced into the *WT T* line increases GFP fluorescence in the shoot apex and hypocotyl and eliminates nearly all *GFP* siRNAs (*Bottom*). c) *Top*: a *dcl4* mutation results in diminished GFP fluorescence that gradually spreads into the shoot apex and hypocotyl. *Bottom*: 22-nt siRNAs (red lines) are substantially increased in a *dcl4* mutant background while 21-nt siRNAs are greatly reduced. Seedlings were photographed at 10 and 21 DAG. Length and distribution of *GFP* siRNAs along the *GFP* CDS and upstream region are indicated. Y-axes show read coverage per million reads (scale differs in each graph). Quantitative comparative differences are shown in Supplementary Fig. 3. AU-AC splice sites are shown in green letters, indicating that the resultant RNA is translated into GFP protein. GU-AG splice sites are shown in red letters indicating that the resultant RNA is untranslatable (see Fig. 1).

To understand the delayed *GFP* silencing phenomenon in older *coi1* mutants, we tested whether it was associated with *GFP* small RNAs. We sequenced small RNAs in *coi1* mutant seedlings and as a control, in *WT T* seedlings, which we assumed would lack *GFP* small RNAs because there was no prior evidence for PTGS in this line. *GFP* small RNAs of both sense and antisense orientations were indeed detected, not only in the *coi1* single mutant (Fig. 3a) but also, unexpectedly, in *WT T* plants displaying intermediate levels of GFP fluorescence (Fig. 4a).

In both the *coi1* mutant (Fig. 3a) and *WT T* plants (Fig. 4a), the *GFP* small RNAs were predominantly 21-nt in length, with lesser amounts of 22 and 24-nt small RNAs. Although the siRNAs were primarily derived from the *GFP* protein CDS, much lower amounts of mainly 21-nt *GFP* small RNAs could be reproducibly detected upstream of the *GFP* CDS, particularly in an exon sequence present in the spliced GU-AG and unspliced transcripts but not in the translatable AC-AU variant (Supplementary Fig. 2, blue box). Neither of the former two transcripts is translatable owing to the presence of numerous stop codons (Fig. 1), which could potentially channel them into a pathway of siRNA production and PTGS (Liu and Chen 2016). However, the relative paucity of siRNAs from the unique central region of the unspliced transcript (Supplementary Fig. 2, red dotted box) suggests that the spliced GU-AG transcript is the primary source of upstream *GFP* small RNAs. The substantial quantitative reduction in *GFP* small RNAs in the upstream sequence compared to the *GFP* CDS may reflect the decreased activity of RDR6 as it progresses 3′ to 5′ along the substrate RNA (Moissiard *et al.* 2007; Supplementary Fig. 2).

**Fig. 5.**
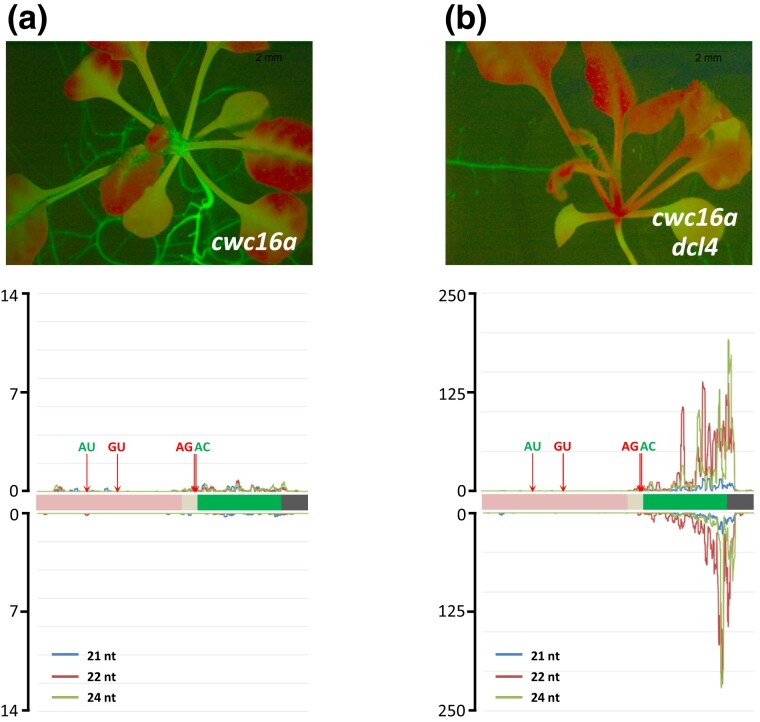
Delayed *GFP* silencing does not occur in a single *cwc16a* mutant, which lacks *GFP* siRNAs. a) *Top*: *cwc16* mutants do not undergo delayed silencing, remaining hyper-GFP throughout the plant's lifetime, and (*Bottom*) contain negligible amounts of *GFP* siRNAs. b) *Top*: *cwc16a dcl4* double mutants undergo silencing that extends into the shoot apex and top of hypocotyl, and they produce large quantities of 22-nt *GFP* siRNAs (*Bottom*) owing to strong DCL2 activity (presumably acting on dsRNA synthesized from minute amounts of aberrant *GFP* transcripts) in the absence of DCL4. Seedlings were photographed at approximately 21 DAG. Length and distribution of *GFP* siRNAs along the *GFP* CDS and upstream region are indicated. Y-axes show read coverage per million reads (scale differs in each graph). Quantitative comparative differences are shown in Supplementary Fig. 3. AU-AC splice sites are shown in green letters, indicating that the resultant RNA is translated into GFP protein. GU-AG splice sites are shown in red letters indicating that the resultant RNA is untranslatable (see Fig. 1).

Additional evidence that the abundant small RNAs originating from the *GFP* CDS are also likely to originate from aberrant *GFP* transcripts and not the translatable AU-AC RNA is provided by the *cwc16a* mutant (Fig. 2d). This mutant was identified by its hyper-GFP phenotype in the same splicing screen as the *coi1* mutant (Kanno, Lin, Fu, Matzke, *et al.* 2017; Kanno *et al.* 2020). The *cwc16a* mutant, which is defective in a putative step 2 splicing factor, produces almost exclusively the spliced, translatable AU-AC transcript (Kanno, Lin, Fu, Matzke, *et al.* 2017), and—in agreement with the hypothesis that *GFP* small RNAs are not derived from this transcript—the *cwc16a* mutant is essentially devoid of *GFP* small RNAs from both the *GFP* CDS and upstream regions (Fig. 5a; Supplementary Fig. 3). Consistent with the lack of *GFP* siRNAs in the *cwc16a* mutant, delayed *GFP* silencing has not yet been observed in hundreds of *cwc16a* seedlings examined, which remain hyper-GFP during the lifetime of the plant (Fig. 2d).

The collective findings indicate that the translatable AU-AC transcript is largely excluded from the small RNA biogenesis pathway. Thus, among the three *GFP* splice variants detected in the *GFP* splicing reporter system (Fig. 1), the untranslatable GU-AG transcript, and perhaps to a lesser extent, the unspliced transcript, represent the most credible sources of all *GFP* small RNAs (Supplementary Fig. 2).

#### *GFP* small RNAs are bona fide siRNAs that trigger delayed *GFP* silencing in *coi1* mutants

To determine whether the *GFP* small RNAs are authentic siRNAs capable of triggering PTGS-of the *GFP* reporter gene, we crossed a *coi1* mutant and *WT T* plants with mutants defective in the PTGS factors RNA-DEPENDENT RNA POLYMERASE 6 (RDR6), which copies aberrant RNAs to produce dsRNAs, and DCL4, which cleaves dsRNAs into 21-nt siRNAs (Lopez-Gomollon and Baulcombe 2022). As described below, these experiments not only implicated classical sense (S)-PTGS in delayed *GFP* silencing in older *coi1* mutants, but they also revealed a previously unsuspected, moderate level of S-PTGS in *WT T* seedlings.

Relative to the single *coi1* mutant (Fig. 3a), *coi1 rdr6* seedlings displayed increased GFP fluorescence and the loss of virtually all size classes and orientations of *GFP* small RNAs (Fig. 3b). Notably, delayed *GFP* silencing similar to that observed in older *coi1* single mutants (Figs. 2, b and c, 3a) was never observed in *coi1 rdr6* seedlings, which remained hyper-GFP throughout growth and development (Fig. 3b).

Conversely, *coi1 dcl4* seedlings showed substantially reduced *GFP* fluorescence compared to the *coi1* single mutant (Fig. 3c). However, unlike delayed silencing in older *coi1* seedlings, which does not infiltrate below the site of initiation (Figs. 2, b and c, 3a), diminished *GPF* fluorescence in *coi1 dcl4* seedlings occurred earlier and extended into the shoot apex and the top part of hypocotyl (compare Fig. 3, a and c, white arrows). The more extensive *GFP* silencing in *coi1 dcl4* seedings was associated with a substantial reduction in 21-nt *GFP* siRNAs and a massive increase in 22-nt *GFP* siRNAs (Fig. 3c; Supplementary Fig. 3c). Previous work has shown that a DCL4 deficiency results in elevated DCL2 activity on double-stranded RNA substrates, resulting in increased production of 22-nt siRNAs that can enhance silencing (Mlotshwa *et al.* 2008; Chen *et al.* 2010; Parent *et al.* 2015; Taochy *et al.* 2017).

Similar changes were seen when *rdr6* and *dcl4* mutations were introduced into the *WT T* line: *rdr6* mutations enhanced GFP fluorescence and eliminated *GFP* siRNAs; *dcl4* mutations reduced GFP fluorescence and increased the accumulation of 22-nt siRNAs (Fig. 4, b and c, respectively).

Greatly elevated levels of 22-nt *GFP* siRNAs and extensive *GFP* silencing were also observed in *cwc16a dcl4* double mutants (Fig. 5c). This finding indicates that the *cwc16a* single mutant, which normally does not produce *GFP* siRNAs or exhibit S-PTGS, is nevertheless capable of undergoing S-PTGS provided sufficient numbers of siRNAs, such as the elevated level of 22-nt siRNAs in a *cwc16a dcl4* double mutant, are available.

#### Summary and limitations of our analysis of delayed S-PTGS in *coi1* mutants and *GFP* siRNAs

The results of experiments with the *rdr6* and *dcl4* mutants demonstrated that the abrupt, nonspreading delayed silencing phenotype observed in older *coi1* single mutants is due to canonical S-PTGS triggered by 21–22-nt *GFP* siRNAs, which likely originate largely from the aberrant GU-AG *GFP* splice variant. What remains unclear is why delayed S-PTGS is provoked suddenly at a relatively late period of seedling development and not earlier in young *coi1* seedlings. Despite the presence of detectable amounts of 21–22-nt *GFP* siRNAs in young (2-wk-old) *coi1* seedlings, they consistently display a hyper-GFP phenotype before the sudden appearance of delayed S-PTGS in older *coi1* seedlings (Fig. 2).

One possibility is that the threshold number of siRNAs required to effectively induce S-PTGS is only surpassed in older *coi1* seedlings, perhaps due to enhanced transcription of aberrant *GFP* splice variants at that time. It is currently difficult to evaluate this hypothesis because RNA-seq data are currently available only from 2-wk-old seedlings. The proportions of the 21–22-nt *GFP* siRNAs (Supplementary Fig. 3b) and the 3 *GFP* splice variants (Supplementary Fig. 4) differ to some extent between the *WT T* line and the *coi1* mutant at 2 wks. However, the effects of these differences in *GFP* siRNAs are difficult to assess, since the threshold level of siRNAs needed to induce S-PTGS is not known. Moreover, the differences in *GFP* splice variants affect primarily the translatable AU-AC splice variant (Supplementary Fig. 4), which is not a major source of *GFP* siRNAs (Supplementary Fig. 2). Whether these quantitative variations in *GFP* siRNAs and splice variants observed in 2-wk-old seedlings are elevated during development to eventually trigger the sharp onset of delayed S-PTGS in older *coi1* seedlings is not yet known.

#### Implications of pre-existing *GFP* siRNAs in the *WT T* line

Small RNA sequencing and experiments with *rdr6* and *dcl4* mutants also revealed that the *WT T* line contains an endogenous and formerly unsuspected population of 21–24-nt *GFP* siRNAs that trigger a modest level of continuous S-PTGS, resulting in intermediate *GFP* expression. The intermediate level of *GFP* expression in the *WT T* line can be shifted to either a GFP-weaker or a stronger GFP phenotype by mutations in factors involved in PTGS (DCL4 and RDR6, respectively; this study), which change the proportions and abundance of different size classes of *GFP* siRNAs (Figs. 3 and 4), or by mutations in specific splicing-related factors (such as COI1, CWC16A PRP8A, PRP18A), which alter the proportions of the three *GFP* splice variants (Sasaki *et al.* 2015; Kanno *et al.* 2016, 2018, 2020; Kanno, Lin, Fu, Matzke, *et al.* 2017; Kanno, Lin, Fu, Chang, *et al*. 2017).

Given the new finding that the *WT T* line contains an endogenous pool of *GFP* siRNAs that can provoke a moderate degree of S-PTGS, it is conceivable that some of the mutants we identified in the previous screen for splicing factors (Kanno *et al.* 2020) could also modulate S-PTGS. Indeed, a recent study reported that PRP39 (identified in our splicing screen by its hyper-GFP phenotype; Kanno, Lin, Fu, Chang, *et al*. 2017b), has distinct roles in not only splicing but also in S-PTGS (Bazin *et al.* 2023).

**Fig. 6.**
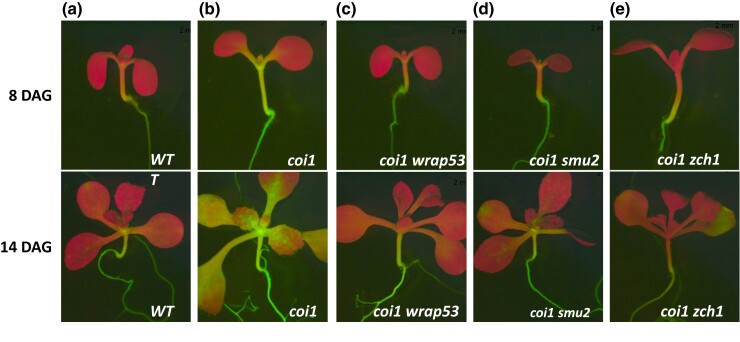
GFP phenotypes in coilin suppressor screen mutants. a, b) Intermediate GFP fluorescence in the *WT T* line compared to the hyper-GFP phenotype in a nonsilenced *coi1* single mutant, which was derived from the *WT T* line (Kanno *et al.* 2016), at 8 and 14 DAG. The *coi1* suppressor screen identified mutations that attenuate the hyper-GFP phenotype of the single *coi1* mutant. c–e) Reduced *GFP* expression in the *coi1 wrap53, coi1 smu2*, and *coi1 zch1* double mutants at 8 and 14 DAG. The *coi1 zch1* double mutant seedlings e) show shortly after germination a strong reduction of GFP fluorescence that gradually extends into the shoot apex and hypocotyl. This resembles the silencing pattern observed when *dcl4* mutations are introduced into the *WT T* line, although the size class of siRNA that accumulates differs [21-nt in *coi1 zch1* vs 22-nt in *dcl4* (Fig. 3, c and d)]. Silencing observed in *coi1 zch1* double mutants is also distinct from the delayed silencing phenotype in *coi1* single mutants, which occurs later during seedling growth and does not spread below the site of initiation (Fig. 3a). Moreover, the premature and extended silencing occurs in 100% of *coi1 zch1* seedlings whereas delayed silencing occurs in only 40–90% of *coi1* seedlings. Despite the differences in the frequency, timing, and pattern of *coi1 zch1* silencing vs *coi1* delayed silencing, both are associated with 21-nt *GFP* siRNAs derived from the *GFP* CDS (Fig. 3, a and d).

Note that the relationship between moderate S-PTGS in *WT T* and delayed S-PTGS in *coi1* mutants is not entirely clear. Our data indicate that the two types of S-PTGS are phenotypically distinct with respect to the timing of silencing, the strength and frequency of silencing, and the *GFP* expression and silencing pattern (Table 1). In *WT T* seedlings, moderate S-PTGS occurs continuously during plant growth and development and affects 100% of seedlings. The pattern of the resulting intermediate *GFP* expression reflects the activity of the viral enhancer upstream of the *GFP* protein coding region (shoot apex, hypocotyl, and root; Fig. 2a; Table 1). This pattern is maintained when *GFP* expression is increased (that is, S-PTGS is suppressed) in an *rdr6* mutant (Fig. 4, a and b; Table 1). By contrast, the *coi1* mutant shows a pervasive pattern of hyper-GFP fluorescence that is visible upon germination (Kanno *et al.* 2016) and maintained during the lifetime of the plant, provided delayed S-PTGS does not occur (Figs. 4b, 6b). When delayed S-PTGS does occur in 40–90% of older *coi1* seedlings, complete and nonspreading *GFP* silencing is observed in all aerial parts of the plant above the point of initiation (Fig. 2b; Table 1). We think this description of the 2 types of S-PTGS in the *GFP* splicing reporter system best fits our data. However, until this system is fully understood, we cannot completely rule out possible alternative interpretations.

**Table 1.** Summary of genotypes, GFP phenotypes, *GFP* siRNAs, and silencing patterns in the mutants described in this study.

Genotype	GFP phenotype (fluorescence microscopy)	GFP silencing (fluorescence microscopy)	GFP small RNAs	References
*WT T*	GFP-intermediate in shoot apex, hypocotyl, and root (Fig. 2a)	Moderate level of S-PTGS that does not completely silence GFP (Figs. 2a, 4a)	21–24 nt; predominantly DCL4-dependent 21-nt siRNAs (Fig. 4a)	Kanno *et al*. (2016) Daxinger *et al*. (2009)
*coi1*	Hyper-GFP throughout seedling visible upon germination (Fig. 2, b and c, 8 DAG); can later undergo delayed S-PTGS	Delayed S-PTGS at 2nd–4th true leaf stage (Fig. 2, b and c, older seedlings and before bolting; Fig. 3a)	21–24 nt; Predominantly DCL4-dependent 21-nt siRNAs (Fig. 3a)	Kanno *et al*. (2016); this study
*cwc16a*	Hyper-GFP at all stages of development (Fig. 2d)	No GFP silencing (Fig. 2d)	Negligible amounts (Fig. 5a)	Kanno, Lin, Fu, Matzke, *et al.* (2017), Kanno, Lin, Fu, Chang, *et al*. (2017); this study
*rdr6*	Enhanced GFP in shoot apex and root relative to WT T (compare Fig. 4, a and b)	No GFP silencing (Fig. 4b)	All GFP siRNAs were eliminated (Fig. 4b)	This study
*rdr6 coi1*	Hyper-GFP throughout the plant (Fig. 3b)	No GFP silencing, even in “older” seedlings (Fig. 3b)	All GFP siRNAs were eliminated (Fig. 3b)	This study
*dcl4*	Weak but visible GFP in the shoot apex and root (Fig. 4c, 10 DAG)	GFP silencing in most of the seedling (Fig. 4c, 21 DAG)	21–24 nt; Predominantly DCL2-dependent 22-nt siRNAs (Fig. 4c)	This study
*dcl4 coi1*	GFP reduced	GFP silencing in most of the seedling (Fig. 3c)	DCL2-dependent 22 nt siRNAs predominate (Fig. 3c)	This study
*dcl4 cwc16a*	GFP reduced	GFP silencing in most of the seedling (Fig. 5b)	DCL2-dependent 22 nt siRNAs predominate (Fig. 5b)	This study
*wrap53 coi1*	Weak-intermediate (WT T pattern) (Fig. 6)	-	21 nt (DCL4)	This study
*smu2 coi1*	Weak-intermediate (WT T pattern) (Fig. 6)	-	21 nt (DCL4)	This study
*zch1 coi1*	Weak-intermediate (WT T pattern) 8 DAG (Fig. 6)	Silencing later intensifies and spreads into root (14 DAG) (Figs. 3d, 6)	21 nt (DCL4)	This study

GFP-intermediate refers to the level of GFP fluorescence in *WT T* relative to the GFP-weak and hyper-GFP phenotypes (observed in various mutants). GFP fluorescence in *WT T* is visible in the shoot apex, hypocotyl, and root, reflecting the expression pattern of the viral enhancer upstream of the *GFP* CDS (Fig. 1). Fluorescence in *WT T* can be either enhanced or reduced, respectively, in *rdr6* and *dcl4* mutant backgrounds (this study).

Hyper-GFP: GFP fluorescence observed in some splicing mutants of the *WT T* line (e.g. co*i1, cwc16a*; Kanno *et al.* 2020) that is considerably stronger than the intermediate GFP levels in *WT T*. The hyper-GFP phenotype is visible upon germination and throughout most of the seedling during growth and development.

GFP-weak: GFP fluorescence that is visibly lower than that observed in the *WT T* line. Observed in some splicing mutants (Kanno *et al.* 2020).

GFP-weak/intermediate: GFP fluorescence that is greatly reduced from that of the *coi1* single mutant to approximately the intermediate *WT T* level) (*coi* suppressor mutants; this study).

GFP-negative (neg): whole seedlings (or parts of seedlings) exhibit no visible GFP fluorescence and appear dark red owing to the autofluorescence of chlorophyll at the excitation wavelength for GFP. Observed with *gfp* loss-of-function mutations (Fu *et al.* 2015; this study) and emerging leaves of hyper-GFP *coi1* mutants undergoing delayed S-PTGS (this study).

Delayed S-PTGS refers to abrupt and complete *GFP* silencing observed at the 2nd−4th true leaf stage in 40–90% of *coi1* seedlings. Silencing does not spread below the site of initiation and is maintained throughout the lifetime of the plant.

#### Future perspective

An analysis of the time course of *GFP* pre-mRNA synthesis and alternative splicing patterns, as well as the accumulation of *GFP* siRNAs and GFP protein, starting with young *coi1* and *WT T* seedlings and extending into later stages of development, would help to address remaining uncertainties about delayed S-PTGS in older *coi1* seedlings. Investigation of these questions in our well-defined transgene system is likely to reveal more general principles relevant to developmentally associated changes in alternative splicing patterns that can potentially affect siRNA production and expression of endogenous genes.

### Identifying proteins that interact with coilin

To learn more about components of coilin-dependent pathways in plants and how coilin mutations foster the hyper-GFP phenotype in young *coi1* seedlings, we performed a genetic suppressor screen using a *coi1* mutant and conducted an IP-mass spectrometry (MS) analysis using epitope-tagged coilin proteins.

#### Genetic suppressor screen

EMS mutagenesis and initial tests for putative suppressor mutants were performed as described in the Methods section using seeds of the hyper-GFP *coi1-8* mutant (Supplementary Fig. 5). Of the GFP-weak/intermediate suppressor mutants that passed the initial tests and were subsequently carried on for further analysis (Methods), 5 contained a mutation in a gene retrieved in the former forward screen using the *WT T* line to identify splicing factors: *prp4k*, *sac3a*, *cbp80*, *prp8a*, and *prp18a* (Supplementary Table 1; Kanno *et al.* 2020). The finding of these 5 genes in both the splicing screen and the *coi1* suppressor screen suggests that the involvement of the respective factors in *GFP* pre-mRNA splicing is independent of coilin function. However, 5 GFP-weak suppressor mutants contained mutations in novel factors that were identified only in the present *coi1-8* suppressor screen: *wrap53, smu2, zch1*, *mad1*, and *sde2-like* (Supplementary Table 1). The failure to identify these 5 factors in the former splicing screen suggests their detection in our system is related in some way to the coilin deficiency in the *coi1* mutant. Here we discuss *wrap53*, *smu2*, and *zch1*. In a *coi1* mutant background, these mutations reduce the intensity of GFP fluorescence and the levels of GFP protein to approximately the intermediate state of the *WT T* line (Fig. 6a–e, 8 DAG; Supplementary Fig. 6). In addition, *coi1 zch1* mutants eventually display enhanced *GFP* silencing that extends into the shoot apex and top of the hypocotyl (Fig. 6e).

#### *WRAP53* (AT4g21520)

**Fig. 7.**
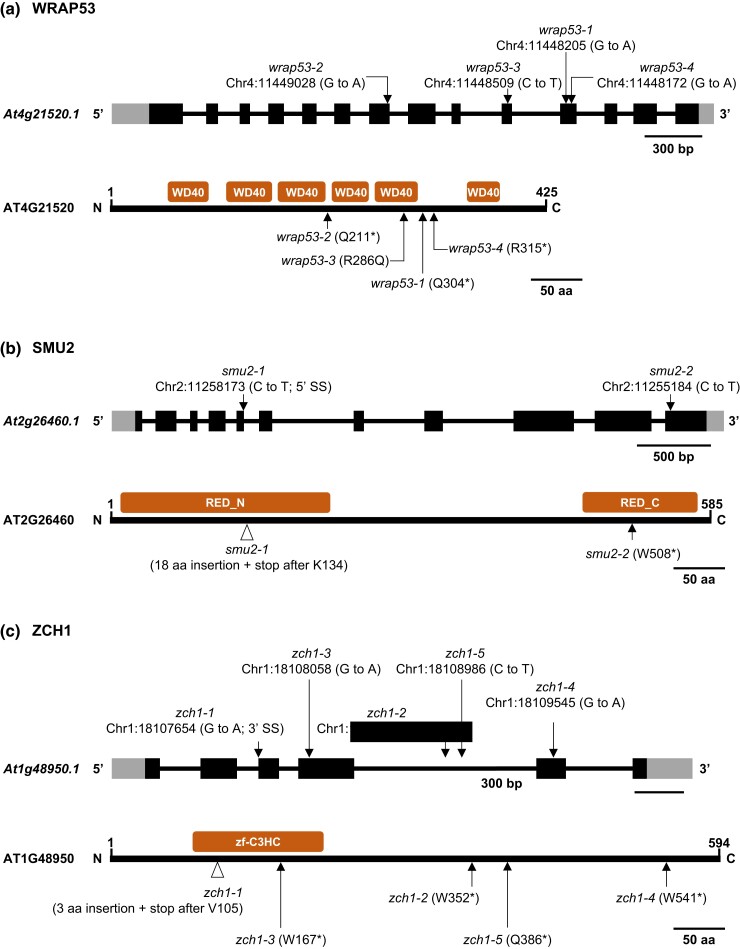
*G*ene structures, protein domains, and positions of mutations in *Arabidopsis thaliana WRAP53, SMU2*, and *ZCH1*. Intron–exon structures of the indicated genes and nucleotide sequence changes are shown at the top of each section; changes in amino acid sequences are at the bottom (asterisks indicate premature termination codons). Recognizable protein domains are indicated by red boxes. Amino acid sequence alignments of ATWRAP53, ATSMU2, and ATZCH1 proteins with the corresponding putative orthologs in other plant species and model organisms are shown in Supplementary Fig. S7a–c. a) ATWRAP53 (At2g21520) is 425 amino acids in length and encoded by a single copy gene in Arabidopsis. ATWRAP53 protein contains six WD40 repeat domains. In our screen, we recovered four *wrap53* alleles, all of which contain a mutation in or around 1 of the 6 WD40 domains. b) ATSMU2 (At2g26460) is 585 amino acids in length and encoded by a single copy gene in Arabidopsis. In metazoans, SMU2 is referred to as RED, named after a region rich in Arg (R)/Glu (E) or Asp/Asp (D) repeats, which can also be identified in ATSMU2 close to the N- and C- termini. Our screen recovered 2 *smu2* alleles, both of which contain mutations that are located in a recognizable RED domain. c) ATZCH1 (At1g48950) is a zinc finger protein that is 594 amino acids in length and encoded by a single copy gene in Arabidopsis. ATZCH1 contains one zf-C3Hc domain. Our screen recovered five independent *zch1* alleles.

WRAP53/TCAB1 (WD40 encoding antisense to P53/telomerase Cajal body protein 1) is an evolutionarily conserved WD40 repeat protein that is widely distributed in the plant, fungal and animal kingdoms (Fig. 7a; Supplementary Fig. 7a). To our knowledge, WRAP53 has not yet been investigated in detail in plants nor have *wrap53* mutations been recovered in any prior genetic screen.

In mammalian cells, WRAP53 has been shown to interact with coilin (Machyna *et al.* 2015) and to be essential for CB cohesion (Henriksson and Farnebo 2015). By contrast, we found that a *wrap53* mutation—unlike a *coi1* mutation—does not disrupt CB structure in Arabidopsis, at least in the cell type tested (trichomes) (Supplementary Fig. 8). Furthermore, we did not identify ATWRAP53 as a coilin–interacting protein in a comprehensive IP-MS analysis (Supplementary Table 2). These results suggest that WRAP 53 may act differently in plants than in mammals and not partner directly with coilin to support CB architecture. Additional work in the future is needed to confirm this conjecture and to determine the mechanism by which *wrap53* mutations attenuate the hyper-GFP phenotype in young *coi1 wrap53* seedlings.

#### SMU2 (suppressors of mec8 and unc52) (At2g26460)

SMU2 is a splicing factor that is conserved in most plant and animal species that carry out alternative splicing (examples in Fig. 7b; Supplementary Fig. 7b). Previous studies in plants found that SMU2 is important for constitutive and alternative splicing in maize (Chung *et al.* 2009) and for magnesium homeostasis in Arabidopsis (Feng *et al.* 2020).

In mammalian cells, the SMU2 ortholog, termed RED (Fig. 7b), interacts with the protein SMU1 to form a complex required for spliceosome activation (Keiper *et al.* 2019). Although interactions between SMU1 and SMU2 proteins have been reported in plants (Chung *et al.* 2009; Feng *et al* 2020), we were unable to demonstrate such an interaction using yeast 2-hybrid assays, possibly because the SMU1 protein was unstable in yeast cells under our experimental conditions. Interestingly, however, we did retrieve *smu1* mutants in the previous screen using the *GFP* splicing reporter to identify splicing factors (Kanno, Lin, Fu, Matzke, *et al.* 2017; Kanno *et al.* 2020). Whereas a *smu2* mutant (in a *coi1* background) exhibits a GFP-weak/intermediate phenotype approaching that of the *WT T* line (Fig. 6d), a *smu1* mutant (in a wild-type *COI1* background) displays a hyper-GFP phenotype (Kanno, Lin, Fu, Matzke, *et al.* 2017). The opposite effects of *smu1* and *smu2* mutations on the same *GFP* splicing reporter gene probably reflect differing impacts on the splicing of the 2 mutations in specific genetic backgrounds. In GFP-weak/intermediate *coi1 smu2* double mutants, the unspliced, untranslatable *GFP* pre-mRNA predominates (Supplementary Fig. 4), whereas, in hyper-GFP *COI1 smu1* mutants, the translatable AU-AG transcript is the major splice variant (Kanno, Lin, Fu, Matzke, *et al.* 2017). A further interesting but currently unexplained feature of the splicing defects in *coi1 smu2* mutants is the exceptionally high number of IR events, and to a lesser extent exon skipping (ES) and alternative 5′-splice site events compared to *coi1 wrap53* and *coi1 zch1* mutants (Supplementary Table 4). Further work is required to understand the mechanism of SMU2 in alternative splicing and the suppression of the hyper-GFP phenotype in *coi1* seedlings.

#### *ZCH1* (At1G48950)

ZCH1 is a zinc finger protein containing a zf-C3HC domain (Fig. 7c). Although this domain is widely distributed throughout the eukaryotic kingdom, the ZCH1 protein we identified is specific to plants (Supplementary Fig. S7C). We identified 5 *zch1* alleles in our screen (Fig. 7c), all of which exhibit reduced GFP fluorescence in a *coi1* mutant background (Fig. 6e, 8 DAG). Around 1- to 2-wks after germination, however, the initially GFP-weak/intermediate phenotype of *coi1 zch1* mutants becomes even weaker and infiltrates downward into the hypocotyl (Fig. 6e). Although this silencing pattern is reminiscent of that seen in *dcl4* backgrounds (compare Fig. 3d with Fig. 4c), the predominant size class of *GFP* siRNA associated with silencing differs in each case: 22-nt in the case of *dcl4* (Figs. 3c, 4c, 5b) and 21-nt in the case of *coi1 zch1* mutants (Supplementary Figs. 3a–c). The increased level of 21-nt siRNAs in *coi1 zch1* relative to a *coi1* single mutant and other *coi1* suppressor mutants (Supplementary Fig. 3a–c) hints that the wild-type ZCH1 protein may promote biogenesis and/or accumulation of *GFP* 21-nt siRNAs in a manner that remains to be determined.

During the course of our study, ZCH1 was also identified as MEM1 (*methylation elevated mutant1*) in Arabidopsis using a reverse genetics approach to identify new proteins involved in DNA demethylation (Lu *et al.* 2020). MEM1 was found to act similarly to ROS3 in a ROS1-mediated DNA demethylation pathway to remove methylation from transposons and transgene promoters (Lu *et al.* 2020). MEM1 has also been reported to safeguard against DNA damage (Wang *et al.* 2022). In our system, we did not detect changes in DNA methylation in the upstream enhancer of the *GFP* reporter gene in a *coi1 zch1* mutant (Supplementary Table 3; Fig. 1). Therefore, the *zch1* mutations we identified are not likely to reduce *GFP* expression in *coi1* mutants through a mechanism that involves DNA methylation/demethylation of transcriptional regulatory regions. Conceivably, MEM1/ZCH1 could play roles in multiple processes including PTGS, DNA demethylation, and DNA repair depending on the physiological context in which it acts.

#### IP-mass spectrometry analysis (MS)

To identify putative coilin–interacting proteins, we performed an IP-MS analysis using three epitope-tagged coilin proteins: C-terminal mRED (coilin-mRed); N-terminal FLAG (FLAG-coilin); and C-terminal FLAG (coilin-FLAG) (Supplementary Table 2). This analysis identified several major functional categories of putative coilin-interacting proteins: (1) splicing factors, including many Sm proteins that constitute the SMN complex needed for biogenesis of spliceosomal snRNPs (Hebert *et al.* 2001: Xu *et al.* 2005); (2) histone proteins and histone acetylases/deacetylases, many of which are located in the nucleolus; and (3) nucleolar proteins involved primarily in rRNA processing or modification (Supplementary Table 2). These results are consistent with prominent roles for plant coilin in splicing, nucleolar chromatin structure, and ribosome function (Machyna *et al* 2015). Moreover, they are compatible with the observation that coilin is present in both CBs and nucleoli (Trinkle-Mulcahy and Sleeman 2017).

### Summary of MS-IP analysis and *coi1* suppressor screen

We identified a number of functionally diverse factors in the *coi1* suppressor screen and the MS-IP analysis on coilin protein. These findings reflect the proposed multifarious roles of coilin in plants. Although we found no strict overlap between the proteins identified in the MS-IP analysis and *coi1* suppressor screen, this is not unexpected because the former proteins presumably interact physically with coilin protein whereas those in the latter category are likely to involve functional relationships (for example, acting in the same pathway) that do not necessarily entail a physical interaction. Nevertheless, there was some functional overlap in proteins identified by the two methods. The findings of the *smu2* mutant in the *coi* suppressor screen and numerous Sm proteins and other splicing-related factors in the MS-IP analysis support a prominent role for coilin and CBs in pre-mRNA splicing. In addition, the discovery of the *zch1* mutant in the suppressor screen and its proposed influence on siRNA accumulation is consistent with the roles of coilin in various small RNA-mediated processes as revealed by the IP-MS analysis.

## Conclusions

In this study, we used a *GFP* splicing reporter gene in a *coi1* mutant background to carry out experiments designed to investigate an unusual gene silencing phenomenon appearing in older *coi1* seedlings and to learn more about coilin functions and components of coilin pathways in plants. Our results indicate that delayed *GFP* silencing in older *coi1* seedlings follows a canonical S-PTGS pathway that involves 21–22-nt siRNAs emanating largely from an aberrant splice variant of *GFP* pre-mRNA. The extent to which *coi1* mutations enhance delayed S-PTGS is not yet known. So far, *coi1* is the only hyper-GFP mutant we have identified (Kanno *et al.* 2020) that frequently displays such a striking delayed silencing phenotype, suggesting that the *coi1* mutation somehow sensitizes the *GFP* reporter gene to S-PTGS. We also uncovered the existence of a previously unknown population of endogenous 21–24 nt *GFP* siRNAs in the *WT T* line. These siRNAs are able to induce a moderate level of S-PTGS leading to intermediate *GFP* fluorescence midway between GFP-weak and hyper-GFP phenotypes. The trans-generational stability of intermediate GFP fluorescence in the *WT T* line renders it a valuable tool for identifying mutations that either enhance or suppress GFP expression, and are thus likely to impair factors acting in alternative splicing, RNA silencing and, in a *coi1* mutant background, general coilin functions in plants.

In addition to TGS/RdDM (Kanno *et al.* 2008; Eun *et al.* 2012) and alternative splicing (Kanno *et al.* 2020), the new findings of S-PTGS involvement add a third dimension to processes that can influence the activity of the *GFP* splicing reporter. Our detailed analysis of this system has revealed its versatility and usefulness for studying the contributions of different expression mechanisms and their coordinated influence on the activity of a complex genetic locus.

The *coi1* suppressor screen identified several new proteins that may functionally interact with coilin. We provide foundational information for three of these proteins—WRAP53, SMU2, and ZCH1—all of which remain to be fully characterized in plants. Future study of these proteins is not only of inherent interest but is also potentially valuable for discovering expanded functions of coilin in plants. Seeds of the *WT T* line and the mutants as well as the high-throughput sequencing data we generated are publicly available and provide many resources for the plant scientific community (Supplementary Table 1).

An IP-MS analysis substantiated major roles for plant coilin in splicing, nucleolar chromatin structure, and rRNA maturation. These processes require in some cases small RNAs. (Preuss *et al.* 2000; Kishore *et al.* 2010; Wang *et al.* 2015; Wang and Chekanova 2016; Park *et al.* 2019; Huang *et al.* 2022). The identification of *zch1* mutations in the *coi1* suppressor screen and their enhancement of *GFP* siRNA accumulation provide at least an indirect link between coilin function and siRNA biogenesis and/or stability.

Our cumulative results highlight the involvement of coilin in multiple processes, some of which can be unified through a requirement for small RNAs. These findings lend support to previous proposals suggesting that coilin may facilitate RNP biogenesis by acting as a chaperone for small nuclear noncoding RNAs (Machyna *et al.* 2015). Regarding coilin's participation in alternative splicing, our prior forward genetic screen to identify splicing factors (Kanno *et al.* 2020) represents, to our knowledge, the first in any organism based on an alternatively spliced gene and it is the only forward screen, apart from “*no cajal bodies*” (*ncb*), which retrieved *coi1* mutants (Collier *et al.* 2006). These findings suggest that coilin protein may carry out a special role in specifically alternative splicing in a way that is worthy of further study.

## Supplementary Material

jkad175_Supplementary_Data

## Data Availability

Supplementary Fig. 1 contains information on the GFP protein fold and positions of amino acid substitutions leading to loss of fluorescence; Supplementary Fig. 2 contains data on probable source of pre-existing *GFP* siRNAs in the *WT T* line; Supplementary Fig. 3 shows a comparison of abundances and size classes of *GFP* siRNAs in coilin suppressor screen mutants and other mutants used in this study; Supplementary Fig. 4 shows proportions of three *GFP* splice variants in *coi1-8* and three coilin suppressor mutants; Supplementary Fig. 5 shows coilin domains and positions of amino acid changes in *coi1* mutants; Supplementary Fig. 6 shows reduced accumulation of GFP protein in coilin suppressor mutants; Supplementary Fig. 7 shows amino acid sequence alignments of proteins identified *n* the coilin suppressor screen; Supplementary Fig. 8 shows that a *wrap53* mutation does not disrupt CBs in Arabidopsis; Supplementary Table 1 lists mutants identified in the coilin suppressor screen and other mutants used in this study; Supplementary Table 2 presents a summary of coilin IP-MS experiments; Supplementary Table 3 shows a summary of bisulfite sequencing experiments to detect DNA methylation in the *GFP* upstream region; Supplementary Table 4 shows a summary of differential alternative splicing events in *smu2* mutants. Seeds of the *WT T* line and coilin suppressor mutants as well as other mutants used in this study (Supplementary Table 1) are available from the Arabidopsis Biological Research Center (ABRC, Ohio, USA) and all DNA and RNA sequence data for selected mutants are available at NCBI under the respective accession numbers as follows: ***wrap53*/At4g21520/*wrap53-1*/**ABRC stock number CS72418/NCBI accession numbers: small RNAs: SRR13267755, SAMN17103491; SRR13267754, SAMN17103492; RNAs: SRR13267733, SAMN17103504; SRR13267750, SAMN17103505; SRR13267749, SAMN17103506; DNAs: SRR13267740, SAMN17103515; ***smu2***/**At2g26460**/***smu2-1***/ABRC stock number CS72417/NCBI accession numbers: small RNAs: SRR13267722, SAMN17103489; SRR13267756, SAMN17103490; RNAs: SRR13267729; SAMN17103501; SRR13267730, SAMN17103502; SRR13267731, SAMN17103503; DNAs: SRR13267739, SAMN17103514; ***smu2-1/SMU2/coi1complemented***/ABRC stock number CS72658/NCBI accession numbers: RNAs: SRR14917397, SAMN19865507; SRR14917396, SAMN19865508; SRR14917395, SAMN19865509; small RNAs: SRR14917403, SAMN19865514; SRR14917402, SAMN19865515; ***smu2-2***/ABRC stock number CS72630; ***smu2-3***/ABRC stock number CS72631; NCBI accession numbers: RNAs: SRR14917400, SAMN19865504; SRR14917399, SAMN19865505; SRR14917398, SAMN19865506; small RNAs: SRR14917405, SAMN19865512; SRR14917404, SAMN19865513; ***zch1/*At1g48950**/***zch1-1***/ABRC stock number CS72421; NCBI accession numbers: small RNAs: SRR13267753, SAMN17103495; SRR13267752, SAMN17103496; RNAs: SRR13267746, SAMN17103510; SRR13267735, SAMN17103511; SRR13267736, SAMN17103512; ***zch1-2***/ABRC stock number CS72422; ***zch1-3***/ABRC stock number CS72423; ***zch1-4***/ABRC stock number CS72424; ***zch1-5***/ABRC stock number CS72425; ***WT T***/ABRC stock number CS69640/small RNAs; SRR13267760, SAMN17103474; SRR13267719, SAMN17103475; SRR13267724, SAMN17103476; ***coilin/*At1g13030/*coi1-1***/ABRC stock number CS69632; NCBI accession numbers: small RNAs: SRR13267745, SAMN17103483; ***coi1-8***/ABRC stock number CS69639; NCBI accession numbers: small RNAs: SRR13267720, SAMN17103484; SRR14917394, SAMN19865510; SRR14917406, SAMN19865511; RNAs; SRR14917408, SAMN19865501; SRR14917407, SAMN19865502; SRR14917401, SAMN19865503; ***cwc16a***/**At1g25682**/***cwc16a-1***/ABRC stock number CS69846; NCBI accession numbers: small RNAs: SRR13267751, SAMN17103497; SRR13267726, SAMN17103498; ***cwc16a-1/dcl4***: NCBI accession numbers: small RNAs: SRR13267727, SAMN17103499; SRR13267728, SAMN17103500; ***dcl2*/At3g03300/***dcl2-kas* seeds provided by H. Vaucheret**/*dcl2/dcl4***: NCBI accession numbers: small RNAs: SRR15185391, SAMN20298928; SRR15185390, SAMN20298929; ***dcl3-5***/At3g43920/ABRC stock number CS69179; ***dcl3/dcl4***/NCBI accession numbers: small RNAs: SRR15185389, SAMN20298930; SRR15185388, SAMN20298931; ***dcl4***/At5g20320/*dcl4-12* seeds provided by Scott Poethig: NCBI accession numbers: small RNAs: SRR13267732, SAMN17103477; SRR13267738, SAMN17103478; SRR13267741, SAMN17103479; ***coi1/dcl4***: SRR13267759, SAMN17103485; SRR13267758, SAMN17103486; SRR15185396, SAMN20298924; SRR15185395, SAMN20298925; ***dcl4***: NCBI accession numbers: small RNAs: SRR15185387, SAMN20298932; SRR15185386, SAMN20298933; SRR15185394, SAMN20298934; ***rdr6****/*At3g49500/***rdr6-14***/ABRC stock number CS24288; NCBI accession numbers: small RNAs: SRR13267742, SAMN17103480; SRR13267743, SAMN17103481; SRR13267744, SAMN17103482; ***coi1-1/rdr6-14***: NCBI accession numbers: small RNAs: SRR13267757, SAMN17103487; SRR13267721, SAMN17103488; SRR15185393, SAMN20298926; SRR15185392, SAMN20298927. The mass spectrometry proteomics data have been deposited to the ProteomeXchange Consortium via the PRIDE [1] partner repository with the dataset identifier PXD027146. Supplemental material available at G3 online.
