# Supplementary material for: A GFP splicing reporter in a coilin mutant background reveals links between alternative splicing, siRNAs, and coilin function in Arabidopsis thaliana
Source: G3 (Bethesda). 2023 Aug 4;13(10):jkad175. doi: 10.1093/g3journal/jkad175 (PMC10542627; doi:10.1093/g3journal/jkad175)
Supplement: jkad175_Supplementary_Data [file jkad175_supplementary_data.zip › Figure_S1_G3-2023-404387.pdf]

**Figure S1:** Overall GFP protein fold and positions of amino acid substitutions leading to loss of fluorescence

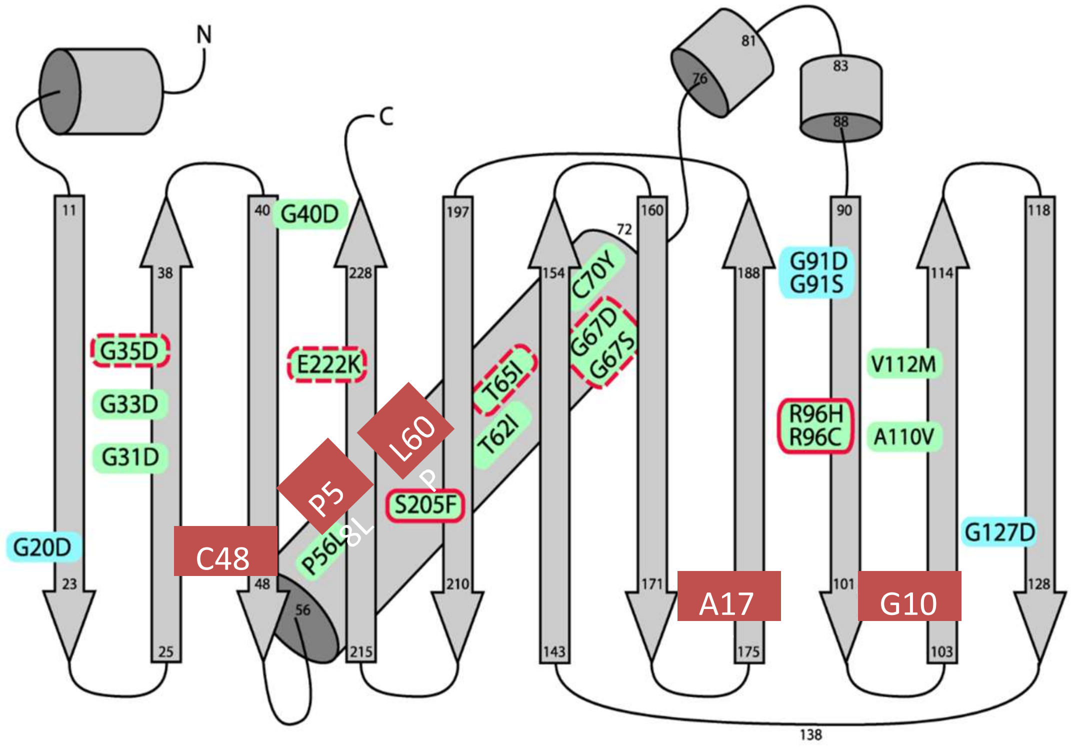

**Figure S1:** Overall GFP protein fold and positions of amino acid substitutions leading to loss of fluorescence (Kanno et al)

Figure and text are adapted from Figure 1 in Fu et al. (2015). The figure shows a schematic depiction of the overall fold of Thr65-GFP protein, the GFP variant that was used in the T-DNA construct (Figure 1 in Fu et al. 2015). Vertical arrows indicate the eleven β strands of the β-barrel structure. Amino acid residue numbers at the base and tips of the arrows denote the beginning and ends of secondary structural elements. The chromogenic tripeptide (Thr65-Tyr66-Gly67) is located on an internal α-helix (diagonal cylinder) extending from amino acids 56 to 72. Amino acid substitutions identified in a previous screen (Fu et al., 2015) that lead to losses of fluorescence are shown on blue, gray or green backgrounds. Solid red outlines indicate substitutions causing defects in chromophore formation without substantial reductions in GFP protein accumulation (Fu et al., 2015). Dotted red outlines designate substitutions resulting in lowered GFP protein levels relative to wild-type. For the remaining substitutions, no GFP protein was detected by Western blotting, indicating these mutations promote instability of the GFP protein (Fu et al., 2015). Lid residues at the N and C termini (G91 and G127) and the opposite side (G20), which is referred to as the 'top' of the barrel (Zimmer et al., 2014), are highlighted in blue.

Noteworthy new mutations identified in the current study (**Table S1**) are shown on red backgrounds. These include G140D, which is present at a hinge region of the GFP secondary structure and thus is likely to be required for GFP folding and stability. Accordingly, G140 is one of 23 highly conserved amino acids in GFP-related proteins (Ong et al, 2011; Fu et al., 2015). A second notable mutation is C58L, which is present in stretch of several proline residues, including C56 that was identified in a previous screen (Fu et al., 2015). These cysteine residues are thought to be important for maintaining the alpha-helical structure necessary for chromophore formation (Fu et al. 2015). The remaining new mutations - L60P. A179T and C48Y - lead to loss of fluorescence by currently unknown means.

The finding of the these new *gfp* mutations add to the growing list of *gfp* loss-of-function mutations, which are proving valuable in various studies for determining amino acid residues important for GFP fluorescence and protein stability, and for using GFP protein as a sensor (Pauly et al., 2017; Birnbaum et al., 2020; Decaestecker et al., 2019).

Birnbaum MD, Nemzow L, Kumar A, Gong F, Zhang F. 2020. A rapid and precise mutation-activated fluorescence reporter for analyzing acute mutagenesis frequency. CELL CHEM BIOL. 27:1220. doi: 10.1016/j.chembiol.2020.08.016.

Decaestecker W, Buono RA, Pfeiffer ML, Vangheluwe N, Jourquin J, Karimi M, Van Isterdael G, Beeckman T, Nowack MK, Jacobs TB. 2019. CRISPR-TSKO: A technique for efficient mutagenesis in specific cell types, tissues, or organs in Arabidopsis. PLANT CELL. 31:2868-2887. doi: 10.1105/tpc.19.00454.

Fu JL, Kanno T, Liang SC, Matzke AJ, Matzke M. 2015. GFP loss-of-function mutations in *Arabidopsis thaliana.* G3: GENES, GENOMES, GENETICS. 5:1849-1855. doi: 10.1534/g3.115.019604.

Ong WJ, Alvarez JS, Leroux IE, Shahid RS, Samma AA, Peshkepija P, Morgan AL, S. Mulcahy S, Zimmer M. 2011. Function and structure of GFP-like proteins in the protein data bank. MOL BIOSYST. 7: 984-992. doi: 10.1039/c1mb05012e.

Pauly MD, Procario MC, Lauring AS (2017) A novel twelve class fluctuation test reveals higher than expected mutation rates for influenza A viruses. Elife 6: e26437. doi: 10.7554/eLife.26437.

Zimmer MH, Li B, R. Shahid RS, Peshkepija Zimmer M (2014) Structural consequences of chromophore formation and exploration of conserved lid residues amongst naturally occurring fluorescent proteins. Chem Phys 429: 5-11.
