# Supplementary material for: A GFP splicing reporter in a coilin mutant background reveals links between alternative splicing, siRNAs, and coilin function in Arabidopsis thaliana
Source: G3 (Bethesda). 2023 Aug 4;13(10):jkad175. doi: 10.1093/g3journal/jkad175 (PMC10542627; doi:10.1093/g3journal/jkad175)
Supplement: jkad175_Supplementary_Data [file jkad175_supplementary_data.zip › Figure_S2_G3-2023-404387.pdf]

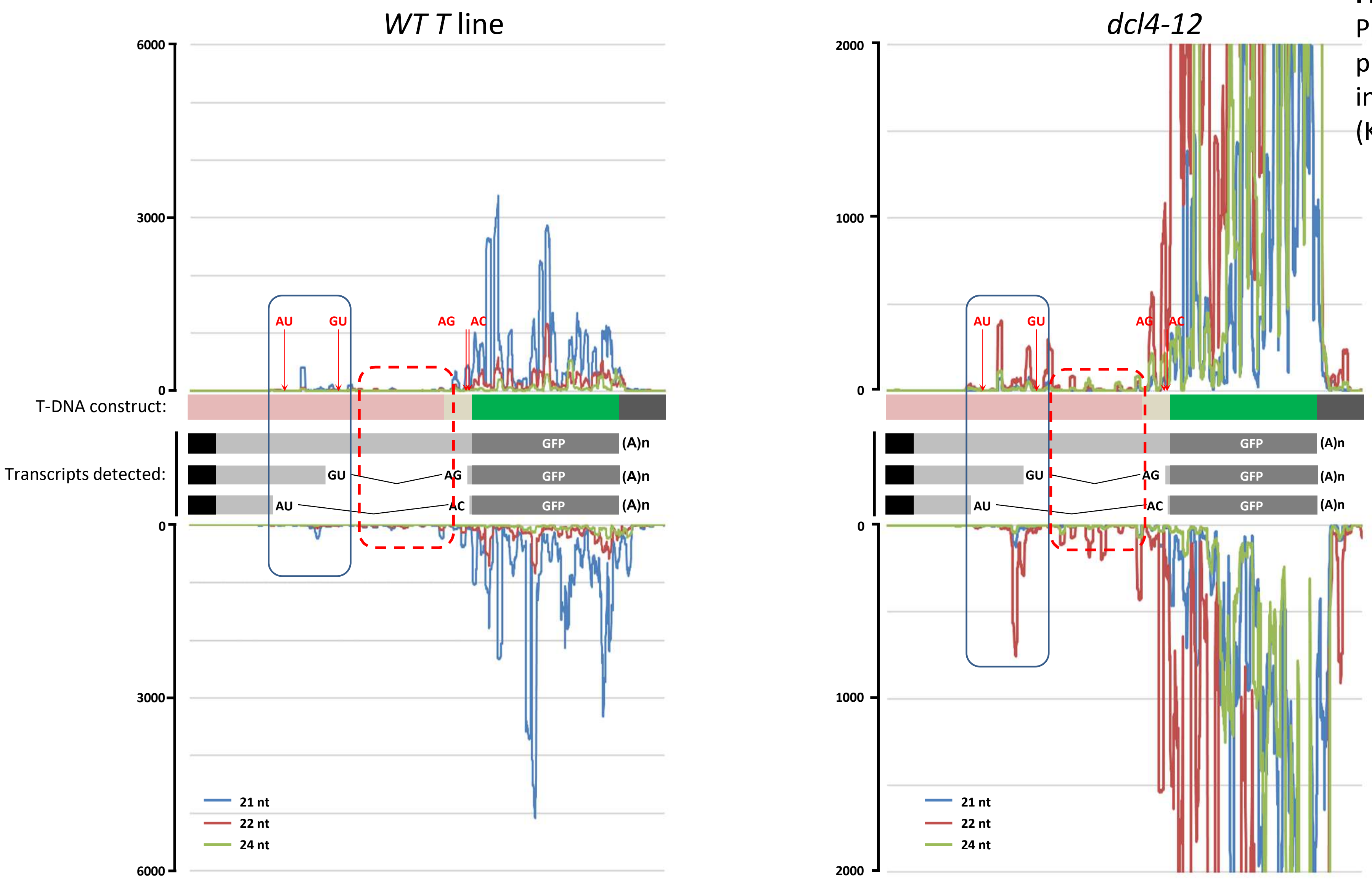

Figure S2:
Probable source of pre-existing GFP siRNAs in the *WT T* line (Kanno et al)

Figure S2: Probable source of pre-existing *GFP* siRNAs in the *WT T* line

We argue here that most of the 21-24 nt *GFP* siRNAs in the *WT T* line and its mutant derivatives, which contain varying levels of all three alternatively spliced *GFP* transcripts (**Figure S4**), may be originating mainly from the spliced, untranslatable GU-AG transcript, with negligible contributions from the unspliced *GFP* pre-mRNA and the spliced, translatable AU-AC variant (*GFP* mRNA) (**Figure 1**). Evidence for this claim can be discerned by inspection of the siRNA distribution profile along the *GFP* CDS and upstream region. To illustrate the point, such results are shown here for the *WT T* line and a *dcl4-12* mutant. Blue, red and green vertical lines represent 21-, 22- and 24-nt siRNAs, respectively. The *GFP* CDS is indicated by the dark gray bars and the upstream region by light gray bars. Positions of splice sites in this region are indicated (also shown in **Figure 1**). Spliced introns are represented by thin black lines.

Negligible contributions to GFP siRNAs from the translatable AU-AC transcript and unspliced pre-mRNA

Although *GFP* siRNAs are derived from the *GFP* CDS, the translatable AU-AC transcript (i.e. *GFP* mRNA) does not appear to contribute significantly to the *GFP* siRNA pool. The best evidence for this claim is provided by the *cwc16a* single mutant, which produces primarily the translatable AU-AC transcript and only minor amounts of the two untranslatable transcripts, and does not accumulate siRNAs (**Figure S3A and B; Figure 5A**) (Kanno et al., 2017a, 2020).

The unspliced *GFP* pre-mRNA can also be ruled out as a prominent source of siRNAs as evidenced by the near absence of siRNAs from its central region (**red dotted box;** *WT T* and *dcl4-12*), which is spliced out in GU-AG and AU-AC transcripts. This demonstrates that the unspliced variant, which accumulates to an appreciable level in all genotypes (**Figure S4**), is not the primary source of siRNAs.

Convincing contributions from the GU-AG splice variant to the GFP siRNA pool

The presence of siRNAs derived from the extended first exon of the untranslatable GU-AG transcript **(solid blue boxes; *WT T* and *dcl4-12*)**, which is not present in the translatable AU-AC transcript, supports the idea that the GU-AG transcript provides the primary substrate for RDR6 synthesis of dsRNA and subsequent processing to siRNAs by DCL activities. The accumulation of siRNAs from the unique first exon of the untranslatable GU-AU transcript

can be seen most clearly in a *dcl4* mutant (*dcl4-12*; **blue box**), which produces large amounts of primarily 22-nt *GFP* siRNAs owing to strong DCL2 activity in the absence of DCL4.

The lower levels of siRNAs from the unique first exon of the GU-AU transcript compared to the *GFP* CDS probably reflect the reduced RDR6 activity as it progresses 3' to 5' along the single stranded aberrant RNA substrate. RDR6 has been reported to read at least 750 nt, with enzyme activity decreasing progressively toward the 5'end of the substrate RNA (Moissiard et al., 2007). In our T-DNA construct, the enhancer region extends for 1277 bp upstream of the minimal 35S promoter (91bp) and *GFP* CDS (720 bp) (**Figure 1**). Hence the unique exon giving rise to siRNAs from the GU-AU transcript is well beyond the optimal range of RDR6, which likely results in less double stranded (ds) RNA from this region and hence a lower quantity of siRNAs.

We assume the *GFP* 21-nt siRNAs (and the 22-nt siRNAs in a *dcl4* mutant background) represent primary siRNAs derived from cleavage of RDR6-dependent synthesis of dsRNAs using the GU-AG transcript as a substrate, although we cannot rule out more complicated scenarios including mechanisms involving secondary siRNAs.
