# Supplementary material for: A GFP splicing reporter in a coilin mutant background reveals links between alternative splicing, siRNAs, and coilin function in Arabidopsis thaliana
Source: G3 (Bethesda). 2023 Aug 4;13(10):jkad175. doi: 10.1093/g3journal/jkad175 (PMC10542627; doi:10.1093/g3journal/jkad175)
Supplement: jkad175_Supplementary_Data [file jkad175_supplementary_data.zip › Figure_S3_G3-2023-404387.pdf]

Figure S3A: Comparison of abundances and size classes of *GFP* siRNAs in coilin suppressor mutants and other mutants used in this study
y-axis: read count per million (Scale 2000, Max. 12000)

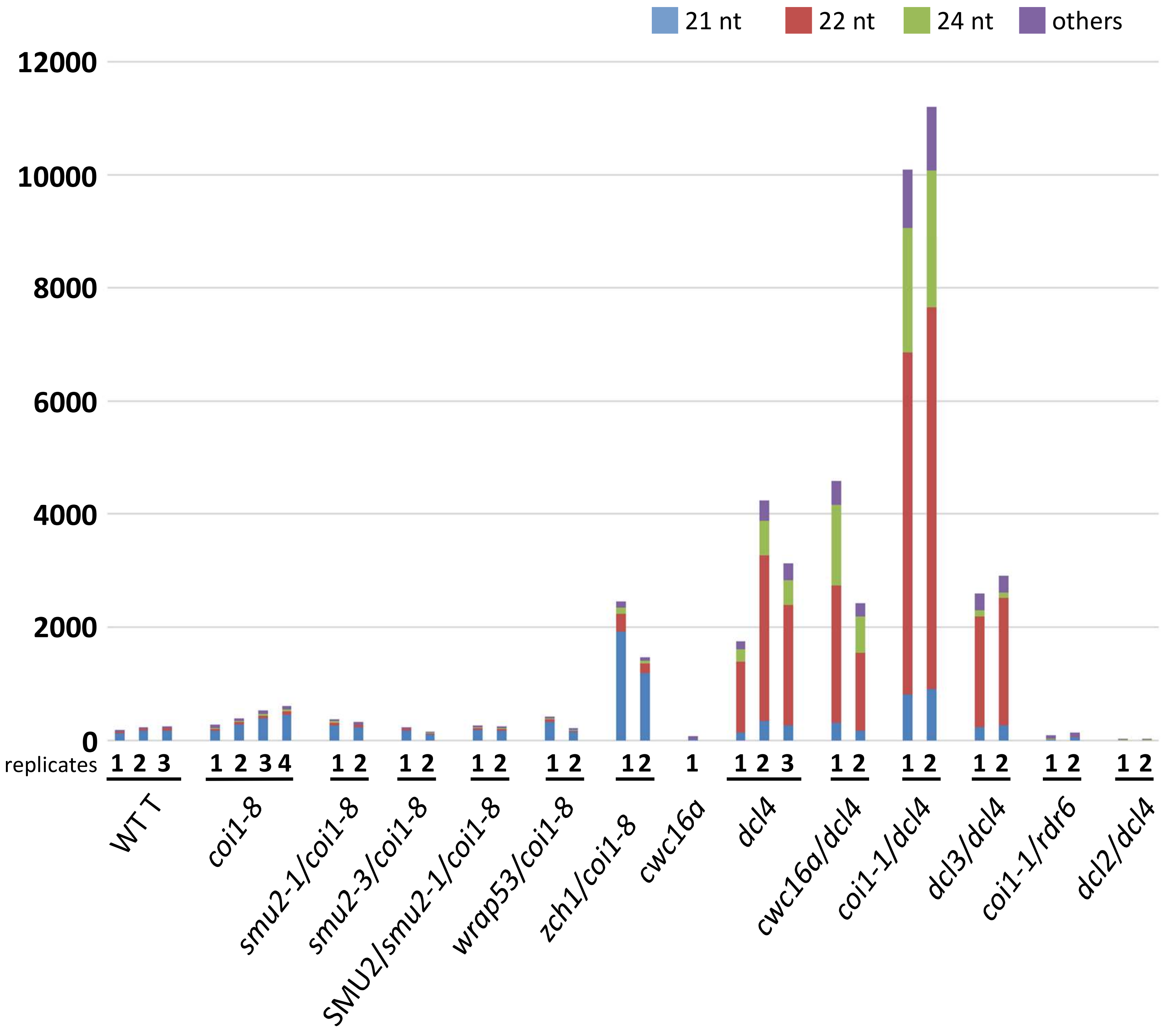

Figure S3B: Comparison of abundances and size classes of *GFP* siRNAs in coilin suppressor mutants and other mutants used in this study
y-axis: read count per million (Scale 200, Max. 2000)

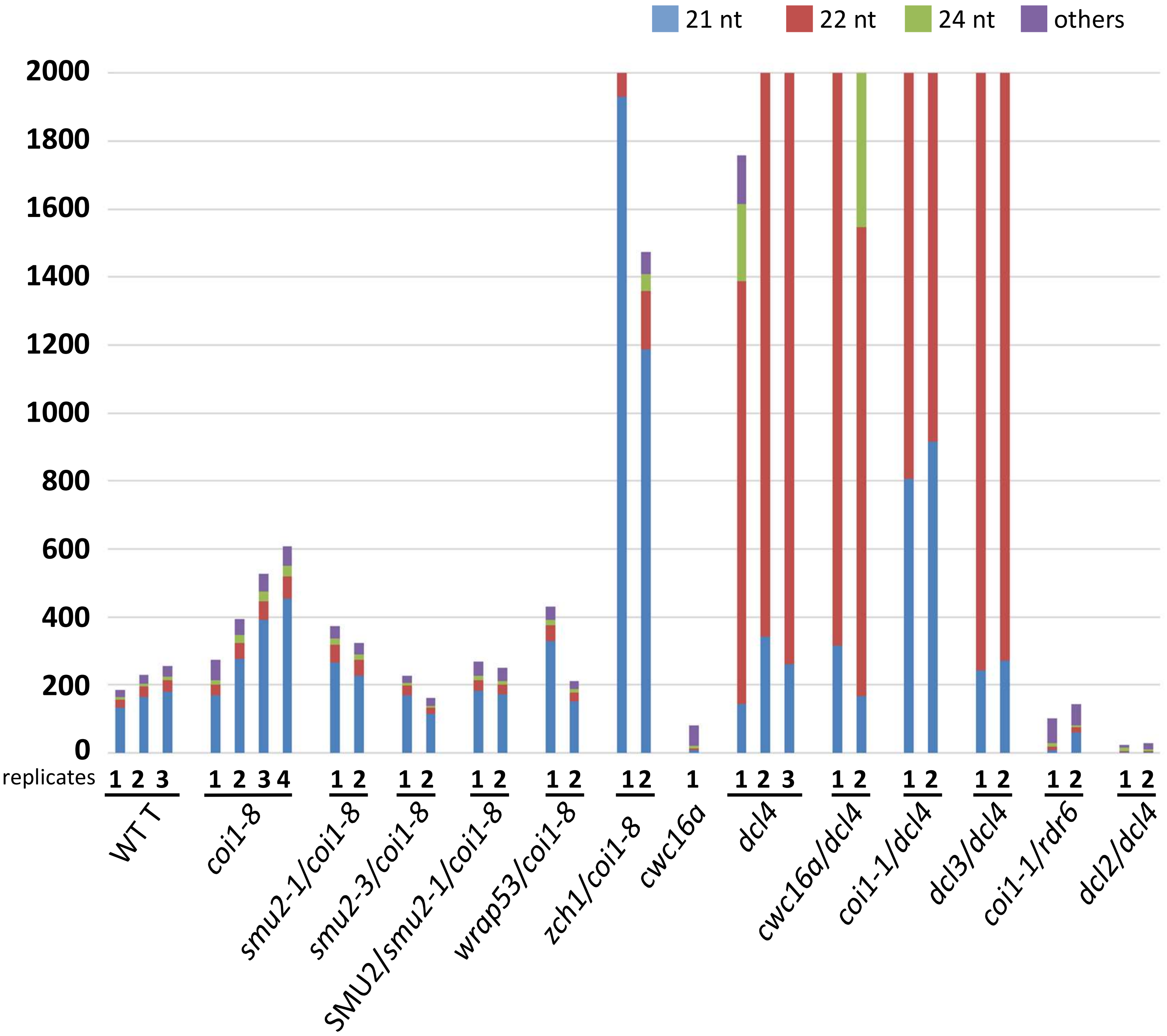

Figure S3C: Comparison of abundances and size classes of *GFP* siRNAs in coilin suppressor mutants and other mutants used in this study
y-axis: ratio (percentage) of each size class in the mutants

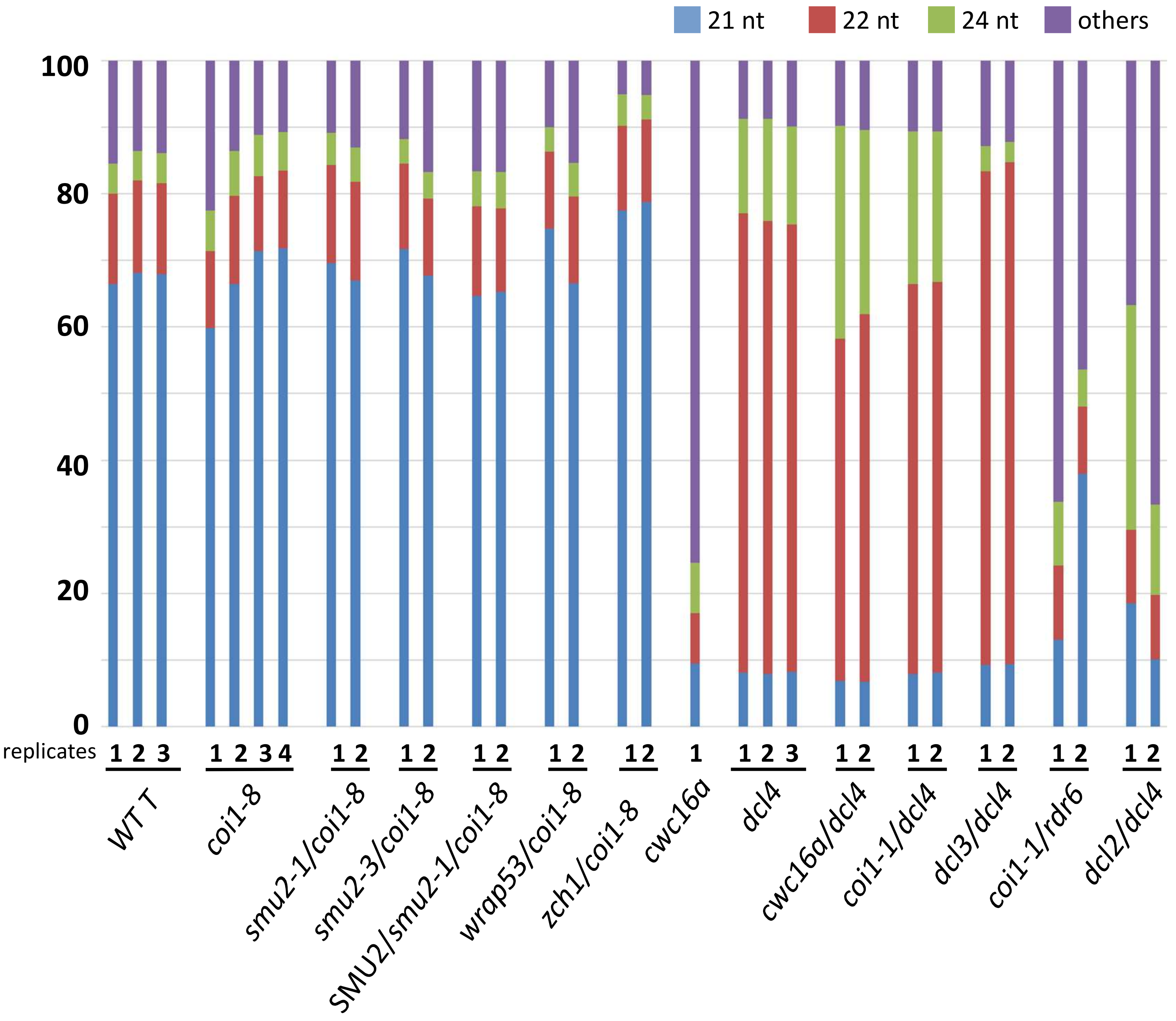

Figure S3: Comparison of abundances and size classes of *GFP* siRNAs in *coi1* suppressor screen mutants and other mutants used in this study (Kanno et al)

The y-axis shows the read count per million reads of *GFP* siRNAs in the indicated mutants. Blue, red and green vertical lines represent 21, 22, and 24-nt siRNAs. respectively. The number of 'replicates' along the x-axis refers to the number of biological replicates. **Parts A** and **B** show the same read data on different scales of maximum reads. The y-axis in **Part C** shows the ratio (percentage) of each size class of siRNA in each mutant. The suppressor mutants are all homozygous for both the indicated suppressor mutation (*smu2-1*, *wrap53-1*, *zch1-1*) and the *coi1-8* mutation.
