# Supplementary material for: A GFP splicing reporter in a coilin mutant background reveals links between alternative splicing, siRNAs, and coilin function in Arabidopsis thaliana
Source: G3 (Bethesda). 2023 Aug 4;13(10):jkad175. doi: 10.1093/g3journal/jkad175 (PMC10542627; doi:10.1093/g3journal/jkad175)
Supplement: jkad175_Supplementary_Data [file jkad175_supplementary_data.zip › Figure_S4_G3-2023-404387.pdf]

Figure S4: Proportions of three *GFP* splice variants in *coi11-8* and three coilin suppressor mutants

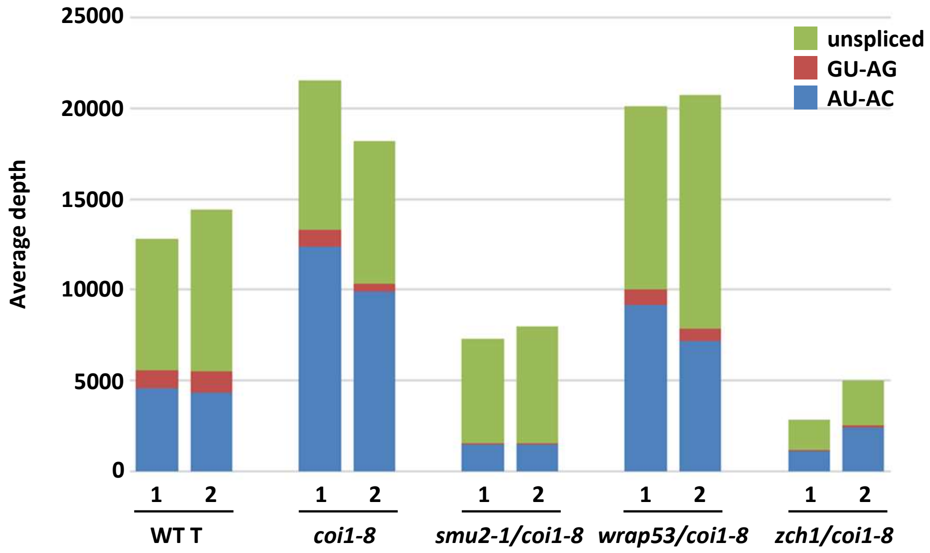

Figure S4: Proportions of three *GFP* splice variants in *coi1-8* and three *coi1* suppressor mutants (Kanno et al)

The y-axis shows the average depth (inferred from the average number of reads covering different regions involving different combinations of *GFP* splice variants) in each of the *GFP* splice variants in the indicated mutants. The x-axis shows the number of biological replicates. Blue bars: AU-AG transcript (spliced, *GFP* mRNA): red bars (GU-AG spliced, untranslatable variant, proposed main source of *GFP* siRNAs); green bars (unspliced *GFP* pre-mRNA). The suppressor mutants are all homozygous for the indicated suppressor mutation (*smu2-1*, *wrap53-1*, *zch1-1*) and the *coi1-8* mutation.
