# Supplementary material for: A GFP splicing reporter in a coilin mutant background reveals links between alternative splicing, siRNAs, and coilin function in Arabidopsis thaliana
Source: G3 (Bethesda). 2023 Aug 4;13(10):jkad175. doi: 10.1093/g3journal/jkad175 (PMC10542627; doi:10.1093/g3journal/jkad175)
Supplement: jkad175_Supplementary_Data [file jkad175_supplementary_data.zip › Figure_S5_G3-2023-404387.pdf]

Figure S5: Domains in coilin protein and positions of amino acid changes in *coi1* mutants

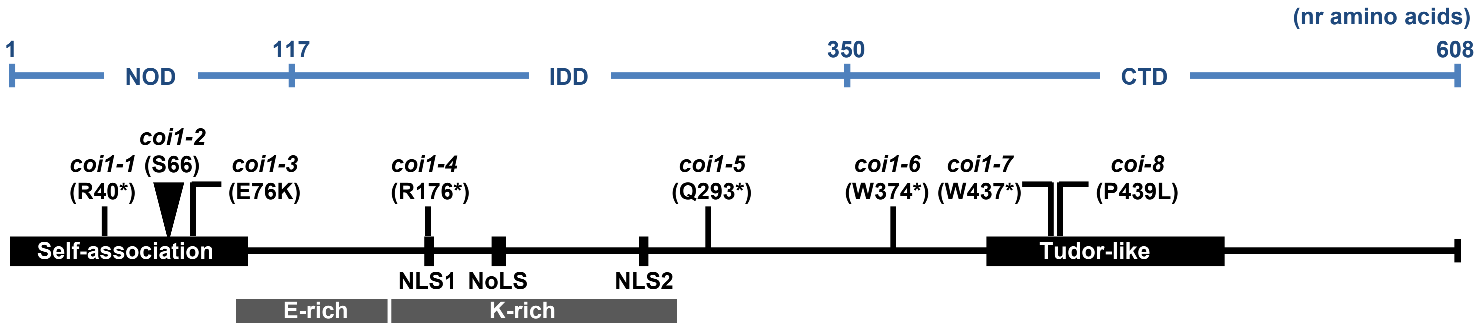

Figure S5: Coilin domains and positions of amino acid changes in *coi1* mutants (Kanno et al)

Figure and text are modified from Figure 2 in Kanno et al. (2016). Recognizable domains and motifs in coilin (At1G13030) include a self-association domain at the N-terminus; an atypical tudor domain at the C-terminus; two nuclear localization signals (NLS); and a nucleolar localization signal NoLS). Analysis of the secondary structure of Arabidopsis coilin revealed three structural domains: NOD, N-terminal globular domain; IDD, internal disordered domain; CTD, C-terminal domain (Makarov et al., 2013). The indicated *coi1* mutations were originally identified as hyper-GFP (*hgf*) mutants in a previous forward screen for factors affecting alternative splicing and expression of the *GFP* reporter gene (Kanno et al., 2016). The *coi1-8* mutant (P439L) was used for the present *coi1*suppressor screen.

Kanno T, Lin WD, Fu JL, Wu MT, Yang HW, Lin SS, Matzke AJ, Matzke M. 2016. Identification of coilin mutants in a screen for enhanced expression of an alternatively spliced *GFP* reporter gene in *Arabidopsis thaliana*. GENETICS. 203:1709-1720. doi: 10.1534/genetics.116.190751.

Makarov V, Rakitina D, Protopopova A, Yaminsky I, Arutiunian A, Love AJ, Taliansky M, Kalinina N. 2013. Plant coilin: structural characteristics and RNA binding properties. PLOS ONE. 8: e5357. doi: 10.1371/journal.pone.0053571.
