# Supplementary material for: A GFP splicing reporter in a coilin mutant background reveals links between alternative splicing, siRNAs, and coilin function in Arabidopsis thaliana
Source: G3 (Bethesda). 2023 Aug 4;13(10):jkad175. doi: 10.1093/g3journal/jkad175 (PMC10542627; doi:10.1093/g3journal/jkad175)
Supplement: jkad175_Supplementary_Data [file jkad175_supplementary_data.zip › Figure_S6_G3-2023-404387.pdf]

Figure S6: Reduced accumulation of GFP protein in *coi1-8* suppressor mutants

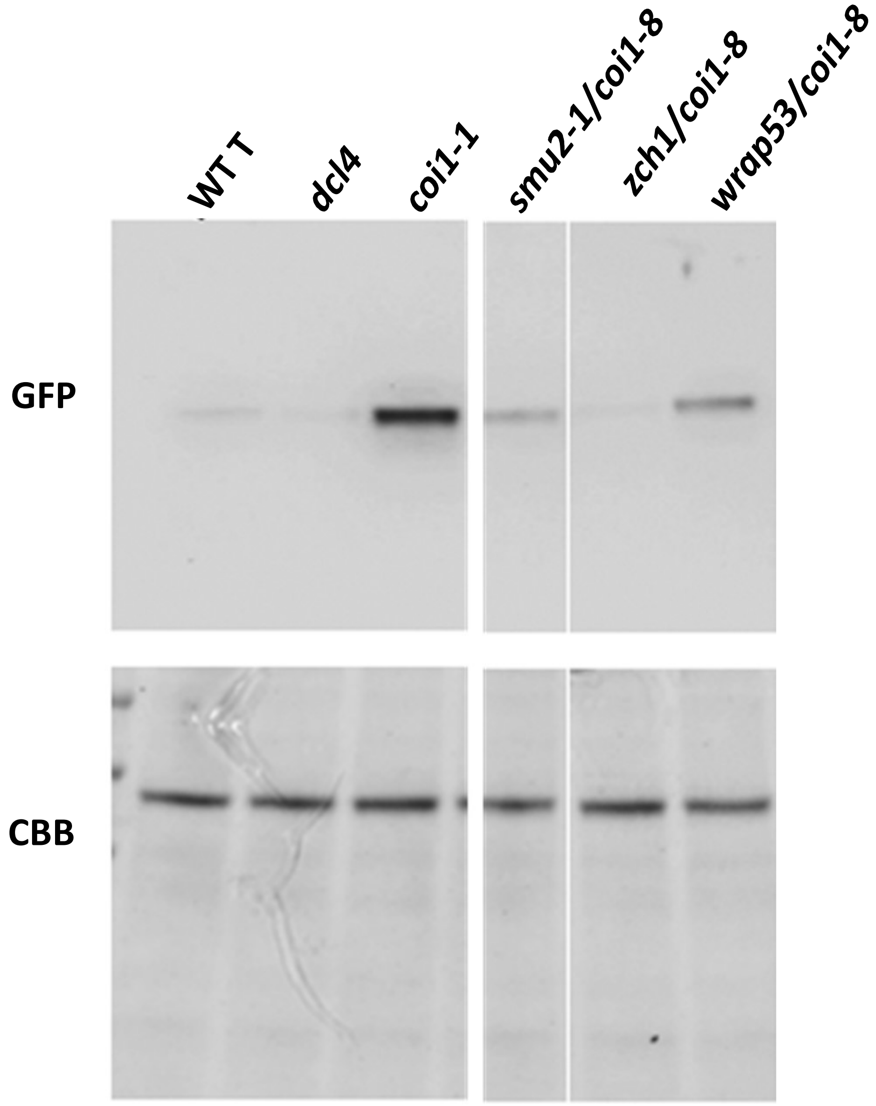

Figure S6: Reduced accumulation of GFP protein in coilin suppressor mutants (Kanno et al)

Western blots were used to detect GFP protein levels in the indicated GFP-weak *coi1* suppressor mutants (which are homozygous for the *coi1-8* mutation) compared to the *WT T* line and the *coi1* hyper-GFP single mutant. Proteins were isolated from two-week-old seedlings with the indicated genotypes and further processed for Western blotting as described in previous publications (Fu et al., 2015; Kanno et al., 2020). The Western blot was probed with monoclonal antibodies to GFP (Roche, catalog nr. 11814 460001). For a loading control, a duplicate gel containing the same samples was run and stained with Coomassie Brilliant Blue (CBB). The coilin suppressor mutants all contain less GFP protein than the single *coi1* mutant and approach the level observed in *WT T* seedlings.

Fu JL, Kanno T, Liang SC, Matzke AJ, Matzke M. 2015. GFP loss-of-function mutations in *Arabidopsis thaliana.* G3: GENES, GENOMES, GENETICS. 5:1849-1855. doi: 10.1534/g3.115.019604.

Kanno T, Venhuizen P, Wu MT, Chiou P, Chang CL, Kalyna M, Matzke AJM, Matzke M. 2020. A collection of pre-mRNA splicing mutants in *Arabidopsis thaliana*. G3: GENES, GENOMES, GENETICS 10:1983-1996. doi: 10.1534/g3.119.400998.
