# Supplementary material for: A GFP splicing reporter in a coilin mutant background reveals links between alternative splicing, siRNAs, and coilin function in Arabidopsis thaliana
Source: G3 (Bethesda). 2023 Aug 4;13(10):jkad175. doi: 10.1093/g3journal/jkad175 (PMC10542627; doi:10.1093/g3journal/jkad175)
Supplement: jkad175_Supplementary_Data [file jkad175_supplementary_data.zip › Figure_S7_G3-2023-404387.pdf]

(A)WRAP53_model species

```
A.thaliana        1 ------------------------------------------------------------
S.cerevisiae      1 ------------------------------------------------------------
C.elegans         1 ---------------------------------------------------------MNT
D.melanogaster    1 MIENSFKMEMHFKNRWQAASLFLRYPRTVTLCLSGKSGDMDESVNLSTSMGDVEASMLNH
D.rerio           1 ----------------------MSGVAQSGEGAAGQEAEADGEP---------PHEALVQG
M.musculus        1 ----------------------MKTSEERLLAPDSLPPDLAPAP--------VPQGSPAEK
H.sapiens         1 ----------------------MKTLETQPLAPDCCPSDQDPAPAHP----SPHASPMNK

A.thaliana        1 ------------------------------------------------------------
S.cerevisiae      1 ------------------------------------------------------------
C.elegans         4 --EESS--SSQELEEKKPSLRAERCGIASLLSSL------------------RSD-----
D.melanogaster   61 NVSFSSTMVTSNGDLSLPKLSVT-------------------L--SAEEILKLRSG-----
D.rerio          31 ALEEE-----EEPAAKQPRLQQED----------------VALDFPMIEIAHEPSEEKSKS
M.musculus       32 NTDFEP-VPPPCGGDDQPQLATDPVASLVVSQELQQGDSV----PLEVEFNTSSE-----
H.sapiens        35 NADSEL-MPPPPERGDPPRLSPDPVAGSAVSQELREGDPVSLSTPLETEFGSPSE-----

A.thaliana        1 ----------------------------------------------------MGEEEAVAE
S.cerevisiae      1 ------------------------------------------------------------
C.elegans        37 HLASKIDELSTTEAAASEEIPEEIPENRPLNRKE---------KRKLQRMQEIDQEVK---
D.melanogaster   96 ----RPKNAVE---SPHA----GVPMETSLAAEE--EANGDEEEESVRVVDIEDTLEL-T
D.rerio          71 QDAAGVEEEVS---YHQTDEPVHVAEHAEMERDNEWHQNGAESRE-----MLEQTEEN-T
M.musculus       82 -LSPGIEEQ--------------DVSEHASLPGEE-------------TNLPELESGEAT-E
H.sapiens        89 -LSPRIEEQ--------------ELSENTSLPAEE---ANGSLSEEEANGPELGSGKAM-E

A.thaliana       10 ENGGLKVESGEQKSSWPTMRFDVSP-YRTHHFS----------KQFRTARNPNNFLKGLK
S.cerevisiae      1 -----------------------------------------------MEKKVICQDIF
C.elegans        86 ------------KKAQEPVEIAEIPTNLKNTFCDKATFNSYNAQFGYKSTENNNFVHFSI
D.melanogaster  142 SNGNE-AKPEDQE-----LAIAPVLQYFQGALVEL------GRRCWTSSTEAQHYTKGCY
D.rerio         122 ADEPEKNSPEAEQHYQG-LDFRQNPQMLTGSW--------------AEYTHSAENYLRGCK
M.musculus      115 GVSEERAEVDEGDTFWT-YSFSQVPRYLSGSW--------------SEFSTRSENFLKGCK
H.sapiens       131 DTSGEPAAEDEGDTAWN-YSFSQLPRFLSGSW--------------SEFSTQPENFLKGCK

A.thaliana       59 WSPDGSCFLASSEDNTLSLFHLPQDGGDSNGYGVPVPEEDSYGASLLVNEGESVYDFCWY
S.cerevisiae     12 WSCDGTSFVSVHNDFGIRQYLVPEESNTDKLN------RNLILPFTRFFRNQSIVSCAID
C.elegans       134 QNEQGNRALVASQDRFIRMYKIDET-----------------PEVIWKHNTGNLVLDSCWE
D.melanogaster  190 WSPDGTCLLVPVHLDGMHVIEMPSDLYSADT-VQPARSLTKLQSEVHVPEGGTVYDCVWY
D.rerio         168 WAPDGSCIVSNSADNVLRVYNLPAELYSSQ-WDL----LSEMSPVLKMAEGDTIYDYCWF
M.musculus      161 WAPDGSCILTNSADNVLRIYNLPPELYSEQ-EQVD---YAEMVPVLRMVEGDTIYDYCWY
H.sapiens       177 WAPDGSCILTNSADNILRIYNLPPELYHEG-EQVE---YAEMVPVLRMVEGDTIYDYCWY

A.thaliana      119 PYMSVSD-----PLTCVFATSTRDHPIHLWDSTSGELRCTYRAYDAMDE-ITAAFSVGFN
S.cerevisiae     66 PFYTLYNENSDRLAGDRIVVGGKNFPLQLYSLMDGQCILSYDTMNKINGEYETVYSVKID
C.elegans       178 NSGK------------GVFSSSKLRPIQLFDTENGSILGAYNGKDAGDN-ITAAMSIG--
D.melanogaster  249 PHMNSLQ-----PETCLWLATRQHEPIHMWDAFDGSLRCSYSGYDAVDE-VMAAISLAFS
D.rerio         223 PKMTSTD-----PDTCFIASSSRDNPVHIWDAFYGDLRASFRPYNHLDE-LTAAHSLCFS
M.musculus      217 SLMSSTQ-----PDTSYVASSSRENPIHIWDAFTGELRASFRAYNHLDE-LTAAHSLCFS
H.sapiens       233 SLMSSAQ-----PDTSYVASSSRENPIHIWDAFTGELRASFRAYNHLDE-LTAAHSLCFS

A.thaliana      173 PDGTKIFAGYNSSIRVFDLHRPGRDFRQYSTLQK-NKEGQAGILSTLAFSPTNS-----G
S.cerevisiae    126 VESRVYTGSCRNKVAIYDKSRRDAVWMNQSTKKA--SKGRQSIISCFEEQPMGGQALSRG
C.elegans       223 QSGSSLIGGFKNKFQIWDIEYTGDAIQHIKSFDNDYNTGTTGLPMSITPHPTMP-----D
D.melanogaster  303 HDGEQIYAGYKRCIKIFDTSRPGRFCDDYPVKFA---------ISCIAQTTAHP-----H
D.rerio         277 PDGSQLYCGFDKIVRVFHTDRPGRDCEQRPTMVK--KRGQTGIISCIAFSQCH------S
M.musculus      271 PDGSQLFCGFNRTVRVFSTSRPGRDCEVRATFAK--KQGQSGIISCIAFSPSQ------P
H.sapiens       287 PDGSQLFCGFNRTVRVFSTARPGRDCEVRATFAK--KQGQSGIISCIAFSPAQ------P
```

```
A.thaliana       227 MLAVGSYGQTTG---IYREDNMELLYVLH-GQEGGVTHVQFSKDG---NYLYTGGRKDPY
S.cerevisiae     184 SLLCGSYANEMFQ--VDCRHQRLERLNYTRTVAGGIVQILTSDNG---RYVYVVRRNSDA
C.elegans        278 LFAAGGSSSLVA---IYSLKWRNAVSTIE-GSLKGYTNLHFSPDG---LKLYASERKG-D
D.melanogaster   349 TLTCGNWHGYIQHFDLRCSHKQGPLFTLG-GHKGGITQLRYGEFGNGEWHLFSGARKCDK
D.rerio          329 MYACGSYSRSVG---LYSCDDGSLLALLPTRHHGGLTHLLFSPNG---YHLYTGGRKDSE
M.musculus       323 LYACGSYGRTIG---LYAWDDGSPLALLG-GHQGGITHLCFHPDG---NLFFSGARKDAE
H.sapiens        339 LYACGSYGRSLG---LYAWDDGSPLALLG-GHQGGITHLCFHPDG---NRFFSGARKDAE
```

```
A.thaliana       280 ILCWDIRKSVEIVYKLYRA----TENTNQRVFFDIEPCGRHLGTGGQDGLVHMYDLQTG-
S.cerevisiae      239 ISIYDRRNLQHELNVLRLPFRIHHNSAKLKAYID---TAYGLSMGTPQGTILNWGRDLVE
C.elegans        330 IHCFDTRMNM--LTQILKR----DMTATHRTRFSIDKSGRLLFSGTSGGDVIVYDLHEY-
D.melanogaster  408 ILQWDMRNYKQPLVELQRH-----VDTNQRIQFDLASDSNWLASGDTRGFVNVWDLKKY-
D.rerio         383 ILCWDLRDPGQVLFSMQRN-----VNTNQRIYFDLDQSGRYLLSGDTDGVVSVWDTLTA-
M.musculus       376 LLCWDLRQPGHLLWSLSRE-----VTTNQRIYFDLDPSGQFLVSGNTSGVVSVWDISGA-
H.sapiens        392 LLCWDLRQSGYPLWSLGRE-----VTTNQRIYFDLDPTGQFLVSGSTSGAVSVWDTDGP-

A.thaliana       335 -N---------WVS--GYQAASDTVNAFSFHPYLPMAATSSGHRRFAIPDDDDGEDKN--
S.cerevisiae      296 FGGVPSHNSVEDPLITSIPPESEWRTNLDSTIPATVVKNCPGDPELFALSHG--------
C.elegans        383 -SE-----ELQPAH--VQNVASRCVPCVDLKGKK--LVLCSGERVYPDDKLLGDQDQEIC
D.melanogaster  462 -G---------DPS--VLPLHSDCCNGVALNPAMPILATSSGQFHFTDQSAQGDNVTLNG
D.rerio         437 -LPDGNEEILKPLL--QFQAHTDCTNGISVHPFMPLMASSSGQRKFFWPSDSEDSTSD--
M.musculus       430 -LS--DDSKLEPVV--TFLPQKDCTNGVSLHPTLPLLATASGQRVFPEPTNSGDEGEL--
H.sapiens        446 -G---NDGKPEPVL--SFLPQKDCTNGVSLHPSLPLLATASGQRVFPEPTESGDEGE---

A.thaliana       381 ----ELQLKAD----------ENCVSLWSFY-VEDNT-YDENNGVASESHLQNVTEEIVL
S.cerevisiae      348 ----------------------GTISLCRFGG*---------------------------
C.elegans        433 ---------------MDHERSENSFQIFEIQN----------------------------
D.melanogaster  510 TETTELPAPADVNQNQKEVLYENAVVMWWCGQTG--------------------------
D.rerio         492 ----SEEGNVM---PTNGDRPDNALVLWWAGPVASTNEIRQEEE-------PAVIDS---
M.musculus       483 ----ELELPLL---SLCHAHPECQLQLWWCGGGPDPSSPVDDQDEKGQRRTEAVGMS---
H.sapiens        497 ----ELGLPLL---STRHVHLECRLQLWWCGGAPDSSIPDDHQGEKGQGGTEGGVGELI-

A.thaliana       425 T
S.cerevisiae          -
C.elegans            -
D.melanogaster       -
D.rerio             -
M.musculus           -
H.sapiens            -
```

Figure S7: Amino acid sequence alignments (Kanno et al)
(A)WRAP53_plant species

```
A.thaliana         1 --------------MGEEEAVA----------------EENGGLK-V---ESGEQKSS-W
C.reinhardtii      1 --------------MEAEEQYLER---GEPVDA-----GVLSGEQVEVPAEGQHTAAGWY
S.moellendorffi    1 ---------------------------------------------MEEEKAAAPNSYLWC
P.patens           1 --------------MGM---------------------ADDDG---QEHTEPEQSAYY-W
Z.mays_1           1 --------------MAAAQEEAAAAPPSTESDATETEEAPPGGAESDATAMEAAAEYS-W
Z.mays_2           1 --------------MAAAQEEAAAAPPSTESDATETEEAPPGGAESDATAMEAAAEYS-W
O.sativa           1 ------------------------------------------------------------
A.trichopoda       1 --------------MLEDE------------------NIEPTTIQ-SASKEGEYAALF-W
G.max              1 MKASSTSSVVVAVAMGEEEPEV---LESE-S------NVVNANGN-S---SNSNEEYS-F
S.lycopersicum     1 --------------MGEEASEQQLVIESMDEDAVELPETPIEAVE-E---KPSEEPYP-W
V.vinifera         1 --------MSVTEAMEEGEEEQPN--LGD--------EAEEIGNE-A---KQVEEEYS-W
G.raimondii        1 --------------MGEEEREEENQLPSS--------QIEPTETS-E---SPKQQEYE-W
P.trichocarpa      1 --------------MEEEA-DQEHEHEQE--------QEQEHISP-T---ATTQQDYT-W


A.thaliana        26 PTMRFDVSPYRTHHFSKQFRTAR------NPNNFLKGLKWSPDGSCFLASSEDNTLSLFH
C.reinhardtii     39 TAFVFPCQPQPLYRATQEYASSSA---ACPNTNFLKGLKWSPDGACLLTASDDNWLRLYD
S.moellendorffi   16 PRVHFDPPPQRLYHLHRQFRLDG------SGNNFFKGVKWSPDGSCFLTCNEDKRLRIFD
P.patens          22 PVLDFQTPPARRYHFYKQMWSPS------AHNNFFKGAKWSPDGSCFITSSEDNSLRIFD
Z.mays_1          46 LQLRFDRPPRRLYHFARQFRSATSSGSNSSGENFLKGVKWSPDGSSFLTSSDDNSLRLFY
Z.mays_2          46 LQLRFDRPPRRLYHFARQFRSATSSGSNNRRKRTLRRSPLPR-------PLSKVKVRFWF
O.sativa           1 ----MAAEDNPGYALRAT---------LAGHRRAVSAVKFSPDGRLLASASADKLLRVWS
A.trichopod       27 PALRFDEPPQRLYHFHHQFKRAS------HSNSFLKGVKWSPDGSFFLTNSDDNKLRLFD
G.max             46 PVLRFDVSPHRTYHFHRQFITPS------NPNNFFKAVKWSPDGSCFLTSSDDNTLRLFA
S.lycopersicum    42 PVIQYDVPPYLTYHFFNQFRTPS------NPNNFLKGVKWSPDGSGFLTCSDDNTFCLYN
V.vinifera        38 PVIRFDVPPHRAYHFCHQFRTPS------NPNNFLKGVKWSPDGSCFLTSSEDNTLRLFS
G.raimondii       34 PKIRFSVPPYRTYHFHNQFRTGP------NPNNFLKGVKWSPDGSCFLTSSEDNTLRLFY
P.trichocarpa     33 PVIRYNLPPQRTHHFFKQFRTS--------PNNFLKGIKWSPDGSCFLTSSEDNTLHSFS


A.thaliana        80 LPQDGGDSNGY---------------------------G--VPVPEEDSYGASLLVNEGE
C.reinhardtii     96 LPQDVATAAVLQHPDTLPPEEQAEAAEAAAGASTSGSSCSAAGGAGPDNLAPALRMHAGE
S.moellendorffi   70 LPSNALEVEAL---------------------------D--DLAAKTDPLVSSLTVSEGE
P.patens          76 LPSDVLESAVLY--------------------------D--NVKSDEDSNGAALIVDEGE
Z.mays_1         106 LPEEAYIAAEP-------------------------VAE--AAVESEDSYGAFIQANEGE
Z.mays_2          99 LSCL-------------------------------------LLRLWEYSYGAFIQANEGE
O.sativa          48 TSDLASPVAEL--------------------------------------------AGHGE
A.trichopoda      81 LPTNPDGSISDQYNT------------------T--GST--LVSAPSDSYDAKVIVHEGE
G.max            100 PPGTESDAPVAASGH------------------GDGNHE--SSLFVADSFAANVVMHEGE
S.lycopersicum    96 LPYDESGQLADF--------------------------S--SSAADTDSYAASLVMSDGE
V.vinifera        92 LPENGSDHHESA--------------------------C------DEDSYAASIVVSEGE
G.raimondii       88 LPDDGSADQITA--------------------------C--SSVSEEDSYAAQLVVSEGE
P.trichocarpa     85 LPDNGSGSDADG--------------------------C--CLAIDEDSYDASLIVKEGE


A.thaliana       111 SVYDFCWYPYMSVSDPLTCVFATSTRDHPIHLWDSTSGELRCTYRAYDAMDEITAAFSVG
C.reinhardtii    156 TVYDYCWYSRMSALDPVSCCLASTARGHPIQLWDACSGEPRATYRGYNDADEPTAAYSMA
S.moellendorffi  101 TVYDFCWYPHMVATDLSTCVFATTTRDHPVHLWDAATGNLRCSYRAYNAMDEVTAALSVA
P.patens         108 AVYDFCWYPCMTTTDSSTCIFATSTRDHPVHLWDAVSGQLRCTYRAYDAMDEITAAYSIA
Z.mays_1         139 PVYDFCWYPCMSLSDPATCVFASTSRDHPIHLWDATSGELRCTYRAYDAMDEITAALSIS
Z.mays_2         122 PVYDFCWYPCMSLSDPATCVFASTSRDHPIHLWDATSGELRCTYRAYDAMDEITAALSIS
O.sativa          64 GVSDLAFS-------PDGRLIASASDDRTVRIWDLGDGGG--------------------
A.trichopoda     119 SVYDYCWYPYMSSSDPTTCVFATTTRDHPIHLWDAVSGQLRCTYRAYDSMDEIAAAFAIA
G.max            140 SIHDFCWYPYMSSSDLVTNVFATTTRDHPIHLWDATSGQLRCTYRAYDAMDEITAAFSIA
S.lycopersicum   128 SVYDYCWYPYMSSSSPETCVFASTTRDHPIHLWDATTGQLRCTYRAYDAMDEITAAFSIA
V.vinifera       120 SIHDHCWYPYMSASDPVTCVFASTTRDHPIHLWDAASGELRCTYRAYDAVDEITTAFSIA
G.raimondii      120 SVYDFCWYPYMSA--SVTCVFATTTRDHPIHLWDATSGLLRCTYRAYDAVDEITAAFSVA
P.trichocarpa    117 SVYDYCWYPYMTASDPVSCVFATTTRDHPIHLWDATSGLLRCTYRAYDAVDEITAAISIA
```

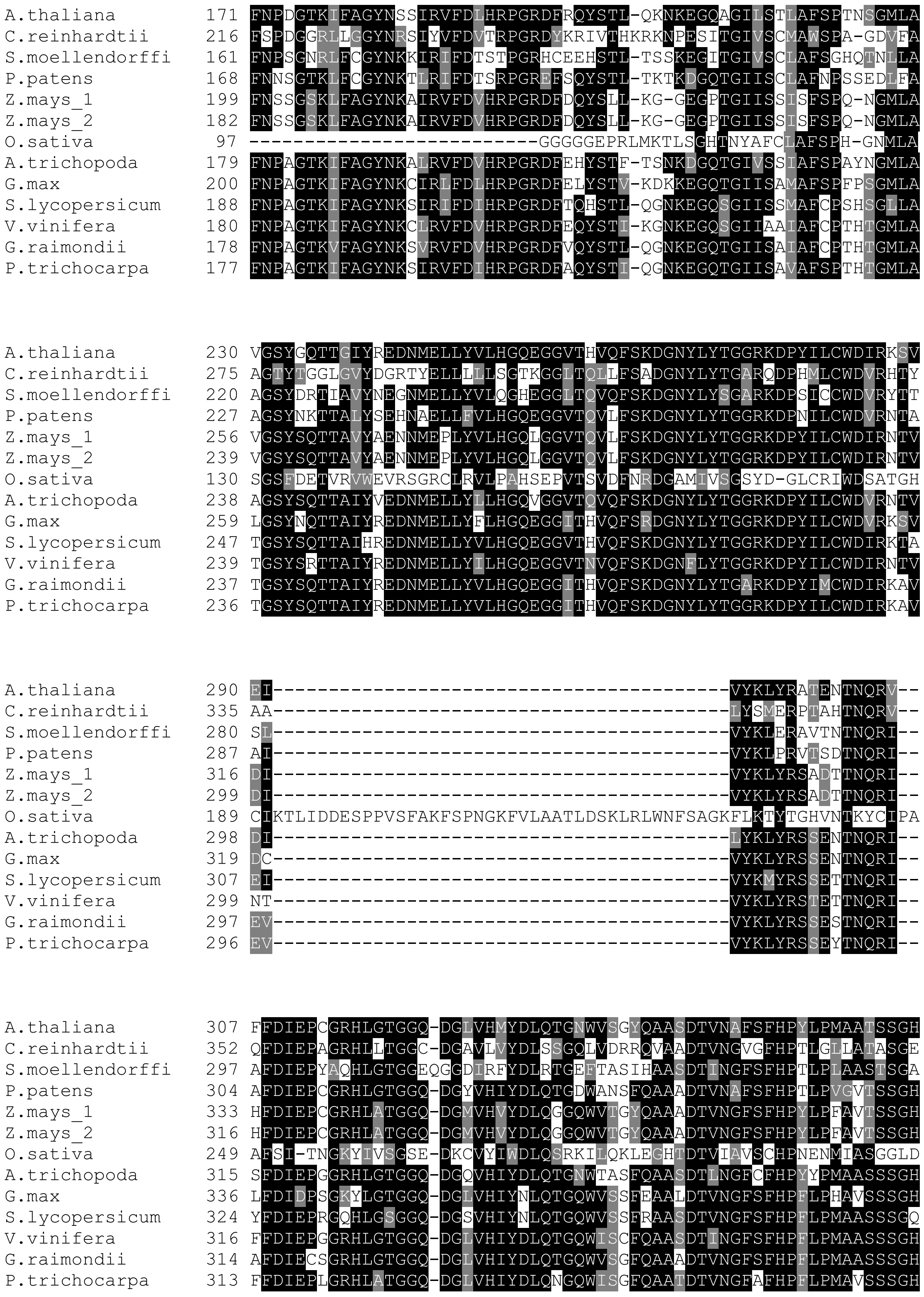

A.thaliana 171 FNPDGTKIFAGYNSSIRVFDLHRPGRDFRQYSTL-QKNKEGQAGILSTLAFSPTNSGMLA
C.reinhardtii 216 FSPDGGRLLGGYNRSIYVFDVTRPGRDYKRIVTHKRKNPESITGIVSCMAWSPA-GDVFA
S.moellendorffi 161 FNPSGNRLFCGYNKKIRIFDTSTPGRHCEEHSTL-TSSKEGITGIVSCLAFSGHQTNLLA
P.patens 168 FNNSGTKLFCGYNKTLRIFDTSRPGREFSQYSTL-TKTKDGQTGIISCLAFNPSSEDLFA
Z.mays_1 199 FNSSGSKLFAGYNKAIRVFDVHRPGRDFDQYSLL-KG-GEGPTGIISSISFSPQ-NGMLA
Z.mays_2 182 FNSSGSKLFAGYNKAIRVFDVHRPGRDFDQYSLL-KG-GEGPTGIISSISFSPQ-NGMLA
O.sativa 97 --------------------------GGGGGEPRLMKTLSGHTNYAFCLAFSPH-GNMLA
A.trichopoda 179 FNPAGTKIFAGYNKALRVFDVHRPGRDFEHYSTF-TSNKDGQTGIVSSIAFSPAYNGMLA
G.max 200 FNPAGTKIFAGYNKCIRLFDLHRPGRDFELYSTV-KDKKEGQTGIISAMAFSPFPSGMLA
S.lycopersicum 188 FNPAGTKIFAGYNKSIRIFDIHRPGRDFTQHSTL-QGNKEGQSGIISSMAFCPSHSGLLA
V.vinifera 180 FNPAGTKIFAGYNKCLRVFDVHRPGRDFEQYSTI-KGNKEGQSGIIAAIAFCPTHTGMLA
G.raimondii 178 FNPAGTKVFAGYNKSVRVFDVHRPGRDFVQYSTL-QGNKEGQTGIISAIAFCPTHTGMLA
P.trichocarpa 177 FNPAGTKIFAGYNKSIRVFDIHRPGRDFAQYSTI-QGNKEGQTGIISAVAFSPTHTGMLA

A.thaliana 230 VGSYGQTTGIYREDNMELLYVLHGQEGGVTHVQFSKDGNYLYTGGRKDPYILCWDIRKSV
C.reinhardtii 275 AGTYTGGLGVYDGRTYELLLLLSGTKGGLTQLLFSADGNYLYTGARQDPHMLCWDVRHTY
S.moellendorffi 220 AGSYDRTIAVYNEGNMELLYVLQGHEGGLTQVQFSKDGNYLYSGARKDPSICCWDVRYTT
P.patens 227 AGSYNKTTALYSEHNAELLFVLHGQEGGVTQVLFSKDGNYLYTGGRKDPNILCWDVRNTA
Z.mays_1 256 VGSYSQTTAVYAENNMEPLYVLHGQLGGVTQVLFSKDGNYLYTGGRKDPYILCWDIRNTV
Z.mays_2 239 VGSYSQTTAVYAENNMEPLYVLHGQLGGVTQVLFSKDGNYLYTGGRKDPYILCWDIRNTV
O.sativa 130 SGSFDETVRVWEVRSGRCLRVLPAHSEPVTSVDFNRDGAMIVSGSYD-GLCRIWDSATGH
A.trichopoda 238 AGSYSQTTAIYVEDNMELLYLLHGQVGGVTQVQFSKDGNYLYTGGRKDPYILCWDVRNTV
G.max 259 LGSYNQTTAIYREDNMELLYFLHGQEGGITHVQFSRDGNYLYTGGRKDPYILCWDVRKSV
S.lycopersicum 247 TGSYSQTTAIHREDNMELLYVLHGQEGGVTHVQFSKDGNYLYTGGRKDPYILCWDIRKTA
V.vinifera 239 TGSYSRTTAIYREDNMELLYILHGQEGGVTNVQFSKDGNFLYTGGRKDPYILCWDIRNTV
G.raimondii 237 TGSYSQTTAIYREDNMELLYVLHGQEGGITHVQFSKDGNYLYTGARKDPYIMCWDIRKAV
P.trichocarpa 236 TGSYSQTTAIYREDNMELLYVLHGQEGGITHVQFSKDGNYLYTGGRKDPYILCWDIRKAV

A.thaliana 290 EI---VYKLYRATENTNQRV--
C.reinhardtii 335 AA---LYSMERPTAHTNQRV--
S.moellendorffi 280 SL---VYKLERAVTNTNQRI--
P.patens 287 AI---VYKLPRVTSDTNQRI--
Z.mays_1 316 DI---VYKLYRSADTTNQRI--
Z.mays_2 299 DI---VYKLYRSADTTNQRI--
O.sativa 189 CIKTLIDDESPPVSFAKFSPNGKFVLAATLDSKLRLWNFSAGKFLKTYTGHVNTKYCIPA
A.trichopoda 298 DI---LYKLYRSSENTNQRI--
G.max 319 DC---VYKLYRSSENTNQRI--
S.lycopersicum 307 EI---VYKMYRSSETTNQRI--
V.vinifera 299 NT---VYKLYRSTETTNQRI--
G.raimondii 297 EV---VYKLYRSSESTNQRI--
P.trichocarpa 296 EV---VYKLYRSSEYTNQRI--

A.thaliana 307 FFDIEPCGRHLGTGGQ-DGLVHMYDLQTGNWVSGYQAASDTVNAFSFHPYLPMAATSSGH
C.reinhardtii 352 QFDIEPAGRHLLTGGC-DGAVLVYDLSSGQLVDRRQVAADTVNGVGFHPTLGLLATASGE
S.moellendorffi 297 AFDIEPYAQHLGTGGEQGGDIRFYDLRTGEFTASIHAASDTINGFSFHPTLPLAASTSGA
P.patens 304 AFDIEPCGRHLGTGGQ-DGYVHIYDLQTGDWANSFQAAADTVNAFSFHPTLPVGVTSSGH
Z.mays_1 333 HFDIEPCGRHLATGGQ-DGMVHVYDLQGGQWVTGYQAAADTVNGFSFHPYLPFAVTSSGH
Z.mays_2 316 HFDIEPCGRHLATGGQ-DGMVHVYDLQGGQWVTGYQAAADTVNGFSFHPYLPFAVTSSGH
O.sativa 249 AFSI-TNGKYIVSGSE-DKCVYIWDLQSRKILQKLEGHTDTVIAVSCHPNENMIASGGLD
A.trichopoda 315 SFDIEPGGRHLGTGGQ-DGQVHIYDLQTGNWTASFQAASDTLNGFCFHPYYPMAASSSGH
G.max 336 LFDIDPSGKYLGTGGQ-DGLVHIYNLQTGQWVSSFEAALDTVNGFSFHPFLPHAVSSSGH
S.lycopersicum 324 YFDIEPRGQHLGSGGQ-DGSVHIYNLQTGQWVSSFRAASDTVNGFSFHPFLPMAASSSGQ
V.vinifera 316 FFDIEPGGRHLGTGGQ-DGLVHIYDLQTGQWISCFQAASDTINGFSFHPFLPMAASSSGH
G.raimondii 314 AFDIECSGRHLGTGGQ-DGLVHIYDLQTGQWVSGFQAAADTVNGFSFHPFLPMAASSSGH
P.trichocarpa 313 FFDIEPLGRHLATGGQ-DGLVHIYDLQNGQWISGFQAATDTVNGFAFHPFLPMAVSSSGH

```
A.thaliana         366 RRFAIPDDDDGEDKNELQLKADE--NCVSLWSFYVEDNTYDENNGVASESHLQNVT--EE
C.reinhardtii      411 RGHNVLGHVRAA*-----------------------------------------------
S.moellendorffi    357 RRFSTSEDKDDDEEPPQILTGNYFQNHLSIALLMV*------------------------
P.patens           363 RRFSTDYDDEGISKETSCI-AQE--NCASVWLFPCTWQSSIDPSSTVETTELENLQGESL
Z.mays_1           392 RRFGMQDEFE----DELNLAGDE--NSCSVWMFSSSQEA*--------------------
Z.mays_2           375 RRFGMQDEFE----DELNLAGDE--NSCSVWMFSSSQEA*--------------------
O.sativa           307 GD-----------------------KTVKVWVQKEEDQMEV*------------------
A.trichopoda       374 RRFGVPDSE-----EDLCLSVEE--NCVSVWNFSCTWITNNSAYKDDEENGIPVEHEDLD
G.max              395 RRFVIPDDG-N---EEFCLTGRE--NCLSMWTFCCDSMMETDLKNDGS-FNNQSESRCLD
S.lycopersicum     383 RRFGVIDDS-H---EDMLLTGDE--NCVSVWSFAYSASVENDVSPISGVSNGKSELDEDL
V.vinifera         375 RRFGIPESNED---LSLGISGDE--NCASVWSFSYLSS*---------------------
G.raimondii        373 RRFQILEDD-N---EDLQLRGEE--NCASVWSFSYDVTAGEQP*----------------
P.trichocarpa      372 RRFKVPDGC-D---ENLPLTGYE--NCASVWNFSCAASMEEDGINIDGGDFTQSE---NE

A.thaliana         422 IVLT*--------
C.reinhardtii          -------------
S.moellendorffi        -------------
P.patens           420 GTW-EENV*----
Z.mays_1               -------------
Z.mays_2               -------------
O.sativa               -------------
A.trichopoda       427 SIDQNPDVLEAH*
G.max              448 *------------
S.lycopersicum     437 HVLQEP*------
V.vinifera             -------------
G.raimondii            -------------
P.trichocarpa      423 ILHQDP*------
```

(B)SMU2_model species

```
A.thaliana        1 ------------------------------------------------------------
S.pombe           1 ------------------------------MNQDDFRKLLATPKAETSQLKNFSSNKSQR
C.elegans         1 -----------------------MADNPTNLRNADFRKLLTSARSDRPAVSAFAKPADPK
D.melanogaster    1 -------------MSDAH----LEAPPSVRLTNDDFRKLLATPRAPPPGSGTS-------
D.rerio           1 MPERETDVYSNPLAPDGHEVEDHRSALQSKLTNDDFRKLLMTPRATPSSAPPS-------
M.musculus        1 MPERDSEPFSNPLAPDGHDVDDPHSFHQSKLTNEDFRKLLMTPRAAPTSAPPS-------
H.sapiens         1 MPERDSEPFSNPLAPDGHDVDDPHSFHQSKLTNEDFRKLLMTPRAAPTSAPPS-------

A.thaliana        1 ---MKPSKSHH------------------------------------------KEKTARR
S.pombe          31 L-------VFGRKHKTAKLEAPRALIKRKQLSHST-------------------------
C.elegans        38 TGDDKPASFKHKHLKPAKFKKPQAAAHGK-----AK--------------KE---KTEAD
D.melanogaster   37 ------STSAGGSLATATFATPSTAPGTTGLSGEKKKGQSSSERNDLRRKKKNFYAALKK
D.rerio          54 ------KTRHHE------------------MP---REYNEDEDPAARRRKKKSYYAKLRQ
M.musculus       54 ------KSRHHE------------------MP---REYNEDEDPAARRRKKKSYYAKLRQ
H.sapiens        54 ------KSRHHE------------------MP---REYNEDEDPAARRRKKKSYYAKLRQ

A.thaliana       16 REEKLEESDNPKYRDRAKERRENQNPD-YDPSEL-------SSFHAVAPPGAVDIRAADA
S.pombe          59 ---------SSDITRHSNAKNSGKDTQFYEEPSSK----QDIELHKLHEKL---RNGQIT
C.elegans        76 EDEAALKNILKNYRDRAAERRKQGDEK-E------DPSKLTAAYRAVPGDARSAQDQADL
D.melanogaster   91 QEDVKLQELSEKYRDRARERRDGANPD-YVNVSTPGHGSSTNAYRAVAPDMKSGIDAQER
D.rerio          87 QEMERERELAEKYRDRARERRDGVNKD-YEETEL---ISTTANYRAVGPTAEADKSAAEK
M.musculus       87 QEIERERELAEKYRDRAKERRDGVNKD-YEETEL---ISTTANYRAVGPTAEADKSAAEK
H.sapiens        87 QEIERERELAEKYRDRAKERRDGVNKD-YEETEL---ISTTANYRAVGPTAEADKSAAEK

A.thaliana       68 LKISIENSKYLGGDVEHTHLVKGLDYALLNKVRSEIVKKPDGE--------DGDGGKTSA
S.pombe         103 TKEYSEKSKELGGDLNTTHLVRGLDRKLLEKVRSNELALDDSLLSSSEKEVDEEADKLL-
C.elegans       129 RKQAILESKYLGGDLEHTHLVKGLDYSLLNKVRSEIDKSDDDD--------DDDIDTAFD
D.melanogaster  150 RRRIIQESKFLGGDIQHTHLVKGLDYALLQKVRSELHSKEAEE--------EEIAAAVAR
D.rerio         143 RRQLIQESKFLGGDMEHTHLVKGLDFALLQKVRAEITSKEREE--------EDMIEKVQK
M.musculus      143 RRQLIQESKFLGGDMEHTHLVKGLDFALLQKVRAEIASKEKEE--------EELMEKPQK
H.sapiens       143 RRQLIQESKFLGGDMEHTHLVKGLDFALLQKVRAEIASKEKEE--------EELMEKPQK

A.thaliana      120 P------------------KEDQRVTFRTIAAKSVYQWIV-KPQTIIKSNEMFLPGRMTF
S.pombe         162 EKVAEESS--HPESVSIL---------EEK-----------------KKIPLYPNGQPKY
C.elegans       181 EKVTSSSSSSKPSEASLLAQELAQSHSENRMVRSLHRVLF--KNEVPLHNQLFAKGRMAY
D.melanogaster  202 EKLAEAAAAAEQLEAERR-ESEDINAINGALARNIYNLVQARRSKEVPRNELFAPGRMAY
D.rerio         195 EVKKDE-------------DPEQKIEFKTRMGRNIYRNVF--KGRQLERNELFLPGRMAY
M.musculus      195 ETKKDE-------------DPENKIEFKTRLGRNVYRMLF--KSKSYERNELFLPGRMAY
H.sapiens       195 ETKKDE-------------DPENKIEFKTRLGRNVYRMLF--KSKAYERNELFLPGRMTY

A.thaliana      161 VYDMEGGY-THDIPTTLYRSKADCPVPEEF-VTVNVDGSVLDRIAKIMSYLRLGSSGKVL
S.pombe         194 RKILENGKK--------------------VKYLLDENGEILKRLVKKEKKL---------
C.elegans       239 VVELEDE--ETDIPTTLLRSLHDLPRAESA-QSIQANNLIILKLSHVLSHLRAEPKKKKK
D.melanogaster  261 VIDLDDELGESDIPTTLKRSKFEVPVSQEDVATLTTNDIVINKLSQILSYLRAGGRNKKN
D.rerio         240 VVDLEDEYADTDIPTTLIRSKADCPTME-AQTTLTTNDIVISKLTQILSYLRQGTRNKKL
M.musculus      240 VVDLDDEYADTDIPTTLIRSKADCPTME-AQTTLTTNDIVISKLTQILSYLRQGTRNKKL
H.sapiens       240 VVDLDDEYADTDIPTTLIRSKADCPTME-AQTTLTTNDIVISKLTQILSYLRQGTRNKKL

A.thaliana      219 KKKKKEKDGKGKMSTIANDYDEDDNKSKIENGSSVNISD--REVLPPPPPLPPGINHLDL
S.pombe         225 KNDN-ER--------LEN-------EHRTE-KLNVNANSLGKSFVKHDIPLPPV------
C.elegans       296 EE-------------FRVQL-----GSRDA----------PGAAAAAPGA--KG------
D.melanogaster  321 KKRDKDK-----PLFYEKEVENLR-GSSSNGGSSRHATSSSSNMGGKSAP--LG------
D.rerio         299 KKKDKGR-----LDE------------------------------KKAPE--AD------
M.musculus      299 KKKDKGK-----LEE------------------------------KKPPE--AD------
H.sapiens       299 KKKDKGK-----LEE------------------------------KKPPE--AD------
```

```
A.thaliana        277 STKQEEPPVARTDDDDIFVGEGVDYTVPGKDVTQSP-I-----------------------
S.pombe           262 -----------DLKLDIFEEVG-EYD-PFHENDKEPAE-----------------------
C.elegans         320 --------------DSIYDDLD-DY-VPSRKSRDSRDAGRRGSRRDRSRDRSRDRDRDRD
D.melanogaster    367 --------------DNIYDDIG-DY-QPSATRPERNRQ----------------------
D.rerio           316 --------------LSIFDDIG-DY-IPSTSKGFRDKEKERYRERERERKDRER-------
M.musculus        316 --------------MNIFEDIG-DY-VPSTTKTPRDKERERYRERERDRERDRDRERDRE
H.sapiens         316 --------------MNIFEDIG-DY-VPSTTKTPRDKERERYRERERDRERDRDRDRERE
```

```
A.thaliana      314 --------SEDMEESPRDKEKVSYFDEPAYGPVQEKVPY----FAEPAYGPVQPSAGQEW
S.pombe  287 -----------------LKAKDAFQLKG---HHELDAPYHKKVFDTNQYSDLKPTNF---
C.elegans       364 RD-----------------NRDRYFEKSANSRREE------------------------E
D.melanogaster     389 ---------------EQGKSAPSYFGDGPPEASEE------------------------P
D.rerio        353 ---ERERERERERDRDEEKRRHSYFEKP--RADEE------------------------V
M.musculus      360 RDRERERERDREREREEEKKRHSYFEKP--KVDDE------------------------P
H.sapiens    360 RERDRERERERDREREEEKKRHSYFEKP--KVDDE------------------------P

A.thaliana      362 QDMSAYGAMQTQGLAPGYPGEWQEYQYAEQTGYQEQYLQPGMEGYEVQPETDVLLDPQLM
S.pombe  324 -----------------------------------------------------------M
C.elegans       383 QNRREQQ--------------------------------RERE----------------R
D.melanogaster     410 II---------------------------------------------------------S
D.rerio        384 IDIDDKG--------------------------------PGSVK------------DQIK
M.musculus      394 MDV-DKG--------------------------------PGSAK------------ELIK
H.sapiens    394 MDV-DKG--------------------------------PGSTK------------ELIK

A.thaliana      422 SQEEKDRGLGSVFKRDDQRLQQLRESDAREKDPTFVSESYSECYPGYQE-YNHEIVGSDE
S.pombe  325 SQIHRLA---KVQERKEE------EERKKGKDGQIVDAGFGLVLSKDDTADIHELGESDD
C.elegans       395 AEQERR---RE---REKEREQEKAKEREKKRKELEESSGYDECYPGGLV-EMGGAWDSDE
D.melanogaster     413 SI------------------PPPPKISKAMAARFAEPEGYAECYPGLEE-MNDAIDDSDD
D.rerio        400 LINEKFAGAAH---QLGPEAGSRRDEKKHLGDFFGMSNSYAECYPAT---MDDLAVDSDE
M.musculus      409 SINEKFAGSAG---WEGTESLKKPEDKKQLGDFFGMSNSYAECYPAT---MDDMAVDSDE
H.sapiens    409 SINEKFAGSAG---WEGTESLKKPEDKKQLGDFFGMSNSYAECYPAT---MDDMAVDSDE

A.thaliana      481 EPDLSKMDMGGKAKGGLHRWDFETEEEWEKYNEQKEAMPKAAFQFGVKMQDGRKTRKQNR
S.pombe  376 DDNVKRRKT---KG----------------------------------------------
C.elegans       448 EADYSKMDAGPKKNQAVNRWDFDTEEEYASYMEGREALPKAAYQYGVKNGEGGRKNKKQS
D.melanogaster     454 EVDYTKMDLGNKKG-PIGRWDFDTQEEYSDYMSTKEALPKAAFQYGVKMQDGRKTRKNKT
D.rerio        454 EVDYSKMDQGNKKG-PLGRWDFDTQEEYSDYMNNKEALPKAAFQYGIKMSEGRKTRRFK-
M.musculus      463 EVDYSKMDQGNKKG-PLGRWDFDTQEEYSEYMNNKEALPKAAFQYGIKMSEGRKTRRFK-
H.sapiens    463 EVDYSKMDQGNKKG-PLGRWDFDTQEEYSEYMNNKEALPKAAFQYGIKMSEGRKTRRFK-

A.thaliana      541 D--RDQKLNNELHQINKILTRKKMEKEGGD-VASLDAAEAQTPKRSKH
S.pombe     ------------------------------------------------
C.elegans       508 AVSDAKRLDRELNEINKIMDKRKAGGDGAGGGGDYKKPKY--------
D.melanogaster     513 EKNEKAELDREWQKIQTIIQKRKLPKDGGGGAGGDEPDYKSAKY---
D.rerio        512 ETNEKAELDRQWKKISAIIEKRKKMEADG---VDIKRPKY--------
M.musculus      521 ETNDKAELDRQWKKISAIIEKRKRMEADG---VEVKRPKY--------
H.sapiens    521 ETNDKAELDRQWKKISAIIEKRKKMEADG---VEVKRPKY--------
```

(B)SMU2_plant species

```
A.thaliana         1 ------------------------------------------------------------
C.reinhardtii      1 MNNDDFRRLLATPRA----------------------ERFGGETPA-----AGKSGQAQA
S.moellendorffi    1 ------------------------------------------------------------
P.patens           1 ------------------------------------------------------------
O.sativa_1         1 ------------------------------------------------------------
O.sativa_2         1 ------------------------------------------------------------
Z.mays_1           1 ----RLPPLLGLPAAQPGLPPPLXFPPHRHPTPPQPAPCYQATLPRSSSAAAGIQRRERE
Z.mays_2           1 ------------------------------------------------------------
Z.mays_3           1 ------------------------------------------------------------
S.lycopersicum     1 ------------------------------------------------------------
A.trichopoda       1 ------------------------------------------------------------
P.trichocarpa      1 ------------------------------------------------------------
G.max_1            1 ------------------------------------------------------------
G.max_2            1 ------------------------------------------------------------
G.raimondii        1 ------------------------------------------------------------
V.vinifera         1 ------------------------------------------------------------
```

```
A.thaliana         1 ---MKPSKSHHKEK---TAR--RREEKLEESDNPKYRDRAKERRENQNPDYDP----SE-
C.reinhardtii     34 QAKAKPTGEYRPKPKPGFKKPTDKKKEDEDEDGPKYRDRAEERRRGKNSDYEDASAVLSA
S.moellendorffi    1 ------------MRVHCLREEKKPTKVDEEDDLPKYRDRAKERREDQNPDYELMAAASG-
P.patens           1 -------------------M--REENAGEDANLPRYRDRAKERREDINPDYDHTS--AE-
O.sativa_1         1 ---MSSKKNYYKE---KMMR--RKEEKKEEPETPRYRDRAKERREDQNPDYEP----TE-
O.sativa_2         1 ---MSSKKNYYKE---KLMR--RKEEKKEEPETPRYRDRAKECREDQNLDYEP----TE-
Z.mays_1          57 GEKMSSKKNYYKE---KLMR--RKEEKKEEPETPRYRDRAKERREDQNPDYEP----TE-
Z.mays_2           1 -----------------------EEKKKEEPETPRYRDRAKERREDQNPDYEP----TE-
Z.mays_3           1 --MLLTVSDWVSSNFSCSPF--SEEKKKEEPETPRYRDRAKERREDQNPDYEP----TE-
S.lycopersicum     1 ---MSSSKRNHKEKI--VRR--NKEEKVEEPELPKYRDRAKERREDQNPDYEL----TE-
A.trichopoda       1 ---MASAKRYHKEKM--ARR---KEEKQDEPELPKYRDRAKERREDQNPDYEP----TE-
P.trichocarpa      1 ---MTSSKKHYKEKI--ARR---KEEKPEESETPKYRDRAKERREDQNPDYEP----TE-
G.max_1            1 --MTASNKKNPKEKP--IRR---KEEKPEEPEVPKYRDRAKERREDQNPDYEQ----TE-
G.max_2            1 --MTASNKKNHKEKP--IRR---KEEKPEEPEVPKYRDRAKERREDQNPDYEQ----TE-
G.raimondii        1 ---MTSSKKYYKEKI--ARR---KEEKAEEPEQPKYRDRAKERREDQNPDYEP----TE-
V.vinifera         1 ---MASSKRNHKEKI--IRR---KEEKPEEPELPKYRDRAKERREDQNPDYEP----TE-
```

```
A.thaliana        48 LSSFH------AVAPPGAVDIRAA-DALKISIENSKYLGG--------------------
C.reinhardtii     94 LGGSGGGGAGGGGPL-GPDIGQV-DLSKLSEEETKYLGG--------------------
S.moellendorffi   48 ISTLH------AVAPPGAVDLQAAEIQKKISIANSKYLGG--------------------
P.patens          37 LGSLH------ALGPPGTADMRLT-DAHRISIENSKFLGG--------------------
O.sativa_1        48 LGSFH------AVAPPGA-DLRLA-DAHKISIEKSKYLGG--------------------
O.sativa_2        48 LGSFH------AVAPPGA-DLRLA-DVHKISIEKSKYLGG--------------------
Z.mays_1         107 LGSFH------AVAPPGN-DLRLA-DAHKISIEKSKYLGG--------------------
Z.mays_2          33 LGSFH------AVAPPGS-DLRLA-DAHKISIEKSKYLGG--------------------
Z.mays_3          52 LGSFH------AVAPPGS-DLRLA-DAHKISIEKSKYLGGQGPFQTGITRCGVCQDYIAW
S.lycopersicum    49 FGGFH------AVAPPGNIDLLSA-DAQKLSIEKSKYLGG--------------------
A.trichopoda      48 LGSFH------AVAPPGTVDLRIA-DAHKISIENSKYLGG--------------------
P.trichocarpa     48 LGSFH------AVAPPGTVDIRSVADSNQISIEKSKYLGG--------------------
G.max_1           49 LG-FH------AVAPPGTVDIRSS-DAHKLSIEKSKYLGG--------------------
G.max_2           49 LG-FH------AVAPPGTVDIRSS-DAHKLSIEKSKYLGG--------------------
G.raimondii       48 LGSFH------AVAPPGTVDLRSA-DAHKISIEKSKYLGG--------------------
V.vinifera        48 LGSFH------AVAPPGTVDLRST-DANKISIEHSKYLGG--------------------
```

```
A.thaliana          81 ------------------------------------------------------------
C.reinhardtii      132 ------------------------------------------------------------
S.moellendorffi     82 ------------------------------------------------------------
P.patens            70 ------------------------------------------------------------
O.sativa_1          80 ------------------------------------------------------------
O.sativa_2          80 ------------------------------------------------------------
Z.mays_1           139 ------------------------------------------------------------
Z.mays_2            65 ------------------------------------------------------------
Z.mays_3           104 NNVTILSHLLLLAQAVPHLTVHYLSNWSMGKATRQHLAIGYQTDLIMEYAVHVILCSLIS
S.lycopersicum      82 ------------------------------------------------------------
A.trichopoda        81 ------------------------------------------------------------
P.trichocarpa       82 ------------------------------------------------------------
G.max_1             81 ------------------------------------------------------------
G.max_2             81 ------------------------------------------------------------
G.raimondii         81 ------------------------------------------------------------
V.vinifera          81 ------------------------------------------------------------

A.thaliana          81 -------------------DVEHTHLVKGLDYALLNKVRSEIVKKPDGEDG-DGGK-T--
C.reinhardtii      132 -------------------DMRFTHLVKGLDYALLHKTRSEITKKGDSDEEDEEHGEGHG
S.moellendorffi     82 -------------------DVEHTHLVKGLDYALLNKVRSEIDKKPEDEDEPIKFN-T--
P.patens            70 -------------------DVEHTHLVKGLDFALLHKVRSEIDKEPENEDP---AS-Q--
O.sativa_1          80 -------------------DLEHTHLVKGLDYALLHKVRSEIEKKPEAEDG-KDTQ-S--
O.sativa_2          80 -------------------DLEHTHLVKGLDYALLHKVRSEIEKKPDAEDG-KDTQ-T--
Z.mays_1           139 -------------------DLEHTHLVKGLDYALLHKVRSEIEKKPEAEDG-KDTK-S--
Z.mays_2            65 -------------------DLEHTHLVKGLGYALLHKVRSEIEKKPEAEDG-KDAK-S--
Z.mays_3           164 QKSPVDEQLVVLLYCLFIGDLEHTHLVKGLGYALLHKVRSEIEKKPEAEDG-KDAK-S--
S.lycopersicum      82 -------------------DVEHTHLVKGLDYALLHKVRSEIDKKPETGDEALEGK-A--
A.trichopoda        81 -------------------DLEHTHLVKGLDYALLHKVRSEIDKKPEQEEE-VDGK-T--
P.trichocarpa       82 -------------------DVEHTHLVKGLDYALLNKVRSEIDKKPDSAED-VDGK-S--
G.max_1             81 -------------------DVEHTHLVKGLDYALLNKVRSEIDKKPEAGDD-VEGK-S--
G.max_2             81 -------------------DVEHTHLVKGLDYALLNKVRSEIDKKPETGDD-VEGK-S--
G.raimondii         81 -------------------DVEHTHLVKGLDYALLNKVRSEIDKKPEAGEE-TDGK-S--
V.vinifera          81 -------------------DVEHTHLVKGLDYALLHKVRSEIEKKPEVGDD-ADGK-S--

A.thaliana         118 ---------------SAPKED---QRVTFR-TIAAKSVYQWIVKPQTIIKSNEMFLPGRM
C.reinhardtii      173 ARKEASREAAARAAAKANAAAGRKEEVRFV-TPLARSVFSTLFAPPRASNVREMYGPRRT
S.moellendorffi    120 ---------------PVARVRGLCVVFSFESKVFSQVIYKWLVKPPTLSKTSDMFLPGRM
P.patens           105 ---------------RQQKED---QTMAFR-TATAKMVYQWICKPQTVSKVNSMFLPGRT
O.sativa_1         117 ---------------RSTKED---QAVSFR-TAAAKSVYQWIIKPQSIIKSNEMFLPGRM
O.sativa_2         117 ---------------RSTKED---QAVSFR-TATA-------------------------
Z.mays_1           176 ---------------RAAKED---QAVSFR-TATAKSVYQWIIKPQSIIKENELFLPGRM
Z.mays_2           102 ---------------RATKED---QAVSFR-TATAKSVYQWIIKPQSIIKENELFLPGRM
Z.mays_3           220 ---------------RATKED---QAVSFR-TATAKSVYQWIIKPQSIIKENELFLPGRM
S.lycopersicum     120 ---------------RGVKED---HQLSFR-TATAKSVYQWIIKPQTVIKTNEMFLPGRM
A.trichopoda       118 ---------------RGSKDD---QPLSFR-TATAKSVYEWIVKPQTVVKTNEMFLPGRM
P.trichocarpa      119 ---------------RASKED---QKILFR-TATAKSVYQWIVKPQTIIKTNEMFLPGRM
G.max_1            118 ---------------RSAMED---QQVSIR-TATAKSVYQWIVKPQTISKTNEMFLPGRM
G.max_2            118 ---------------RSAKED---QQVSIR-TATAKSVYQWIVKPQTISKTNEMFLPGRM
G.raimondii        118 ---------------RKSKED---QQLSFR-TATAKSVYQWIVKPQTVMKTNEMFLPGRM
V.vinifera         118 ---------------RVSNED---QPLSFR-TATAKSVYQWIVKPQTVVKSNEMFLPGRM
```

```
A.thaliana          159 TFVYDMEGG--YTHDIPTTLYRSKADCPVPEEFVTVNVDGSVLDRIAKIMSYLRLGSSGK
C.reinhardtii       232 AFVYDFDNEDSPDTDVPTTLRRPKSECPQVAETLFAAVDNAVLERIAKIMSYVRTTADGK
S.moellendorffi     165 AYAFDMEE---MSHHIPTTVYRSKDDCPTPEEFVTVGVDVAVLDRIAKIMAYLKLGASGK
P.patens            146 AFVFDMDGE--EAHDIPTTVHRSKADCPPPQETVTVGVDGTVLERIAKIMTYLRLGAG-K
O.sativa_1          158 AFIYNMEDG--LTNDIPTTLHRSKADCSVPEEMVTVSVDGSVLDRIAKIMSYLRLGSSGK
O.sativa_2          133 -----KEDG--LTNDIPTTLHRSKADCAVPEEMVTVSVDGSVLDRIAKIMSYLRLGSSGK
Z.mays_1            217 SFIYNMEEG--VTNDIPTTLHRSKADCPVPEEMVTVSVDGSVLERIAKIMTYLRLGSSGK
Z.mays_2            143 SFIYNLEEV--VTNDIPTTLHRSKADCPVQEEMVTVSVDGSVLDRIAKIMTYLRLGSSGK
Z.mays_3            261 SFIYNLEEV--VTNDIPTTLHRSKADCPVQEEMVTVSVDGSVLDRIAKIMTYLRLGSSGK
S.lycopersicum      161 AFIFNMDSG--YSNDIPTTLHRSKADCPVLEEMVTVSVDGSVLDRIAKIMSYLRLGSSGK
A.trichopoda        159 AFVFDMEED--YAHDIPTTVHRSKADCPVPQEMVTVSVDGSVLDRIAKIMSYLRLGSSGK
P.trichocarpa       160 SFIFNMEGG--YSHDIPTTLHRSKADCPVPEEMVTVSVDGSVLDRIAKIMSYLRLGSSGK
G.max_1             159 TFIYNMEGG--YHHDIPTTLHRSKADCPVPEEMVTVNVDGSVLDRIAKIMSYLRLGSSGK
G.max_2             159 TFIYNMEGG--YHHDIPTTLHRSKADCPVPEEMVTVNVDGSVLDRIAKIMSYLRLGSSGK
G.raimondii         159 AFIFNMEGG--YSNDIPTTLHRSKADCPVPDEMVTVNVDGSVLDRIAKILSYLRLGSSGK
V.vinifera          159 AFIFSMEGG--FSSDIPTTLHRSKADCPVPEEMVTVGVDGSVLDRIAKIMSYLRLGSSGK

A.thaliana          217 VLKKKKKEK-DGKGKMSTIANDYDED-DNKSKIENGSSVNISDREVLPPPPPLPPGINHL
C.reinhardtii       292 KLKKKDRDA---------LLGIKSEADAA------------EKDLAGVSPGAP--------
S.moellendorffi     222 VREFVLDFL---------RLSLTFVLQTSKKKRRDKSDTKETHDDEVVKDRPS--------
P.patens            203 PLKKKKKDK-ESRAKSVSLGGETPLH-DGERGATNGTANRSHEWENLPPPPPLPPRRFGE
O.sativa_1          216 VLKKKKKER-DTKGKNSLASGDYDEV-ARPGQTNGSALKHQFE--KDMPPPP-PPRNNNL
O.sativa_2          186 VLKKKKKER-DTKGNNSLASGDYDEV-ARPGQT-GSSLKHQFQ--KDMPPPP-PPRNNNL
Z.mays_1            275 VLKKKKKER-DIKGKSNLASGDYGES-VKPSQTNGSTLKHQSD--MPPPPAP-PPRNNNF
Z.mays_2            201 VLKKKKKER-DIKGKNNLASGDCDES-VKPSKINGSTQKHQSD--MPPPPAR-PPQNNNF
Z.mays_3            319 VLKKKKKER-DIKGKNNLASGDCDES-VKPSKINGSTQKHQSD--MPPPPAR-PPQNNNF
S.lycopersicum      219 VLKKKKKEK-DSKGKT-VISNGYDEV-LK-----SDASKSQIDKETVHPSAQ-LPKKNHS
A.trichopoda        217 VLKKKKKDRSEVKGKISAVGHEDDED-EKALRPADGIAKHQVYKEMGVP--Q-PSNGNHR
P.trichocarpa       218 VLKKKKKDK-DAKGKISVVGNEYDEH-DKPSKPSGGMLNNKTEREILPPPPP-PPKNNLA
G.max_1             217 ILKKKRKEK-DAKGKILAVGNGFDKE-DKPSKVEGGAK-NQTEKEIILPPPP-PIKKNPL
G.max_2             217 ILKKKKKEK-DAKGKILAVGNGFDKE-DKPSKVEGGAK-NQTEKEII--PPP-PIKKNPL
G.raimondii         217 VLKKKKKER-DAKGKISSLGNEYYEE-EKLSKPNDGVSNGRTEKEILPPTPP-PPRKNHL
V.vinifera          217 VLKKKKKER-DVKGKISTVGNEFDEE-KKPSKLDGGMSKNQTERESLPP-PL-PPRKNYV

A.thaliana          275 DL------------STKQEEPPVARTDDDDIFVGEGVDYTVPGKDVTQSPISEDMEESPR
C.reinhardtii       324 -----------ASAAAAEPAKPAAPVDDDEDIFGDAGTDYQPTTKGAKAK----DAAAPPG
S.moellendorffi     266 ----------DTDDAAVEEEAPKPRVAEDDIFADAGTDYVVT---IDGSPRSEDMEESPR
P.patens            261 MRESISIDQAAVSNLPLPPPPPHPPSRDDDIFEGVGTDYVP-TVRDNDSPKSEDMEESPG
O.sativa_1          271 SK-------------NEKPSAPVARADEDDIFVGDGVYYSVPNKEMSQSPVSEDMDESPH
O.sativa_2          240 SK-------------NEEQSIPVARADEDDIFVGDGVDYSVPNKEMSHSPVSEDMDESPH
Z.mays_1            330 NG-------------KEKQPVPVSREDDDDIFVGDGVDYLVPNKEMSQSPVS-DMDESPH
Z.mays_2            256 TG-------------KEKQPVPVARADDDDIFVGDGVDYTVPNEEMSQSPVS-DMDESPH
Z.mays_3            374 TG-------------KEKQPVPVARADDDDIFVGDGVDYTVPNEEMSQSPVS-DMDESPH
S.lycopersicum      270 ER-------------REVQGPVVARPEEEDIFIGEGVDYSVPAGDMGQSPVSEDMEESPR
A.trichopoda        273 DE-------------KNKQGNPVNRAGADDIFVGDGTDYTVGAKDLSQSPISEDMEESPR
P.trichocarpa       275 DS-------------FEKQQPAVAREDENDIFVGHGIDYEVPGKDMSQSPLSEDMEESPR
G.max_1             273 HS-------------IEKQGPAVARAEDDDIFVGEGVDYDIPGKDLSQSPVSEDMEESPR
G.max_2             271 HS-------------REKQGPAVARAEDDDIFVGEGVHYDVPGKDLSQSPVSEDMEESPR
G.raimondii         274 DS-------------REKQGPTIARAEEDDIFVGEGVDYNSPEKDAVPSPLSEDMEESPR
V.vinifera          273 DS-------------REKHGPSVARSEQDDIFVGDGVEYDIPSKDMSQSPVSEDMEESPR
```

```
A.thaliana        323 DKEK--VSYFDEPAY-GPVQEKVPY---------------------------FAEPAYGP
C.reinhardtii     370 A-RRPDGSYFDTKDTMDDIALPGTRLRRPGGGADQGADGGGGGGGGGDDDMDLEEGEAAP
S.moellendorffi   313 RDGNGGASYFHEE-----------------------------------------------
P.patens          320 RDNE--RPYFG--------------------------------------------EHYGP
O.sativa_1        318 NHQK--QSYFTE-----------------------------------------EKPIYGP
O.sativa_2        287 NHQK--QSYFTE-----------------------------------------EKPVYGP
Z.mays_1          376 NHQK--QSNFT-------------------------------------------EPLYGP
Z.mays_2          302 NHQK--QSNFI-------------------------------------------EPMYGP
Z.mays_3          420 NHQK--QSNFI-------------------------------------------EPMYGP
S.lycopersicum    317 NKER--TSYFS-------------------------------------------EPAYGP
A.trichopoda      320 NRER--PSYFS-------------------------------------------DPVYGP
P.trichocarpa     322 NKER--LSYFS-------------------------------------------EPVYGP
G.max_1           320 NKEK--PSYFT-------------------------------------------EPTYGP
G.max_2           318 NKEK--PSYVT-------------------------------------------EPTYGP
G.raimondii       321 HKER--VSYFP-------------------------------------------EPAYGP
V.vinifera        320 NKER--ISYLS-------------------------------------------EPAYGP

A.thaliana        353 VQPS--AGQEWQDMSAYGA-MQTQGLAPGYPGEWQEYQYAE------------QTGYQEQ
C.reinhardtii     429 APAP------GPQPQGYGGQAQVV---Q----GYDAYG---------------NPVYGGQ
S.moellendorffi   326 ----QRSHEDWEQASLLAI----SPAMPQFGSEWQDYLPPPPDDPSYEY-------YANQ
P.patens          334 MPPSQDLNPIWQHQATYDASVQGPAMPY-ASGEWTEYPQPPPEQYAGPWQPEQYPGYMEH
O.sativa_1        335 IPPSD-PAQAWPQPNAYDA-IQAQMVAAGYQGEWSGYQYGE-----------QQMAYPEQ
O.sativa_2        304 IPPSD-PAQAWPQTNGYDA-IQAQMVAAGYQGEWSGYQYGE-----------QQMPYPEQ
Z.mays_1          391 VPPSE-SAQAWQQPNTYDAAVQAQMAAAGYQGDWSSYVYAE-----------QQLGYPEQ
Z.mays_2          317 VPPSE-PAQAWQQPNGYDA-VQAQMAAAGYQGDWSSYVYAE-----------QQLGYPEQ
Z.mays_3          435 VPPSE-PAQAWQQPNGYDA-VQAQMAAAGYQGDWSSYVYAE-----------QQLGYPEQ
S.lycopersicum    332 VPPSE-PSHDWQYTNGYDA-AQAQAVAGVYQPEWQDYQYPE------------QVAYPEQ
A.trichopoda      335 APPTE-LVPDWQQTNGYDT-VHGPVMPTTYQGEWQDYQYAE------------QMAYPDQ
P.trichocarpa     337 VPPSE-LSHEWQDPNGYDA-VHAQALSADYQGEWQNYQYAE------------QFAYPEQ
G.max_1           335 VQPSM-VPQGWQETNGYDV-MQTQALAAGYQGEWQEYQYAE------------QLAYPDQ
G.max_2           333 VPPSM-VPQGWQETNGYDV-MQTQAFDAGYQGEWQEYQYAE------------QLAYPDQ
G.raimondii       336 VSPS--AAQEWQELNGYDA-LQTQALAGGYQGEWQDYQYAD------------QLAYPEQ
V.vinifera        335 VPPS--EPQEWQQTNGYDA-MQAQALAAGYQGDWQEYQYAE------------QMAYPEQ

A.thaliana        398 YL---------QPGMEGYEVQPETDVLLDPQLMSQEEKDRGLGSVFKRDDQRLQQLRESD
C.reinhardtii     461 YGAGGAYGQAPQQAANAYG------TAEEPRWRASRTRDAKVKAVL--------------
S.moellendorffi   371 LSV--------EGNASVPAAAIAPVAPKDPLCMTQEEKDRGMSSVFKRDDQRLRQRRELD
P.patens          393 YP---------T------AAVANPEVQGDPHLMTQEEKDRGLGSVFKRDDQRLQQRRERD
O.sativa_1        382 YM---------QQSAQDCDVLADPNITQDPRLMTQADKDRGLGSVFKRDDERLKQLREKD
O.sativa_2        351 YM---------QQSTQDYDVLADPNIAQDPRLMTQADKDRGLGSVFKRDDERLKQLREKD
Z.mays_1          439 YV---------QQSTQEYDVLADPSISQDPRFMTQADKDRGLGSVFKRDDQRLNQLREKD
Z.mays_2          364 YV---------QQSTQEYDVLADPSISQDPRFMTQADKDRGLGSVFKRDDQRLNQLREKD
Z.mays_3          482 YV---------QQSTQEYDVLADPSISQDPRFMTQADKDRGLGSVFKRDDQRLNQLREKD
S.lycopersicum    378 YL---------QQN---YDMQADVDGLQDPQFMTQEEKDRGLGSVFKRDDQRLLQLRERD
A.trichopoda      381 YI---------QHNVNDYEPEAGVTVPLDPRFMTQEDKDRGLGSVFKRDDQRLQQLREKD
P.trichocarpa     383 YT---------QQTMQAYDMQAASSIQPDPRFMTQEEKDRGLGSVFKRDDQRLQQLREKD
G.max_1           381 YL---------QQNMQAYDEQADLNLPLDPRFMTQEEKDRGLGSVFKRDDQRLQQLREKD
G.max_2           379 YL---------QQNMQPYDVQAGLNLPQDPRFMSQEEKDRGLGSVFKRDDQRLQQLRERD
G.raimondii       381 YL---------QASMQGYEVQAGSNIPQDPRYMTQEEKDRGLGSVFKRDDQRLQQLREKD
V.vinifera        380 YL---------QQNMQTYDVQAGMGIPQDPRFMTQEEKDRGLGSVFKRDDQRLQQLREKD
```

```
A.thaliana         449 AREKDPTFVSESYSECYPGYQEYNHEIVGSDEEPDLSK-MDMGGK-AKGGLHRWDFETEE
C.reinhardtii      501 ---GEADGEDDAYAEYYPGMAGYAGALADSDDEGDKAGTADMDSKTANKSKTRADFTTDE
S.moellendorffi    423 SREKDPNFVSESYSECYPGYQEYNREIVDSDEDADLTK-MDVGGR-AKGRLHRWDFETEE
P.patens           438 VREKDPNFISESYSECYPGYQEYNREIVGSDDDEDLTK-MDMGGR-AKGRLHRWDFESED
O.sativa_1         433 AREKDPNFISDSYSECYPGYQEYNHEIAGSDEEDDLSK-MDMGGR-AKGRLHRWDFETEE
O.sativa_2         402 SREKDPNFISDSYSECYPGYQEYNHEIAGSDEEDDLSK-MDMGGR-AKGRLHRWDFETEE
Z.mays_1           490 ARERDPNFISDSYSECYPGYQEYNNEIAGSDDEDDLSK-MDMGGR-AKGRLHRWDFETEE
Z.mays_2           415 ERERDPNFISDSYSECYPGYQEYNNEIAGSDDEDDLSK-MDMGGR-AKGRLHRWDFETEE
Z.mays_3           533 ERERDPNFISDSYSECYPGYQEYNNEIAGSDDEDDLSK-MDMGGR-AKGRLHRWDFETEE
S.lycopersicum     426 AREKDPNFISESYSECYPGYQEYNREVVDSDDEADLSK-MDMGGR-AKGRLHRWDFETEE
A.trichopoda       432 AREKDPNFISESYSECYPGYQEYNREVVFSDDEDDLSK-MDMGGR-AKGRLHRWDFETEE
P.trichocarpa      434 AREKDPNFISDSYSECYPAYQHFTREVVDSDDEDDLSK-MDMGGR-AKGRLHRWDFDTED
G.max_1            432 AREKDPNFISESYSECYPGYQEYNREIVDSDDEDDLSK-MDMGGR-AKGRLHRWDFETEE
G.max_2            430 AREKDPNFISESYSECYPGYQEYNREIVDSDDEDDLSK-MDMGGR-AKGRLHRWDFETEE
G.raimondii        432 AREKDPNFISESYSECYPGYQEYNREIVDSDDEDDLSK-MDMGGR-AKGRLHRWDFETEE
V.vinifera         431 AREKDPNFISESYSECYPGYQEYNREVVDSDDEDDLSK-MDMGGR-AKGRLHRWDFETEE
```

```
A.thaliana         507 EWEKYNEQKEAMPKAAFQFGVKMQDGRKTRKQ--------NRDRDQKLNNELHQINKILT
C.reinhardtii      558 EYTRYKESREHNPKAAYQYGVKRADGRKSGKELEKSAMVEAKQRDQKLDGQLRKIKGIMD
S.moellendorffi    481 EWGKYNDQKEATPKAAYQFGVKMQDGRKTRKQ--------NK--DQKLNNELHKITQIME
P.patens           496 DWAKYNEQKEAMPKAAFQFGVKMQDGRKTRKQ--------NK--DQKLTNELHKINKILD
O.sativa_1         491 EWATYNDQKEAMPKAAFQFGVKMQDGRKTRKQ--------NK--DQKLTNDLHKINKILA
O.sativa_2         460 EWAKYNDQKEAMPKAAFQFGVKMQDGRKTRKQ--------NK--DQKLTNDLHKINKILA
Z.mays_1           548 EWAKYNDQKEAMPKAAFQFGVKMQDGRKTRKQ--------NK--DQKLTNDLHKINKILA
Z.mays_2           473 EWANTMRSI---------------------------------------------------
Z.mays_3           591 EWAKYNDQKEAMPKAAFQFGVKMQDGRKTRKQ--------NK--DQKLTNDLHKINKILA
S.lycopersicum     484 EWATYNEQKEAMPKAAFQFGVKMQDGRKTRKQ--------NK--DQKLTNELHKINKILT
A.trichopoda       490 EWATYNEQKEAMPKAAFQFGVKMQDGRKTRKQ--------NK--DQKLTNELHKINKILA
P.trichocarpa      492 EWAKYNEQKEAMPKAAFQFGVKMQDGRKTRKQ--------NK--DQKLNNELHQINKILA
G.max_1            490 EWATYNEQKEAMPKAAFQFGVKMQDGRKTRKQ--------NK--DQKLNNDLHKINKILA
G.max_2            488 EWATYNEQKEAMPKAAFQFGVKMQDGRKTRKQ--------NK--DQKLNNDLHKINKILA
G.raimondii        490 EWATYNEQKEAMPKAAFQFGVKMQDGRKTRKQ--------NK--DQKLNNELHKINKILA
V.vinifera         489 EWATYNEQKEAMPKAAFQFGVKMQDGRKTRKQ--------NK--DQKLTNELHKINKILA
```

```
A.thaliana         559 RKKMEKEGGDV----------------ASLDAAEAQTPK-RSKH--
C.reinhardtii      618 EKGLDHGTAFDRKPKEDRERGGGGGGGGGGGGETPAHGRKRLRI--
S.moellendorffi    531 KKKREKGGLHE---------------DGDIVAEESSHPGKRIRT--
P.patens           546 RRKTGKGGYE---------------NDDRGGYEEEGTPAKRSRA--
O.sativa_1         541 RKKGDKD-GGH----------------DGGHYDDDMPSGKKQRA--
O.sativa_2         510 RKKGDKD-GGD----------------DGGHYDDDLPSGKKQRA--
Z.mays_1           598 RKKGEKD-GAE----------------DGGHYDDDLPSSKKQRG--
Z.mays_2               ----------------------------------------------
Z.mays_3           641 RKKGEKD-GAE----------------DGGHYDDDLPSSKKQRG--
S.lycopersicum     534 RKKMEKDKGE-------------------ALEDGEIQPGKKQRV--
A.trichopoda       540 RKKAEKGETFD----------------DGGQYDDDAQPGKKLRI--
P.trichocarpa      542 KKKIDKE--NG----------------DGGHYDDDTPPGKKQRQ--
G.max_1            540 RKKMEKDTNGE----------------GGNHYDDEPTPGKKLRI--
G.max_2            538 RKKMEKDTNGE----------------GGHHYDDEPTPGKKLRI--
G.raimondii        540 RKKMEKESGGG----------------EGGSHSDDVQPGKKLRISG
V.vinifera         539 RKKMEKGEMND----------------DGGRYDDDSQPGKKLRI--
```

(C)ZCH1_model species

```
A.thaliana  1   ---------MA--------------QDSEKRFHQIMDKLFTPSKSQLPSSSTSSSVEQQS
H.sapiens   1   MAAPCEGQAFAVGVEKNWGAVVRSPEGTPQKIRQLIDEGIAPEEGGVDAKDTSATS----

A.thaliana  38  RGKKRQNPSSALALVEPKIVLATIDRSSALKVPAGTSPSGLCRPWDRGDLMRRLATFKSM
H.sapiens   57  ---------------------QSVN-----GSPQAEQP--SLESTSKEAFFSRVETFSSL

A.thaliana  98  TWFAKPQVISAVNCARRGWVNDDADSIACESCGAHLYFSAPSSWSKQQVEKAASVFSLKL
H.sapiens   89  KWAGKPFELSPLVCAKYGWVTVECDMLKCSSCQAFLCASLQPAFDFDRYKQRCAELKKAL

A.thaliana  158 ESGHKLLCPWIENSCEETLSEFPLMAPQDLVDRHEERSEALLQL-LALPVISPSAIEYMR
H.sapiens   149 CTAHEKFCFWPDSPSPDRFGMLPLDEPAILVSEFLDRFQSLCHLDLQLPSLRPEDLKTMC

A.thaliana  217 SSDLEE-FLKRPIAPACSDTAAESSQTESLTNHVGASPAQLFYQAQKLISLCGWEPRALP
H.sapiens   209 LTEDKISLLLHLLEDELDHRTDER----KTTIKLG--SDIQVHVTACILSVCGWACSS--

A.thaliana  276 YIVDCKDKLSETARGTETIDLLPETATRELLSISESTPIPNGISGNNENPTLPDTLNSDP
H.sapiens   261 -------PIPGLEGRPERLPLVPESPRRMMTRSQDATFSP-GSEQA--------------

A.thaliana  336 SSVVLDCKLCGACVGLWVFSTVPRPLELCRVTGDTEINIEKHPKGGTLQHQPSSLKFTIA
H.sapiens   299 ---------------------------------------------------------EKS

A.thaliana  396 GGPPATKQNFKATISLPIIGRNLRSRFASYSRDHDHGDVSSIQDQQSRTAENNGDVTQNS
H.sapiens   302 PG--------------PIVSRT-----RSWDS------SSPVDRPE----PEAASPTTRT

A.thaliana  456 NQVMNDIGEKADGGRNSTDVESDIALQNKDKQMMVVRSNLPENNKPRDSTAEKSATSNKQ
H.sapiens   333 RPVTRSMGTGDTPG---LEV---------------PSSPLRKAKRAR-LCSSSSSDTSSR

A.thaliana  516 MEFDPIKQHRHFCPWIWSTG----------------RRGPGWRQTLSALQRHKGSCQTPP
H.sapiens   374 SFFDPTSQHRDWCPWVNITLGKESRENGGTEPDASAPAEPGWKAVLTILLAHKQSSQPAE

A.thaliana  560 SSSSLFKVDDPLTSVRNLFKSPSPKKRKLNGGSSS
H.sapiens   434 TDSMSL--SEKSRKVFRIFRQWESLC----S---C
```

(C)ZCH1_plant species

```
A.thaliana           1 ------------------------------------------------------------
P.patens             1 ------------------------------------------------------------
S.moellendorffi      1 ------------------------------------------------------------
A.trichopoda         1 ------------------------------------------------------------
O.sativa             1 MGEGIGATAGLAACRSAAAITAVRYRLADKDLLVLVTSDKELVHMHDEYDRLRPAILAHP
Z.mays               1 ------------------------------------------------------------
P.trichocarpa        1 ------------------------------------------------------------
G.max_1              1 ------------------------------------------------------------
G.max_2              1 ------------------------------------------------------------
S.lycopersicum       1 ------------------------------------------------------------
V.vinifera           1 ------------------------------------------------------------

A.thaliana           1 ------------------------------------------------------------
P.patens             1 ------------------------------------------------------------
S.moellendorffi      1 ------------------------------------------------------------
A.trichopoda         1 -----------------------------------------------------------M
O.sativa            61 CGRPKSRPVHTDGLESQAHVSEAQQAGTRQRGSFSLSPSHRKRKQRRPLLSSPDDASPLA
Z.mays               1 ------------------------------------------------------------
P.trichocarpa        1 ------------------------------------------------------------
G.max_1              1 ------------------------------------------------------------
G.max_2              1 ------------------------------------------------------------
S.lycopersicum       1 ------------------------------------------------------------
V.vinifera           1 ------------------------------------------------------------

A.thaliana           1 --------------------MAQDSEKRFHQIMDKLFTPSKS-QLPSSSTS------SS-
P.patens             1 ------------------MEVTEVSEKRFERAMERLFSGSST-PAASSARV---------
S.moellendorffi      1 ---------------MTSGDKDDDAEQRIQRAMDRIFSSPIQ-AASSCSSSPPTKSILER
A.trichopoda         2 AEDLFSYK--TCGARPKLGKIAEDVEKRFENVMNKLSTSKCS-FGTTPTSM----AKSA-
O.sativa           121 ADPLVSRHPTMATGGGGGGDIGADSERRLKKAMDKLYHFPKPKAGTGPGSSKPSSAS---
Z.mays               1 ----------MAAGEGGGGDIGADSERRLQKAMDKLYHFPKPKSSSTGGSKPSSST----
P.trichocarpa        1 --------------------MEEDPEKRFHSIMDKLLNAPSS-KSISNPSSPISCGAIQ-
G.max_1              1 --------------------MAQDSEKRFHSIMDKLFHPPKP-PSASSSSG---------
G.max_2              1 --------------------MAQDSEKRFHSIMDKLYHPPKP-PSASSSSG---------
S.lycopersicum       1 --------------------MAEESQKRFQDAMDKIFRTPPKSKLNSSASG---------
V.vinifera           1 --------------------MSEEAEKRLQSVMAKLFHPPKS-KPNSPSDSS-S------

A.thaliana          33 -VEQQSRGKKRQNPSSALALVEPKIVLATIDRSSALKVPAGTSPSGLCRPWDRGDLMRRL
P.patens            33 --PSPVKEKKEDEENTSGRVF------------QTAGVATPLSGSHGCRPWDRGDLLRRL
S.moellendorffi     45 RVVTPLALERSPGEKNAGE---------------KNAGEIASSSTPSCRPWDREDLLRRL
A.trichopoda        54 -SSMP---------ETPLEANP-VGLQ--LHSRSSKDALNGASSPPLCRPWDRGDFLRRL
O.sativa           178 -TSSALSIGRAGK--AAGAGGRRFGMVRGSRLPSQLAAMSAISPPPPCRPWDRADLMRRL
Z.mays              47 --SAPSSGRAVGK--AAAEAARRFGVVRGSRLPHQMLAMAAISPPPPCRPWDRADLMRRL
P.trichocarpa       39 -ISSPSRGKKRDNRESALA----------------LVEHVSSADAPLCRPWDRGDLMRRL
G.max_1             31 -VQLPGSSKKRPYQSGIMELNWRG---DVAEGQQSSSAATMALQGSLCRPWDRGDFMRRL
G.max_2             31 -VQLPGNSKKRPYQSGVMELNRRG---DVAEGQQSSSASATALQGSLCRPWDRGDFTRRL
S.lycopersicum      32 -V-QLSRDKERLDMSSIGKAV----------SKYNLLATKGSGEAPPCRPWDRDDLFTRM
V.vinifera          33 -DGQSSKGKKRPNPMSASAIESKL-RK-------DIQKSTTPVQAPLCRPWDREDLMKRL

A.thaliana          92 ATFKSMTWFAKPQVISAVNCARRGWVNDDADSIACESCGAHLYFSAPSSWSKQQVEKAAS
P.patens            79 ATYKSISWFGKPQVAGPVACARRGWVNVDIDLLACEICGSRLSFPVSSSWSRHQVEQAAL
S.moellendorffi     90 GTFKSVSWFGKPSAAGPVACAQRGWINVDMDLLCCEVCGSRLSFSFPATWSKKEVETAGL
A.trichopoda       101 STFKAVTWFAKPKIVNPVNCARRGWINVERDTIACEACGARLLFCTPSSWTPQQDEKAAA
O.sativa           235 ATFKAMTWFAKPKVISPVNCARRGWINIEPDVITCEACEARLLFSTPSSWAPQQVEKAAA
Z.mays             103 GSFKAMTWFAKPKVISPVNCARRGWTNIEPDVITCEACGARLLFSTPSSWTTQQVEKAAA
P.trichocarpa       82 ATFKSMTWFAKPKVVSAVDCARRGWINLDMDIIACEACGARLLFSTPSSWSQQQVEKAAL
G.max_1             87 ATFKSMSWFAKPKVVSAVNCASRGWINVDIDTISCEACGARLLFSTPASWNQQQVEKAAL
G.max_2             87 ATFKSMSWFAKPKVVGAVNCASRGWINVDIDTIACEACGVRLLFSTPASWNQQQVEKAAL
S.lycopersicum      80 STFKSMTWFAKPQAISAVNCARRGWINVDMDTIACEACGSRMLFTTPPSWAQQQVDKAAL
V.vinifera          84 ATFKSMTWFAKPKVISAINCARRGWVNVEMDIIACEACGARLLFSTPSSWTQQQVEKAAL
```

```
A.thaliana          152 VFSLKLESGHKLLCPWIENSCEETLSEFPLMAPQDLVDRHEERSEALLQLLALPVISPSA
P.patens            139 VFAEKLDTAHKGLCAWKNNPCAETLAHFPPTPVSVLRGAYTDRCEALLQLSALPVISDAA
S.moellendorffi     150 EFSRKLHDGHKTSCPWKGNGCGEDLAAFPPTPAPVLVQAYEARLQSVALLSDLPVISSST
A.trichopoda        161 VFSLKLDHGHKPLCPWKDNICEESLAHFPRVPVPTLIKSYRKRFDALAHLSSLPIVSSSA
O.sativa            295 VFSLKLDNGHKLLCPWIDNICDESLALFPPTPPPVLVENYHEGFSSLLRLSALPRISCSS
Z.mays              163 VFSLKLDSGHKLLCPWIDNICDESLALFPPTPPPVLVGNYYELLSSLLRLLALPRISCSS
P.trichocarpa       142 VFSLKLDNGHKLLCPWIDNACDERLAEFPPTPPQVLVDKFRERSCALLRLLALPLISSSA
G.max_1             147 VFSLKLDNGHKLLCPWIDNACDETLARFPPATPPVLVDNFREHCFALLQLSALPRISPSA
G.max_2             147 VFSLKLDNGHKLLCPWIDNACNETLARFPPATPPVLVDNFRERCFALLQLSALPRISSSA
S.lycopersicum      140 VFSLKLDSGHKLLCPWIDNVCDEKLADFPPTATVMLVDQYKIRHSVLSQLAALPVISPKA
V.vinifera          144 VFSLKLDNGHKLLCPWMDNACDEMLAQFPPATVQDLVDGYKERSSALLQLVALPLISSAA

A.thaliana          212 IEYMR-S--SDLEEFLKRPI-----APACSDTAAESSQTESLTNHVGASPAQLFYQAQKL
P.patens            199 KSLMKLSRGPQVDQLLSELNPPSPGFLV-GNGASSSSTE-----NEIFANSKAYYEAQRM
S.moellendorffi     210 VERMKISRGDQVASLLALPSNDAA--VR-ELEAAQGEAV-----QKLRQTYEAFLQAQKL
A.trichopoda        221 IDCMR-D--PQLDIFLSQSPPLEKSTSEYG--STAPGGVDFV-DHSEAASADLYYKAQKI
O.sativa            355 LESMK-KRSPQLEQFLLKPFSSS-VVLKGGFILTEDSTIKDLDH--TFQDADTYYQALKI
Z.mays              223 LETMK-KRNPQLEQFLSEPFSSS-VVLKGGLMLTEDSTIKDLDD--AFQDADTYYQALKI
P.trichocarpa       202 IEYIR-C--PQLEEFLGQSP-----TLEFGKMSANLSQIQFLGNDCDAGFANLYYEAQKL
G.max_1             207 IDYMQ-SQSTLLEDFLGQSL-----MLEYGNGSAENSGIGDV---SSQEELKLYYQAQKL
G.max_2             207 IDYMQ-SQSPLLEDFLGQSL-----MLEYGNGSAENSGIGDV---SSQEELKLYYQAQKL
S.lycopersicum      200 IDFLR-N--PQLEQFLRESL-----TVEHDESMHTP--QEETRNAPTSVSSLTYYQVQKL
V.vinifera          204 INYMR-S--PQLEHFLRQSA-----VLEFGSVSADSSQTEYLGSECDAVSANLYFQAQKL

A.thaliana          264 ISLCGWEPRALPYIVDCKDKLSETARGTETIDLLPETATRELLSISESTPIPN--GISGN
P.patens            253 IAVCGWEPRLLPYTVDCEDRSGAQSIHE-----QIGTSHGPGPSVTVHMQGGQQS--KVA
S.moellendorffi     262 ISLCGWEVRLLPYAVDSHDSNVDS------------------------------------
A.trichopoda        275 ISLCGWEPRVLPYVIECEDHPSLPSKCPSPSQRSQCLLQVQTPTILLNSKSGDDE-AAVL
O.sativa            411 ISLCGWEPRLLPYAVDCGTKSHSDANSSSTLTQPGLINNSMEDRVVVYAPNEVDGSTV--
Z.mays              279 ISLCGWEPRLLPYAVDCGTEFHSDANSTSKLAQPEQSSKTMEDRVILYSPDGAR---P--
P.trichocarpa       254 ISLCGWEPRALPYVVDCNDKPTQPVKDTGFTDSFHMVINGQHPSIRVRSVATE--QSVDT
G.max_1             258 ISLCGWKLRPLPYVVDWKDMSDQSLNNT--------------TNLVVHSAGTN--EHLKT
G.max_2             258 ISLCGWKLHPLPYVVDWKDMSDLSLNNS--------------TNLVVHSAGTN--ENSKT
S.lycopersicum      250 ISLCGWELRRLPYMVDPKDQLNQSSKDANLSEKSILSRKSEI--ITVYGSCTDKTSESKT
V.vinifera          256 ISLCGWKPRSLPYVVDSKDRQCKSAKDT--------------PPISVHSSATD--KSMQA

A.thaliana          322 NENPTLPDTLNSDPSSVVLDCKLCGACVGLWVFSTVPRPLELCRVTGDTEINIEKHPK--
P.patens            306 QGQIQSTGTDVSDPASAVLDCNLCGASVGLWNFATINRPAPLLNSGLEELFSS-KNRSSG
S.moellendorffi     286 -HELRVSLATGSDPCSAVLECRLCKASVGLWRFRTLSRSSLSITAILSTIEAS-AKKNVE
A.trichopoda        334 KESNTSFKEKEYDPASVVLDCKLCGASIALWTFSTVPQPLQSFNLIEQVEVHGIDSRCST
O.sativa            469 -IADARQAYQHYDPLSVVLDCQFCGACVALWPFSLVERPLQLFKLISDSSRQDEQTEGH-
Z.mays              334 -SVDVNQEDQHYDPLSVVLDCQFCGACVALWPFSLVQRPLQLFKLISDSNRQDDQDNGH-
P.trichocarpa       312 NEESGSCSGPHADSNAVVLDCRFCGASVGLWTFSMVPRPLELFKLVGYAEVNNNKNSGQ-
G.max_1             302 DESSKDSIGEQMDPNSAVLDCSLCGATIGLWAFCTVPRPVESIRLVGYAEVNGENAD---
G.max_2             302 DENSKDSIGEQMDPYSAVLDCTLCGATTGLWAFCTAPRPVESIRLVGYAEVNGGNAN---
S.lycopersicum      308 DDDNRASEEAIINPNSVVLDCKLCGACIGLWDFSMVSRPLEFLRVSGYTQVNNDHINHT-
V.vinifera          300 NEDPM-ASSELQSEHSVVLECSLCGATVGLWAFSTVQRPTEFFRLVGNSEVTAVMI----

A.thaliana          380 ---------------------------------------------GGTLQHQPSSLKF
P.patens            365 SNPVRDDA---AGAVGGPLLASPEAAEMVDAKPPEVVPV----NVLERAAPPKGVLDLKL
S.moellendorffi     344 VLPA--------GDVNAHV--DDNAAENIDTVNAEATISEDNDAAAVDDSKKNPGLDLTL
A.trichopoda        394 IACYISNASGVEGASNEHRIAN-------------CEIFHDDSAKEISTKQNDRNPSLNL
O.sativa            527 -----------A------------------------------GRVSGAGPSKTANIGFNF
Z.mays              392 -----------A------------------------------NIVSGVGHSKDANISFNF
P.trichocarpa       371 -----------DSANEN-HVD--------------NRGVIGNSASNGALSSMHRPSYLSF
G.max_1             359 -------------------LE--------------NRQGVNNTMPDVANSSKDTSSSLNM
G.max_2             359 -------------------LE--------------NRKGVNNTMPDVANSSKDTSSSLNM
S.lycopersicum      367 -----------HGDKNH-FSGNSGR---------DKSRECTGQVTTSANTMLDRRPPNFNL
V.vinifera          355 ------------------------------------------TDANGARSSNERLLNLNL
```

```
A.thaliana          393 TIAGGPPATKQNFKAT--ISLPIIGRNLRSRF----------------ASYSRDHDH----
P.patens            418 TIAGGPPPTRLIAPASVPPSFGIPGLPH---TVMQPRKIEAITYAAASYESRRPAHQGRG
S.moellendorffi     394 TIAGGPRPTRLSAPSP--ASIPIPGLNSQRHDDFQPRKSTLEQE---------PAE----
A.trichopoda        441 SIAGGPLPTKQKFHVT--VSLPIMTRHLLAGS----------------NSERRNWALL---
O.sativa            546 TIAGGPPPTRQNFRPR--VSLPVVSRHLKADL----------------SSHGHFISSG---
Z.mays              411 TIAGGPPPTRQSFRPK--VSFPVVSRHLKADL----------------NSRVNLLSSR---
P.trichocarpa       405 TIAGGPPPTKQNFKAT--ISLPVIGRNLRARF----------------SYDSDFRDRT---
G.max_1             386 TIAGGPPPTKQNFKAI--ISLPIIGQNLRARL----------------SYDSDFRDHV---
G.max_2             386 TIAGGPPPTRQNFKAI--ISLPIIGQNLRARL----------------SYDSDFRDHV---
S.lycopersicum      407 TIAGGPPPVTHDYRAK--ISLPIIGRNLRAWF----------------IAESELKDDL---
V.vinifera          373 TIAGGPPPTEQNFRAT--ISIPVIGQNLRARF----------------SSDHDFRDRS---

A.thaliana          432 ------------------------------------------------------------
P.patens            475 HNDTGTPTYHVEGEVIQNREMKDA---------EES-----------KDAEYFSKRKREK
S.moellendorffi     439 ------------KTTVQDRS--------------------------------SSKRKRDS
A.trichopoda        481 -----PLP-QEQQDYMQSTAHDINSAQCRNKHDQHYVHGQQGERVA---TGGMSKRRRSE
O.sativa            586 -----SDNHMVPVTLHASGLTKHKR---SMD-----------------------------
Z.mays              451 -----SDCYMVPVASHASGSMKRKR---STD-----------------------------
P.trichocarpa       445 -----CD----NQEFSQSGSENKNL---SSEERESAKH-NFGEQVSLLEAVGLLKSKTHD
G.max_1             426 -----FV----DRGGIQSDLQ-EK--------TDNIVNAFIGQLVPVSSEIR--------
G.max_2             426 -----FV----DRGGIQSDLQKEK--------TDNTVNASIGQLVPVSSEIR--------
S.lycopersicum      447 -----VT----KSSSGVSKNP-EFL---AGE--------------------------NTE
V.vinifera          413 -----CV----NQENSPSGANKKE--------------------------------SKRHG

A.thaliana          432 ------GDVSSIQDQ------QSR----------T-AEN---NG-D---VTQNSNQVMND
P.patens            515 GNDESEGVFSPNTKRKREVGSQWPGFTVKLPARDLPHASSVNAIDTCYPPKQ-ENSMESV
S.moellendorffi     455 -----ESA-----HR-----------KKLKAVDGLPGSSSVNAVETSYNHNRHENSAESV
A.trichopoda        532 -----EA--SLPTNH------LDP----------ISLDEKFSEVND---TP---------
O.sativa            609 ------ESHMLEGNN------TIS----------T-DAGTTTNGAD---HQRENSV-NGT
Z.mays              474 ------EPHLLEGDA------DDV----------D--TSTIGANHD---QPGENSE-KSI
P.trichocarpa       492 -----QGQCSYASGD------QSS----------C-LNSGSSEGDA---LRKENNSKMSL
G.max_1             460 ------EISNFETGS------QSS----------L-HDSDVLAGQS---SAL--KDKMPV
G.max_2             461 ------EISNFETGS------QAS----------L-HDSDVIAGQS---SGL--KDKMHV
S.lycopersicum      468 -----EG---------------SS----------L-STSEVSTE-----AQLENNQAA--
V.vinifera          433 -----DGQFNATSND------QSP----------C-LNNNISEEDD---AFRNSNNHMSL

A.thaliana          462 IGE---------------------------------------------------------
P.patens            574 DNLPLGSDGQGANTA------EYHVNGPESRVQAEHSMSR----QDGID-----------
S.moellendorffi     494 ECSPQGSDEEQDT-----------------------------------------------
A.trichopoda        557 ------MDKTFSS------------------EGESSKKQRQSSVNAIGACHTVIENST-
O.sativa            642 SN--------------------LVANPEHQQGGSHSDTSRVTSTGEVSN----------
Z.mays              506 SN--------------------LVVSTEQKQDGSHSNAAKDTELDEASN----------
P.trichocarpa       527 EGIDITREPAFPETAGYDSAVECLTQNPPNTVHGYDATDQLPENSNNIESHDSVVGVSGV
G.max_1             492 HT---DADKLNSSAA------------------GYPSS----------------------
G.max_2             493 HT---DADKLNSSTA------------------GYPST----------------------
S.lycopersicum      490 ------------------------------------------------------------
V.vinifera          468 ERPNVNQQ-------------------------GLDANM--TTRNAEIS-----------

A.thaliana          465 ------------------------------------------------------------
P.patens            613 ------------AGKSICGGRVEEGV-------QNNVVVICPGAGISGSHETEIQGVEAN
S.moellendorffi     507 ------------------------------------------------------------
A.trichopoda        591 ESVENRPQ-DSFESGVIFEGSRANDLGLEVGE--RNFANGLPTSSTKGAEIDN-------
O.sativa            671 ---EESE-TGHAAIKSHT---------------S------TDELGQHGSDPKS-------
Z.mays              535 ---ETQPETGSPLRKSIT--------------ST------YADADQHGLEPRF-------
P.trichocarpa       587 SQVSASSISGSAAISPIGSGKRSESNSLEMVTSSINHDEQIPGADISGSNVA--------
G.max_1             509 ------------------------------------------------------------
G.max_2             510 ------------------------------------------------------------
S.lycopersicum      490 ------------------------------------------AQVSGN-----------
V.vinifera          490 -----------------------EDDSLMVVG-----ADDCNLQQIHGTDSA--------
```

```
A.thaliana         465 ------------------------------------------------------------
P.patens           654 LERSESVAEFATDVIELMEEHVSGRGLMDEFIPEDTLKAIVTDDHEGSRQAMLGISHTFV
S.moellendorffi    507 ------------------------------------------------------------
A.trichopoda       641 ------------------------------------------------------------
O.sativa           699 ------------------------------------------------------------
Z.mays             565 ------------------------------------------------------------
P.trichocarpa      639 ------------------------------------------------------------
G.max_1            509 ------------------------------------------------------------
G.max_2            510 ------------------------------------------------------------
S.lycopersicum     496 ------------------------------------------------------------
V.vinifera         514 ------------------------------------------------------------

A.thaliana         465 -------------------K-----ADGGRNSTDVESDIALQNKDK--QMM---------
P.patens           714 KDTSSAGVSERYQRNVAQRD-ADEDANAGSTLE-------TT------NAIIQGVDMVAN
S.moellendorffi    507 ---------------VLSR----------------------E------KAAS--------
A.trichopoda       641 ---------------ELPKP-----SSN--GCHQSDEIIPANNEKQHKSSIEDGQPALSK
O.sativa           699 ----------------LPVEDSS----NAHDLA---KTCTNNSRPVQAATL---------
Z.mays             565 ----------------CSVQGTNEEPSGGVTLT---EMHANNSRSTEVSTI---------
P.trichocarpa      639 ----------------CQIG-----MRGGETCSDIENTLATQEKSQEG------------
G.max_1            509 ----------------SQKD-----STEGEAPSVSHKTLDGQVGSLENYGV---------
G.max_2            510 ----------------SQKD-----STEGEAPLVSHKTLDGEVGSLENYGV---------
S.lycopersicum     496 ------------------------------TTEMADNT---ESMNKVDPAV---------
V.vinifera         514 ----------------CNIH-----IRGGAKYSEVQNILSTQPNSQGDDWV---------

A.thaliana         490 ---------------VVRSNLPENNKPRD----------STAEKSATSNKQMEFDPIKQH
P.patens           760 IQVKEVTA------GRTVGESPSERNEEVQSHPSFPPGLLLDDVKKLEIQTGEFDPIRQH
S.moellendorffi    516 -------------------------------------QDVAKEAAIFSGSPSEFDPVHHH
A.trichopoda       679 IPTCLVTSTPLIAFNGVSCENKVT---------------SDSVAALGSGTNKEFDPIKHH
O.sativa           727 ---------------TK-----SSNDGEKG-------ASQPSGSQGLYDKLNEFDPMKQH
Z.mays             597 ---------------TE-----S------L-------VNREKEKQGLYDRMNEFDPIKQH
P.trichocarpa      666 ---------------TTGVQFPVNSEVVAN-------STGKDPKKLALDKALGFDPIRQH
G.max_1            539 ---------------KDREENPISRDNVLY-------SLGKLKNPPVSDKAMEFDPIRQH
G.max_2            540 ---------------KDREENPINRDNVHS-------SLGKLKNPTVSNKLMEFDPIRQH
S.lycopersicum     514 ---------------TDPCKDKVGNDFGSS-------SRGKELPILSLDKALEFDPFKLH
V.vinifera         544 ---------------KDRGQIPVNNEAVAC-------GIANDLKQQPIDKAMEFDPIWQH

A.thaliana         525 RHFCPWIWSTG---------RRGPGWRQTLSALQRHKGSCQTPP---------S--SSSL
P.patens           814 RHFCPWVNAHVAAATSGTGSSKFCGWQIVLDALQPQPPSPHQQHQHQQGSVESEFTGSKY
S.moellendorffi    539 RYFCPWISSNAA------DQSGKCGWQMTIDAIFSCAATNAKS-----G-VVSDRDKAAA
A.trichopoda       724 RYYCPWITSSGN------SDTGLAGWQQTLSALLKSEEAATHDS-------PMSPASTSL
O.sativa           760 RTFCPWICPDGG--------ETLPGWRLTLPALLSQDKRIDEDS-------QVEPQISLL
Z.mays             624 RTFCPWTSPDYG--------EPLPGWRLTLSALLAGDKRSDGDL-------QVDIQTSLL
P.trichocarpa      704 RHFCPWIISTS---------SGAPGWQQTLSALGRQKEFSLPST-------NSP--SSSL
G.max_1            577 RHFCPWIASIN---------DGEPGWKQTLSALYHQKNHLPHSP-------NRSPSSMPI
G.max_2            578 RHFCSWIASIH---------DGEPGWKQTLSALYHQKNHLPHSP-------NRFPSSMPI
S.lycopersicum     552 RYFCPWIASNG---------VSPSGWEQTLSALERHEESSSPLS-------NHA--PSSL
V.vinifera         582 RHFCPWIAAAG---------GAAPGWQQTLSALQQQKDFSHPSP-------------SNM

A.thaliana         565 FKVDDPLTSVRNLFKSPSPKKRKLNGGSSS-----
P.patens           874 KVQDDPILSVRKLLGSVSSNYLPSS----------
S.moellendorffi    587 VNKMDPLVSVRRMLGGKGKGVAVNGGAISMSRAGT
A.trichopoda       771 RYEVDPVASIRKLFQSPSSKRMKSA----------
O.sativa           805 SEEDDPVTSVRKLFMTPPSKKLRIHRAEKG-----
Z.mays             669 DEEDDPLTSVRKLFMTPPPKRRRIQQSEKS-----
P.trichocarpa      746 NKVDDPIALVRKLFTSP-VKRMKPTSGSS------
G.max_1            621 VKVDDPVGSIRKLFMSPPTKRLKSTHITGQNT---
G.max_2            622 VKVDDPVGSIRKLFMSPPSKRTESTHITGHNS---
S.lycopersicum     594 IKVDDPVASVQKLFTSPQAKRRKLVRPS-------
V.vinifera         620 IKVDDPIASVRKIFMSSSEKRMKRTHGST------
```

Figure S7: Amino acid sequence alignments

Amino acid sequence alignments of the indicated Arabidopsis proteins (identified in the *coi1* suppressor screen) with orthologs in other plant species and model species. (A) WRAP53; (B) SMU2; (C) ZCH1. Note that the sequence similarity of ZCH1 with the human protein is restricted to the zf-Z3HC domain (region extending from approximately amino acids 60-275 in the Arabidopsis protein). The zf-Z3HC domain is found in many functionally unrelated eukaryotic proteins. The plant orthologs of ZCH1 all share significant sequence similarity with each other throughout the entire protein. A second protein in Arabidopsis also contains a zf-Z3HC domain (At1g17210), but this protein is larger than ZCH1 (953 amino acids vs. 594 amino acids) and it does not share significant sequence similarity with ZCH1 beyond the zf-Z3HC domain. In contrast to ZCH1, At1g17210 does have putative homologs in other species, including humans (Gunkel and Cordes, 2022). We conclude that ZCH1 is a plant-specific protein encoded by a single copy gene in Arabidopsis.

Gunkel P, Cordes VC (2022) ZC3HC1 is a structural element of the nuclear basket effecting interlinkage of TPR polypeptides. Mol Biol Cell 33: ar2: doi: 10.1091/mbc.E22-02-0037.
