# Supplementary material for: A GFP splicing reporter in a coilin mutant background reveals links between alternative splicing, siRNAs, and coilin function in Arabidopsis thaliana
Source: G3 (Bethesda). 2023 Aug 4;13(10):jkad175. doi: 10.1093/g3journal/jkad175 (PMC10542627; doi:10.1093/g3journal/jkad175)
Supplement: jkad175_Supplementary_Data [file jkad175_supplementary_data.zip › Figure_S8_G3-2023-404387.pdf]

Figure S8: **a** *wrap53* mutation does not disperse CBs (Kanno et al)

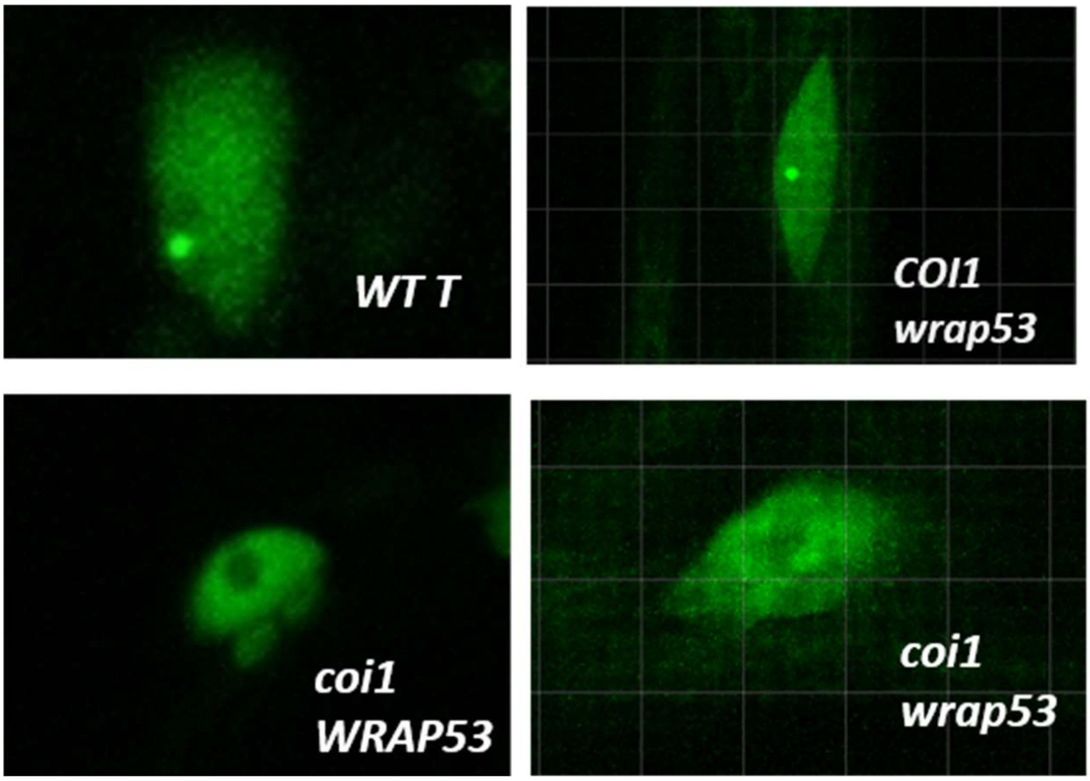

Figure S8: A *wrap53* mutation does not disrupt Cajal bodies in Arabidopsis

To examine whether *wrap53* mutations are able to disrupt CB structure, we used a fluorescence detection procedure described previously (Kanno et al., 2016). Briefly, a *coi1-8 wrap53-1* double mutant was crossed to a transgenic line expressing the CB-specific fluorescence marker U2B": GFP (Collier *et al*., 2008). The resulting F1 plants were allowed to self-fertilize to produce F2 seeds, which were germinated on solid MS medium containing phosphinothricin (PPT). F2 seedlings were selected for PPT resistance (contributed by the U2B": GFP line) and a GFP-negative phenotype, indicating absence of the *WT T* locus. Seedlings selected in this way were transferred to soil and later genotyped for the *wrap53* mutation with or without the *coi1* mutation. The presence of fluorescent CBs in trichome nuclei of *WT T* plants and the indicated combinations of *coi1* and *wrap53* mutations (shown in lower case letters; wild-type copies written in upper case letters) containing the U2B": GFP gene was assessed using a TCS LSI-III Confocal Microscope System. At least 20 leaf nuclei were examined for each genotype. **Top-left;** *WT T*, fluorescent CBs are visible. **Bottom left**: By contrast, CBs lose fluorescence in a *coi1* mutant that is wild-type for WRAP53. **Top right:** Notably, fluorescent CBs are also visible in the *wrap53* mutant that is wild-type for coilin (COI), indicating that the *wrap53* mutation alone does not disrupt CB integrity. **Bottom right**: CBs are not fluorescent in a *coi1 wrap53* double mutant, demonstrating that the *wrap53* mutation cannot rescue the loss of CB fluorescence caused by the *coi1* mutation.
