# Supplementary material for: A GFP splicing reporter in a coilin mutant background reveals links between alternative splicing, siRNAs, and coilin function in Arabidopsis thaliana
Source: G3 (Bethesda). 2023 Aug 4;13(10):jkad175. doi: 10.1093/g3journal/jkad175 (PMC10542627; doi:10.1093/g3journal/jkad175)
Supplement: jkad175_Supplementary_Data [file jkad175_supplementary_data.zip › Table_S1_G3-2023-404387.pdf]

Table S1: Mutants identified in the coilin suppressor screen and other mutants used in this study

Name	AGI number	ABRC Seed Stock Number	alleles/mutation	GFP phenotype	Predicted function	Reference	Accession numbers: RNA-seq, small RNAs, and DNA-seq data
**NEW MUTANTS CSS SCREEN** **(> 1 allele &/or complementation)**							
**WRAP53**	AT4G21520	CS72418	wrap53-1 (css5-3)<br>Gln304* (G to A);<br>chr 4: 11448305	GFP-weak in *coi*1 *wrap53*	WD40 repeat protein; Cajal body protein	This paper	***wrap53***:<br>small RNAs<br><br>SRR13267755<br>SAMN17103491<br><br>SRR13267754<br>SAMN17103492<br><br>RNAs<br><br>SRR13267733<br>SAMN17103504<br><br>SRR13267750<br>SAMN17103505<br><br>SRR13267749<br>SAMN17103506<br><br>DNAs<br><br>SRR13267740<br>SAMN17103515
		CS72419	wrap53-2 (css6-7)<br>Gln211* (G to A);<br>chr 4: 11449128			This paper	
**SMU2**	AT2G26460	CS72417<br><br><br><br><br>CS72658	smu2-1 (css3-2)<br>splice site (5''ss, 5th intron) C to T;<br>chr 2: 11258173<br><br>smu2-1_compl (smu2-1 (css3-2) complemented line)	GFP-weak in *coi1 smu2*	Predicted splicing factor	This paper	***smu2***:<br>small RNAs<br><br>SRR13267722<br>SAMN17103489<br><br>SRR13267756<br>SAMN17103490<br><br>RNAs<br><br>SRR13267729<br>SAMN17103501

							SRR13267730<br>SAMN17103502<br><br>SRR13267731<br>SAMN17103503<br><br><u>DNAs</u><br><br>SRR13267739<br>SAMN17103514<br><br>***smu2-1*** complemented:<br><br><u>RNAs</u><br><br>SRR14917397<br>SAMN19865507<br><br>SRR14917396<br>SAMN19865508<br><br>SRR14917395<br>SAMN19865509<br><br><u>small RNAs</u><br><br>SRR14917403<br>SAMN19865514<br><br>SRR14917402<br>SAMN19865515
		CS72630	smu2-2 (css42-2)<br>W508* (C to T);<br>chr 2: 11255284			This paper	
		CS72631	smu2-3 (css37-2)<br>R542H (CGT to CAT; G to A)<br>chr 2: 11255182			This paper	***smu2-3*:**<br><u>RNAs</u><br><br>SRR14917400<br>SAMN19865504<br><br>SRR14917399<br>SAMN19865505<br><br>SRR14917398<br>SAMN19865506<br><br><u>small RNAs</u><br><br>SRR14917405<br>SAMN19865512<br><br>SRR14917404<br>SAMN19865513
**ZCH1**	AT1G48950	CS72421	zch1-1 (css13-5)<br>3'ss, 2nd intron (AG to AA);<br>chr 1: 18107754	*GFP weak, enhanced' PTGS' in*	unknown C3HC zinc finger protein	This paper	***znf*:**<br><u>small RNAs</u><br><br>SRR13267753

				coi1 zch1-1			SAMN17103495<br><br>SRR13267752<br>SAMN17103496<br><br>RNAs<br><br>SRR13267746<br>SAMN17103510<br><br>SRR13267735<br>SAMN17103511<br><br>SRR13267736<br>SAMN17103512
		CS72422	zch1-2 (css21-1)<br>W352* (TGG to TGA);<br>chr 1: 18108886			This paper	
		CS72423	zch1--3 (css25-1)<br>W167* (TGG to TGA);<br>chr 1: 18108158			This paper	
		CS72424	zch1-4 (css23-2)<br>W541* (TGG to TAG);<br>chr 1: 18109545			This paper	
		CS72425	zch1-5 (css18-2)<br>Gln386*(CAA to TAA);<br>chr 1: 18108986			This paper	
**MAD1**	AT5G49880	CS72416	mad1-3 (css2-4)<br>Arg162* (CGA to TGA);<br>chr 5: 20283048	GFP-weak in *coi1 mad1*	mitotic spindle assembly checkpoint protein	This paper	
		CS72632	mad1-4 (css15-6)<br>Glu315Lys (GAA to AAA);<br>chr 5: 202840291			This paper	
**SDE2-like**	At4g01000	CS72633	sde2-1 (css26-4)<br>Trp122" or Trp127*? (C to T)<br>chr 4: 433348	GFP-weak in *coi1 sde2-like*	Ubiquitin family protein, Splicing, C complex	Koncz et al., ;<br>This paper	
		CS72634	sde2-2 (css21-5)<br>Gly86Glu (C to T)<br>chr 4: 433471			This paper	
**NEW GFP ALLELES**							
**GFP**	GFP	CS72635	gfp-1 (css34-1)<br>L60P (CTC to CCC);<br>179	GFP negative		This paper	

	GFP	CS72636	gfp-2 (css29-1) (first one found) 35S promoter splice site (AG to AA)			This paper	
	GFP	CS72637	gfp-3 (css19-8) (first one found) A179T (GCC to ACC); 535			This paper	
	GFP	CS72638	gfp-4 (css32-1) C48Y (TGC to TAC); 143			This paper	
	GFP	CS72639	gfp-5 (css6-6) (first one found) P58L (CCC to CTC;) 173			This paper	
	GFP	CS72640	gfp-6 (css13-1) (first one found) G104D (GGC to GAC); 311			This paper	
**NEW ALLELES** **Of factors identified in previous splicing screen**							
**PRP4Ka**	At3g25840	CS72641	prp4ka-5 (css7-8) Trp360* (C to T) chr 3: 9456029	GFP weak		This paper	
	At3g25840	CS72642	prp4ka-6 (css11-4) Arg254His (C to T) chr 3:9670352			This paper	
		n.a.	prp4ka-7 (css17-4) G809D (C to T) chr 3: 9453637			This paper	
		CS72643	prp4ka-8 (css19-3) Arg47* (G to A) chr 3: 9457308			This paper	
		CS72644	prp4ka-9 (css21-3) 5' ss, first intron (C to T) chr 3: 9457089			This paper	
		CS72645	prp4ka-10 (css25-4) Gly624Glu (C to T) chr 3:9454450			This paper	
		n.a.	prp4ka-11 (css22-1)			This paper	

			Gly719Asp (C to T)<br>chr 3: 9454065				
**SAC3a**	AT2G39340	CS72646	sac3a-8 (css7-8)<br>Trp306* (TGG to TGA);<br>chr 2:16425626	GFP weak		This paper	
		CS72647	sac3a-9 (css32-2)<br>Gln192* (C to T)<br>chr 2: 16425282			This paper	
**CBP80**	At2g13540	CS72648	cbp80-2 (css1-1)<br>W430*(G to A）<br>chr 2: 5641098	GFP weak		This paper	
		CS72649	cbp80-3 (css2-5)<br>W520* (TGG to TGA) chr 2: 5641569			This paper	
		CS72650	cbp80-4 (css6-13)<br>5' ss_8th intron (GT to AT)<br>chr 2: 5639118			This paper	
		n.a.	(css7-1)<br>R669* (CGA to TGA);<br>chr 2: 5642204			This paper	
		CS72651	cbp80-5 (css17-2) D57N (D597N?) (GAT to AAT);<br>chr 2: 5641798			This paper	
**SUS2/PRP8a**	AT1G80070	CS72652	prp8-12 (css16-2)<br>Gly1820Arg (G1820R) (GGA to AGA);<br>chr 1:30125294	GFP weak		This paper	
**PRP18**	At1g03140	n.a.	css40-1<br>G63R (GGA to AGA)<br>chr 1: 756037	GFP weak		This paper	
**T line (WT)**	n.a.	CS69640		Wild-type GFP (intermediate)	Wild-type line expressing *GFP* reporter gene; used for EMS mutagenesis	Kanno et al., 2008	**T line:**<br>small RNAs<br><br>SRR13267760<br>SAMN17103474<br><br>SRR13267719<br>SAMN17103475<br><br>SRR13267724<br>SAMN17103476<br><br>control for all RNA-seq and

							DNA-seq experiments
**coilin**	At1g13030	CS69632<br><br>CS69639	hgf1-1/R40* (C118T)<br><br>hgf1-8/P439L (C2521T)	Hyper-GFP		Kanno et al., 2016<br><br>Kanno et al., 2016	***hgf1-1***:<br>small RNAs<br><br>SRR13267745<br>SAMN17103483<br><br>***hgf1-8***:<br>small RNAs<br><br>SRR13267720<br>SAMN17103484<br><br>SRR14917394<br>SAMN19865510<br><br>SRR14917406<br>SAMN19865511<br><br>RNAs<br><br>SRR14917408<br>SAMN19865501<br><br>SRR14917407<br>SAMN19865502<br><br>SRR14917401<br>SAMN19865503
**CWC16a**	At1g25682	CS69846, CS72366, CS72367	cwc16a-1: (C to T, Chr 1:900354)<br><br>cwc16a-2: (C to T, Chr 1:9004043, W18*)<br><br>cwc16a-3: (C to T, Chr 1:9002886, splice site mutation)	Hyper-GFP		Kanno et al., 2017<br><br>This paper<br><br>This paper	***cwc16a***:<br>small RNAs<br><br>SRR13267751<br>SAMN17103497<br><br>SRR13267726<br>SAMN17103498<br><br>***cwc16a-1/dcl4***<br>small RNAs<br><br>SRR13267727<br>SAMN17103499<br><br>SRR13267728<br>SAMN17103500
**OTHER mutants used in this study**							
**DCL2**	AT3G03300	provided by Hervé Vaucheret	dcl2 -Kas			Parent et al. 2014	***dcl2/dcl4***:<br>small RNAs<br><br>SRR15185391<br>SAMN20298928<br><br>SRR15185390<br>SAMN20298929

**DCL3**	AT3G43920	CS69179	dcl3-5			Daxinger et al. 2009	***dcl3/dcl4***:<br>small RNAs<br><br>SRR15185389<br>SAMN20298930<br><br>SRR15185388<br>SAMN20298931
**DCL4**	AT5G20320	provided by Scott Poethig	dcl4-12: Ch5 C6866157T 8th intron acceptor site			provided by Scott Poething	***T/dcl4:***<br>small RNAs<br><br>SRR13267732<br>SAMN17103477<br><br>SRR13267738<br>SAMN17103478<br><br>SRR13267741<br>SAMN17103479<br><br>***hgf1-1/dcl4***:<br>small RNAs<br><br>SRR13267759<br>SAMN17103485<br><br>SRR13267758<br>SAMN17103486<br><br>SRR15185396<br>SAMN20298924<br><br>SRR15185395<br>SAMN20298925<br><br>***dcl4***:<br>small RNAs<br><br>SRR15185387<br>SAMN20298932<br><br>SRR15185386<br>SAMN20298933<br><br>SRR15185394<br>SAMN20298934
**RDR6**			rdr6-14: Ch3 C18349667T W1039*			Peragine et al., 2004	**T/*rdr6*:**<br>small RNAs<br><br>SRR13267742<br>SAMN17103480<br><br>SRR13267743<br>SAMN17103481<br><br>SRR13267744<br>SAMN17103482

							hgf1-1/rdr6:<br>small RNAs<br><br>SRR13267757<br>SAMN17103487<br><br>SRR13267721<br>SAMN17103488<br><br>SRR15185393<br>SAMN20298926<br><br>SRR15185392<br>SAMN20298927

Study: SRP298335

Bioproject accession: PRJNA686012

References

Daxinger, L., Kanno, T., Bucher, E., van der Winden, J., Naumann, U., Matzke, A. J., & Matzke, M. (2009). A stepwise pathway for biogenesis of 24-nt secondary siRNAs and spreading of DNA methylation. The EMBO journal, 28(1), 48–57. https://doi.org/10.1038/emboj.2008.260

Kanno, T., Lin, W. D., Fu, J. L., Wu, M. T., Yang, H. W., Lin, S. S., Matzke, A. J., & Matzke, M. (2016). Identification of Coilin Mutants in a Screen for Enhanced Expression of an Alternatively Spliced GFP Reporter Gene in Arabidopsis thaliana. Genetics, 203(4), 1709–1720. https://doi.org/10.1534/genetics.116.190751

Kanno, T., Lin, W. D., Fu, J. L., Matzke, A., & Matzke, M. (2017). A genetic screen implicates a CWC16/Yju2/CCDC130 protein and SMU1 in alternative splicing in Arabidopsis thaliana. RNA (New York, N.Y.), 23(7), 1068–1079. https://doi.org/10.1261/rna.060517.116

Kanno, T., Bucher, E., Daxinger, L., Huettel, B., Böhmdorfer, G., Gregor, W., Kreil, D. P., Matzke, M., & Matzke, A. J. (2008). A structural-maintenance-of-chromosomes hinge domain-containing protein is required for RNA-directed DNA methylation. Nature genetics, 40(5), 670–675. https://doi.org/10.1038/ng.119

Parent, J.-S., Bouteiller, N., Elmayan, T. and Vaucheret, H. (2015), Respective contributions of Arabidopsis DCL2 and DCL4 to RNA silencing. Plant J, 81: 223-232. https://doi.org/10.1111/tpj.12720

Peragine A, Yoshikawa M, Wu G, Albrecht HL, Poethig RS. SGS3 and SGS2/SDE1/RDR6 are required for juvenile development and the production of trans-acting siRNAs in Arabidopsis. Genes Dev. 2004;18(19):2368-2379. doi:10.1101/gad.1231804
