# Supplementary material for: A GFP splicing reporter in a coilin mutant background reveals links between alternative splicing, siRNAs, and coilin function in Arabidopsis thaliana
Source: G3 (Bethesda). 2023 Aug 4;13(10):jkad175. doi: 10.1093/g3journal/jkad175 (PMC10542627; doi:10.1093/g3journal/jkad175)
Supplement: jkad175_Supplementary_Data [file jkad175_supplementary_data.zip › Table_S2_G3-2023-404387.pdf]

Table S2: Summary of Coilin IP-MS

splicing factors							
-Sm proteins-							
**AGI number**	**Protein name** B/B′, D1, D2, D3, E, F, and G	**Freq (mRED x2)**	**Freq (N-FLAG x2)**	**Freq (FLAG-C x2)**	**Total count**	**Freq (BGx2)**	**Reference (Koncz et al., 2012)**
**AT2G47640**	Small nuclear ribonucleoprotein family protein (SMD2 – NCBI)	2	2	2	6	1	Sm core proteins
**AT3G11500**	Small nuclear ribonucleoprotein family protein (SMG - NCBI)	2	1	1	4	1	Sm core proteins
**AT2G18740**	Small nuclear ribonucleoprotein family protein (SME1 – temperature-specific splice regulator porcupine – TAIR)	0	2	1	3	1	Sm core proteins
**AT2G23930**	Putative small nuclear ribonucleoprotein G	2	0	1	3	0	Sm core proteins
**AT3G07590**	Small nuclear ribonucleoprotein family protein (SMD1)	1	2	0	3	1	Sm core proteins Elvira-Matelot et al (2016)
**AT4G02840**	Small nuclear ribonucleoprotein family protein (SMD1B)	1	2	0	3	1	Sm core proteins Elvira-Matelot et al (2016)
**AT1G20580**	Small nuclear ribonucleoprotein family protein (SMD3- TAIR))	0	1	1	2	0	Sm core proteins
**AT4G30220**	small nuclear ribonucleoprotein (SMF-F)	0	2	0	2	1	Sm core proteins Kanno et al (2017)
-Other splicing factors-							
**AGI number**	**Protein name**	**Freq (mRED x2)**	**Freq (N-FLAG x2)**	**Freq (FLAG-C x2)**	**Total count**	**Freq (BGx2)**	**Reference (Koncz et al., 2012; TAIR)**

**AT1G56070**	Ribosomal protein S5/Elongation factor G/III/V family protein (cold-induced translation- TAIR)	2	2	2	6	1	U5 snRNP
**AT5G02500**	heat shock cognate protein 70-1	2	2	2	6	1	NTC-associated
**AT3G09440**	Heat shock protein 70 (Hsp 70) family protein (chaperone)	1	2	2	5	1	Abundant first in C complex
**AT4G38740**	rotamase CYP 1	1	2	2	5	1	NTC-associated
**AT5G20160**	Ribosomal protein L7Ae/L30e/S12e/Gadd45 family protein	1	2	2	5	1	U4/U6 snRNP
**AT1G18080**	Transducin/WD40 repeat-like superfamily protein (RACK1 – hormone, stress signaling – TAIR)	0	2	2	4	1	Abundant first in C complex
**AT2G16600**	rotamase CYP 3	0	2	2	4	1	NTC-associated
**AT2G21660**	cold, circadian rhythm, and rna binding 2(AtGRP7 – TAIR)	0	2	2	4	1	Glycine-rich RNA binding proteins
**AT4G39260**	cold, circadian rhythm, and RNA binding 1 (e)	0	2	2	4	1	Glycine-rich RNA binding proteins
**AT5G04280**	RNA-binding (RRM/RBD/RNP motifs) family protein with retrovirus zinc finger-like domain-containing protein (RZ-1C; cold tolerance. TAIR)	0	2	2	4	1	Glycine-rich, Zn finger; hnRNP family
**AT5G52040**	RNA-binding (RRM/RBD/RNP motifs) family protein (RS41 – TAIR)	0	2	2	4	1	SR proteins

**AT4G12600**	Ribosomal protein L7Ae/L30e/S12e/Gadd45 family protein	1	0	2	3	0	U4/U6 snRNP
**AT5G47210**	Hyaluronan / mRNA binding family (mRNA binding protein, stress? – TAIR)	0	2	1	3	1	hnRNP family
**AT1G06220**	Ribosomal protein S5/Elongation factor G/III/V family protein (similarity to splicing factor Snu114 – TAIR)	0	2	0	2	1	U5 snRNP
**AT1G23860**	RS-containing zinc finger protein 21 (RSZ21 – TAIR)	0	2	0	2	1	SR proteins
**AT1G77180**	chromatin protein family (SKIP – spliceosome_environmental stress – TAIR)	0	1	1	2	0	Core NTC
**AT3G12580**	heat shock protein 70	1	1	0	2	0	NTC-associated
**AT3G56070**	rotamase cyclophilin 2 (signal transduction – TAIR)	0	0	2	2	0	U4/U6 snRNP
**AT4G34870**	rotamase cyclophilin 5 (ROC5)	0	1	1	2	0	NTC-associated
**AT5G25230**	Ribosomal protein S5/Elongation factor G/III/V family protein (splicing, catalytic step 2 spliceosome - TAIR)	0	2	0	2	1	U5 snRNP
**AT5G58470**	TBP-associated factor 15B (TAF15B –autonomous flowering pathway, represses FLC – TAIR – RdDM Slotkin)	0	2	0	2	1	hnRNP family

nucleolar proteins (fibrillarin 1 and 2, NOP56-like (2X), NOP10-like and nucleolin-like							
**AGI number**	**Protein name**	**Freq (mRED x2)**	**Freq (N-FLAG x2)**	**Freq (FLAG-C x2)**	**Total count**	**Freq (BGx2)**	**Reference TAIR**
**AT1G56110**	NOP56-like pre rRNA processing ribonucleoprotein	2	1	1	4	0	
**AT3G05060**	NOP56-like pre rRNA processing ribonucleoprotein	2	1	1	4	1	
**AT5G52470**	fibrillarin 1 binds C/D snoRNAs	2	1	1	4	1	
**AT1G48920**	nucleolin like 1	0	2	1	3	1	
**AT4G25630**	fibrillarin 2	0	1	1	2	1	
**AT2G20490**	nucleolar RNA-binding Nop10p family protein	0	0	1	1	0	
**AT3G12860**	NOP56-like pre rRNA processing ribonucleoprotein	0	0	1	1	0	

nuclear transport factors (alpha-importin isoforms 1 and 2, NTF2, ALY/TREX							
**AGI number**	**Protein name**	**Freq (mRED x2)**	**Freq (N-FLAG x2)**	**Freq (FLAG-C x2)**	**Total count**	**Freq (BGx2)**	**Reference TAIR**
**AT4G16143**	importin alpha isoform 2	1	2	2	5	1	
**AT5G43960**	Nuclear transport factor 2 (NTF2) family protein with RNA binding (RRM-RBD-RNP motifs) domain-containing protein	0	1	1	2	1	
**AT5G59950**	RNA-binding (RRM/RBD/RNP motifs) family protein (ALY1, TREX complex; mRNA transport – TAIR)	0	2	0	2	1	

histones, HAs and HDACs

AGI number	Protein name	Freq (mRED x2)	Freq (N-FLAG x2)	Freq (FLAG-C x2)	Total count	Freq (BGx2)	Reference TAIR
**AT5G22650**	histone deacetylase 2B (member of plant-specific HDAC – TAIR)	2	2	2	6	1	nucleolar
**AT1G52740**	histone H2A protein 9	1	2	2	5	1	
**AT3G27360**	Histone superfamily protein (histone H3.1 – TAIR)	1	2	2	5	1	
**AT3G45980**	Histone superfamily protein (histone H2B – TAIR)	2	2	1	5	1	
**AT3G46030**	Histone superfamily protein (HTB11 – TAIR)	2	2	1	5	1	
**AT4G40030**	Histone superfamily protein (histone H3.3, HTR4 – TAIR)	1	2	2	5	1	
**AT5G03740**	histone deacetylase 2C (ABA and stress responses – TAIR)	1	2	2	5	1	nucleolar
**AT5G10980**	Histone superfamily protein (histone H3.3, HTR8 – TAIR)	1	2	2	5	1	
**AT5G59970**	Histone superfamily protein (no more info from TAIR)	1	2	2	5	1	
**AT5G65350**	histone 3 11 (HTR11 – TAIR)	1	2	2	5	1	
**AT3G44750**	histone deacetylase 3 (leaf polarity – TAIR)	1	2	1	4	1	nucleolar
**AT1G07660**	Histone superfamily protein (no other info TAIR)	0	1	2	3	1	
**AT1G07790**	Histone superfamily protein (histone H2B, HTB1 – TAIR)	2	1	0	3	0	

**AT2G28720**	Histone superfamily protein (no other info from TAIR)	2	1	0	3	0	
**AT2G37470**	Histone superfamily protein (no other info from TAIR)	2	1	0	3	0	
**AT5G02570**	Histone superfamily protein (no other info from TAIR)	2	1	0	3	0	
**AT5G22880**	histone B2 (histone H2B – TAIR)	2	1	0	3	0	
**AT5G56740**	histone acetyltransferase of the GNAT family 2 (histone H4 acetylation, chromatin silencing at Telomere - TAIR)	0	1	2	3	1	nucleolar
**AT5G59910**	Histone superfamily protein (HTB4 – TAIR)	2	1	0	3	0	
**AT1G13370**	Histone superfamily protein (no other info on TAIR)	1	0	1	2	0	
**AT5G27670**	histone H2A 7 (H2A7 – TAIR)	1	1	0	2	1	

Other proteins

AGI number	Protein name	Freq (mRED x2)	Freq (N-FLAG x2)	Freq (FLAG-C x2)	Total count	Freq (BGx2)	Reference TAIR
**AT3G18780**	actin 2	2	1	2	6	1	
**AT3G01500**	carbonic anhydrase 1	2	2	2	6	1	
**AT3G13920**	eukaryotic translation initiation factor 4A1	2	2	2	6	1	
**AT3G49010**	breast basic conserved 1	2	1	2	5	1	
**AT1G20620**	catalase 3	2	2	1	5	1	
**AT1G54270**	eif4a-2	1	2	2	5	1	
**AT2G36530**	Enolase	1	2	2	5	1	
**AT3G14240**	Subtilase family protein	2	2	1	5	0	

**AT5G26000**	thioglucoside glucohydrolase 1	1	2	2	5	1
**AT4G14960**	Tubulin/FtsZ family protein	2	1	2	5	1
**AT5G03690**	Aldolase superfamily protein	1	2	1	4	1
**AT3G14415**	Aldolase-type TIM barrel family protein	2	1	1	4	0
**AT3G15950**	DNA topoisomerase-like protein	0	2	2	4	1
**AT2G33040**	gamma subunit of Mt ATP synthase	1	1	2	4	0
**AT5G40370**	Glutaredoxin family protein	0	2	2	4	1
**AT3G25530**	glyoxylate reductase 1	0	2	2	4	1
**AT4G02930**	GTP binding Elongation factor Tu family protein	1	2	1	4	1
**AT4G14880**	O-acetylserine (thiol) lyase (OAS-TL) isoform A1	0	2	2	4	1
**AT4G13940**	S-adenosyl-L-homocysteine hydrolase	1	1	2	4	1
**AT1G07080**	Thioredoxin superfamily protein	0	2	2	4	1
**AT5G11420**	transmembrane protein, putative (Protein of unknown function, DUF642)	1	1	2	4	0
**AT3G46000**	actin depolymerizing factor 2	0	1	2	3	1
**AT2G23390**	acyl-CoA	0	1	2	3	0
**AT3G08580**	ADP/ATP carrier 1	0	2	1	3	1
**AT3G09980**	ankyrin repeat 30A-like protein (DUF662)	0	1	2	3	0
**AT3G08030**	DNA-directed RNA polymerase subunit beta (Protein of unknown function, DUF642)	0	1	2	3	0

**AT2G30950**	FtsH extracellular protease family	0	2	1	3	1
**AT5G26710**	Glutamyl/glutaminyl-tRNA synthetase, class Ic	0	1	2	3	0
**AT1G11860**	Glycine cleavage T-protein family	1	0	2	3	0
**AT3G07050**	GTP-binding family protein	2	0	1	3	0
**AT5G42020**	Heat shock protein 70 (Hsp 70) family protein	1	1	1	3	1
**AT5G56030**	heat shock protein 81-2	1	1	1	3	0
**AT1G66240**	homolog of anti-oxidant 1	0	1	2	3	0
**AT3G02090**	Insulinase (Peptidase family M16) protein	1	0	2	3	0
**AT4G31300**	N-terminal nucleophile aminohydrolases (Ntn hydrolases) superfamily protein	0	1	2	3	0
**AT3G62030**	rotamase CYP 4	0	1	2	3	1
**AT1G66580**	senescence associated gene 24	0	1	2	3	1
**AT1G24510**	TCP-1/cpn60 chaperonin family protein	0	2	1	3	1
**AT3G11830**	TCP-1/cpn60 chaperonin family protein	0	2	1	3	1
**AT3G18190**	TCP-1/cpn60 chaperonin family protein	0	1	2	3	0
**AT2G36410**	transcriptional activator (DUF662)	0	1	2	3	0
**AT3G52920**	transcriptional activator (DUF662)	0	1	2	3	0

**AT3G53990**	Adenine nucleotide alpha hydrolases-like superfamily protein	0	0	2	2	0
**AT5G03300**	adenosine kinase 2	0	1	1	2	0
**AT1G28290**	arabinogalactan protein 31	0	2	0	2	1
**AT5G65010**	asparagine synthetase 2	0	1	1	2	0
**AT5G14060**	Aspartate kinase family protein	1	1	0	2	0
**AT1G66410**	calmodulin 4	0	1	1	2	0
**AT2G27030**	calmodulin 5	0	1	1	2	0
**AT3G43810**	calmodulin 7	0	1	1	2	0
**AT1G08830**	copper/zinc superoxide dismutase 1	0	0	2	2	0
**AT2G29560**	cytosolic enolase	0	1	1	2	0
**AT2G33830**	Dormancy/auxin associated family protein	0	1	1	2	1
**AT2G41475**	Embryo-specific protein 3, (ATS3)	0	2	0	2	1
**AT5G15230**	GAST1 protein homolog 4	1	1	0	2	0
**AT4G09000**	general regulatory factor 1	1	1	0	2	0
**AT2G02930**	glutathione S-transferase F3	0	0	2	2	0
**AT5G56000**	HEAT SHOCK PROTEIN 81.4	1	1	0	2	0
**AT5G52640**	heat shock-like protein	0	1	1	2	0
**AT4G22670**	HSP70-interacting protein 1	0	0	2	2	0
**AT1G17860**	Kunitz family trypsin and protease inhibitor protein	0	0	2	2	0
**AT5G56290**	peroxin 5	0	1	1	2	0
**AT2G29650**	phosphate transporter 4;1	2	0	0	2	0
**AT1G79550**	phosphoglycerate kinase	0	1	1	2	0

**AT3G16420**	PYK10-binding protein 1	0	1	1	2	0
**AT1G58270**	TRAF-like family protein	1	1	0	2	0
**AT1G75780**	tubulin beta-1 chain	1	1	0	2	1
**AT3G29360**	UDP-glucose 6-dehydrogenase family protein	1	0	1	2	0
**AT5G15490**	UDP-glucose 6-dehydrogenase family protein	1	0	1	2	0
**AT5G39320**	UDP-glucose 6-dehydrogenase family protein	1	0	1	2	0

Table S2. Summary of Coilin IP-MS

Two independent experiments were performed for each of the three epitope-tagged coilin proteins (six experiments total). Frequency columns indicate the number of times a coilin interaction was detected for the indicated protein in the six experiments. The Freq BG indicates the number of times a protein was detected in two independent experiments (one for mRED, one for FLAG) without IP, i.e., background noise.
These results are in accord with earlier findings showing that metazoan coilin can interact with Sm proteins and several nucleolar proteins (Machnya et al., 2015; Xu et al., 2005). Abbreviation: TAIR – The Arabidopsis Information Resource (https://www.arabidopsis.org/), NTC – Nine Teen complex

References

Elvira-Matelot E, Bardou F, Ariel F, Jauvion V, Bouteiller N, Le Masson I, Cao J, Crespi MD, Vaucheret H (2016) The nuclear ribonucleoprotein SmD1 interplays with splicing, RNA quality control, and posttranscriptional gene silencing in Arabidopsis. Plant Cell 28:426-38. doi: 10.1105/tpc.15.01045.

Kanno T, Lin WD, Fu JL, Matzke AJM, Matzke M (2017) A genetic screen implicates a CWC16/Yju2/CCDC130 protein and SMU1 in alternative splicing in *Arabidopsis thaliana*. RNA 23:1068-1079.

Koncz, C., F. Dejong, N. Villacorta, D. Szakonyi, and Z. Koncz, 2012, The spliceosome-activating complex: molecular mechanisms underlying the function of a pleiotropic regulator. Front. Plant Sci. 3: 9. https://doi.org/10.3389/fpls.2012.00009

Machnya M, Neugebauer KM, Staněk D (2015) Coilin: the first 25 years. RNA Biology: 12: 590-596

Xu H, Pillai RS, Azzouz TN, Shpargel KB, Kambach C, Hebert MD, Schümperli D, Matera AG (2005) The C-terminal domain of coilin interacts with Sm proteins and U snRNPs. Chromosoma 114:155-166
