# Supplementary material for: A GFP splicing reporter in a coilin mutant background reveals links between alternative splicing, siRNAs, and coilin function in Arabidopsis thaliana
Source: G3 (Bethesda). 2023 Aug 4;13(10):jkad175. doi: 10.1093/g3journal/jkad175 (PMC10542627; doi:10.1093/g3journal/jkad175)
Supplement: jkad175_Supplementary_Data [file jkad175_supplementary_data.zip › Table_S3_G3-2023-404387.pdf]

*Table S3: Summary of bisufite sequencing experiments to detect DNA methylation in the *GFP* upstream region

(A) GFP

Genotype	methylated CG	unmethylated CG	methylated CHG	unmethylated CHG	methylated CHH	unmethylated CHH	# of clones
WT T	11/110 (**10.00**)	99/110 (**90.00**)	0/120 (**0.00**)	120/120 (100.00)	29/461 (6.29)	432/461 (**93.71**)	10
coi1-1	11/110 (**10.00**)	99/110 (**90.00**)	0/120 (**0.00**)	120/120 (100.00)	29/461 (6.29)	432/461 (**93.71**)	10
coi1-1 zch1-4	11/110 (**10.00**)	99/110 (**90.00**)	0/120 (**0.00**)	120/120 (100.00)	25/461 (5.42)	436/461 (**94.58**)	10
coi1-1 zch1-4	11/110 (**10.00**)	99/110 (**90.00**)	3/120 (**2.5**)	117/120 (97.5)	28/467 (6.00)	439/467 (**94.00**)	10

(B) PHAVOLUTA

Genotype	methylated CG	unmethylated CG	methylated CHG	unmethylated CHG	methylated CHH	unmethylated CHH	# of clones
WT T	4/117 (3.42)	113/117 (96.58)	1/162 (0.62)	161/162 (99.38)	8/456 (1.75)	448/456 (98.25)	9
coi1-1	0/130 (0.00)	130/130 (100.00)	0/180 (0.00)	180/180 (100.00)	12/508 (2.36)	496/508 (97.64)	10
coi1-1 zch1-1	0/103 (0.00)	103/103 (100.00)	0/144 (0.00)	144/144 (100.00)	14/405 (3.46)	391/405 (96.54)	8
coi1-1 zch1-4	0/117 (0.00)	117/117 (100.00)	0/162 (0.00)	162/162 (100.00)	13/458 (2.84)	445/458 (97.16)	9

Legend: The table shows the numbers of methylated and unmethylated cytosines and percentage of total in each of the indicated sequence contexts (CG. CHG, CHH) in *coi1* and *zchl* mutants. The region tested for methylation in the *GFP* upstream region is indicated by the opposing blue arrows in Fig. 1, top. Previous work has shown that siRNAs direct to this region, which contains a short tandem repeat, can trigger TGS of the *GFP* reporter gene (Kanno et al., 2008). The *WT T* line is wild-type for *ZCH* and *COI.* PHAVOLUTA is used as an unmethylated control (Bao et al, 2004; Reinders et al. (2008). Bisulfite sequencing as carried out as described in Daxinger et al (2009) using the primers listed below.

For GFP (top strand):

Top2F GCG GTG TYA TYT ATG TTA YTA GAT
Top2R CTT CTT RAT RTT CCA TAR CTT TCC

For PHV (this was performed by nested PCR)
Primary PCR
PHAV_P-F GTG YAG ATY TGT TTG GAG YTG ATT Y
PHAV_P-R TTT AAT ATC TAA CAT AAC CAA CCT TT

Secondary PCR
PHAV_S-F2 GGA YYA TAG TGA TGY YAT ATT GTG
PHAV_S-R TAT CAT CAA CAA CTT TCC ACA CC

REFERENCES

Bao N, Lye KW, Barton MK (2004) MicroRNA binding sites in Arabidopsis class III HD-ZIP mRNAs are required for methylation of the template chromosome. Dev Cell 7: 653–662

Daxinger L, Kanno T, Bucher E, van der Winden J, Naumann U, Matzke AJ, Matzke M (2009) A stepwise pathway for biogenesis of 24-nt secondary siRNAs and spreading of DNA methylation. EMBO J 28:48-57.

Kanno T, Bucher E, Daxinger L, Huettel B, Böhmdorfer G, Gregor W, Kreil DP, Matzke M, Matzke AJ (2008) A structural-maintenance-of-chromosomes hinge domain-containing protein is required for RNA-directed DNA methylation. Nat Genet 40:670-675.

Reinders J, Delucinge Vivier C, Theiler G, Chollet D, Descombes P, Paszkowski J (2008) Genome-wide, high-resolution DNA methylation profiling using bisulfite-mediated cytosine conversion. Genome Res 18: 469–476
