# Supplementary material for: A GFP splicing reporter in a coilin mutant background reveals links between alternative splicing, siRNAs, and coilin function in Arabidopsis thaliana
Source: G3 (Bethesda). 2023 Aug 4;13(10):jkad175. doi: 10.1093/g3journal/jkad175 (PMC10542627; doi:10.1093/g3journal/jkad175)
Supplement: jkad175_Supplementary_Data [file jkad175_supplementary_data.zip › Table_S4_G3-2023-404387.pdf]

Table S4: Summary of differential alternative-splicing (DAS) events in *smu2* mutants. For comparison to *smu2*, the DAS results for two other mutants identified in the *coi1-8* suppressor screen (*wrap53* and *zc3hc1*) are shown.

The most striking changes in *smu2* mutants concern the number of IRs (roughly 10X more), ES (roughly 6X more) and 5’ss (roughly 3-4X more) compared to the other two mutants shown (*wrap53* and *zc3hc1*). The high number of IR events was observed in two independent experiments (reps) of *smu2-1* as well in a second allele, *smu2-3*. The high numbers of IRs return to a more ‘normal’ value when compared to *coi1-8* that is heterozygous for the *smu2-1* allele (last column). **Abbreviations:** IR, intron retention; ES, exon skipping; 5’_ss, alternative 5’ splice site; 3’_ss, alternative 3’ splice site; 5’/3’_ss, alternative 5’ and 3’ splice sites

**Event**		**Number of DAS events**					
		coi1-8 wrap53	***coi1-8 zc3hc1***	***coi1-8 smu2-1* (rep. 1)**	***coi1-8 smu2-1* (rep. 2)**	***coi1-8 smu2-3***	***SMU2/smu2-1* vs. *coi1-8***
**Ref**	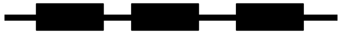	**n.a.**	**n.a.**	**n.a.**	**n.a.**	**n.a.**	**n.a.**
**IR**	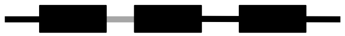	**12696**	**5653**	**65262**	**70316**	**30197**	**8654**
**ES**	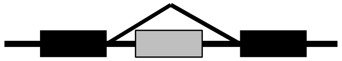	**563**	**399**	**2998**	**3977**	**1817**	**519**
**5’_ss**	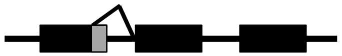	**1920**	**1248**	**4022**	**6622**	**3122**	**2840**
**3’_ss**	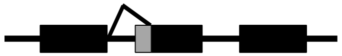	**4557**	**2532**	**3359**	**5690**	**7087**	**3807**
**5’/3’_ss**	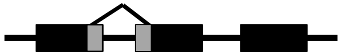	**1007**	**832**	**1100**	**2583**	**2570**	**2423**
